# Functional chimeric mRNAs encode proteins in mammalian immunity

**DOI:** 10.1038/s41586-026-10982-x

**Published:** 2026-09-02

**Authors:** Olivia Venezia, Harry Kane, Gang Du, Hannah D. Coughlan, Timothy M. Johanson, Hongshen Wang, Pathricia Tilstam, Samuel W. Kazer, Georgia Gunner, Daisy A. Hoagland, Megha G. Basavappa, Kanchana Ravichandran, Eren Ada, Ademi Zhakyp, Sandhya Kumar, Coco Duizer, Owen Searle, Chris Geo Provido, Leonel Joannas, Shilong Yang, Liam B. Healy, Qiankun Wang, Joia Capocchi, Bahawar Sharif Dhillon, Sarah Slavoff, Liang Shan, Jorge Henao-Mejia, Ruth A. Franklin, Isaac M. Chiu, Jose Ordovas-Montanes, Kate L. Jeffrey, Judy Lieberman, Rhys S. Allan, Jonathan C. Kagan, Hao Wu, Ruaidhrí Jackson

**Affiliations:** 1 Department of Immunology, Harvard Medical School, Boston, MA USA; 2 Department of Biological Chemistry and Molecular Pharmacology, Harvard Medical School, Boston, MA USA; 3 Program in Cellular and Molecular Medicine, Boston Children’s Hospital, Boston, MA USA; 4 The Walter and Eliza Hall Institute of Medical Research, Parkville, Victoria Australia; 5 Department of Medical Biology, The University of Melbourne, Parkville, Victoria Australia; 6 Broad Institute of MIT and Harvard, Cambridge, MA USA; 7 Division of Gastroenterology, Boston Children’s Hospital and Harvard Medical School, Boston, MA USA; 8 Ragon Institute of MGH, MIT and Harvard, Cambridge, MA USA; 9 Department of Stem Cell and Regenerative Biology, Harvard University, Cambridge, MA USA; 10 Moderna, Cambridge, MA USA; 11 Department of Pathology and Laboratory Medicine, Perelman School of Medicine, University of Pennsylvania, Philadelphia, PA USA; 12 Institute for Immunology, Perelman School of Medicine, University of Pennsylvania, Philadelphia, PA USA; 13 Division of Infectious Diseases, Department of Medicine, Washington University School of Medicine, St Louis, MO USA; 14 Department of Chemistry, Yale University, New Haven, CT USA; 15 Division of Protective Immunity, Department of Pathology and Laboratory Medicine, Children’s Hospital of Philadelphia, University of Pennsylvania, Philadelphia, PA USA; 16 Harvard Stem Cell Institute, Harvard University, Cambridge, MA USA; 17 The Gene Lay Institute of Immunology and Inflammation, Brigham and Women’s Hospital, Mass General Hospital and Harvard Medical School, Boston, MA USA; 18 Division of Gastroenterology, Hepatology and Nutrition, Boston Children’s Hospital, Boston, MA USA; 19 Program in Immunology, Harvard Medical School, Boston, MA USA; 20 Department of Pediatrics, Harvard Medical School, Boston, MA USA; 21 Present Address: Institute of Precision Medicine, the First Affiliated Hospital, Sun Yat-Sen University, Guangzhou, Guangdong, China; 22 Present Address: Institute of Human Immunology, Shenzhen Medical Academy of Research and Translation, Shenzhen, Guangdong, China

**Keywords:** Innate immunity, Transcriptomics

## Abstract

Individual mammalian mRNAs and proteins are typically believed to originate from single genomic loci, with isoform diversity arising through *cis*-splicing of pre-mRNA. Whether mRNA from distant genes can undergo *trans*-splicing to generate functionally relevant chimeric transcripts has remained unclear. Here we develop a pipeline combining long-read direct RNA sequencing with non-targeted and targeted validation to identify chimeric transcripts in macrophages. Chromatin conformation capture studies reveal that inflammation induces interchromosomal DNA interactions, positioning parent genes proximally to facilitate the formation of chimeric mRNA. Notably, we identify a protein-coding chimeric mRNA representing a fusion between the pore-forming protein gasdermin D (GSDMD)^1,2^ and a C-terminal domain translated out of frame from *Tmem106a* (*Gsdmd*-*Tmem106a*) in mice. We show that inflammasome priming upregulates *Gsdmd*-*Tmem106a*, with the protein localizing to the plasma membrane. After activation of the inflammasome, GSDMD–TMEM106A directly interacts with canonical GSDMD N termini to accelerate and enhance pore formation and IL-1β release. Finally, we show that GSDMD–TMEM106A balances host defence and immunopathology in vivo: its loss protects against lethal sepsis but compromises antibacterial defence, whereas overexpression enhances host protection while increasing sepsis lethality. We establish that protein-coding chimeric mRNAs formed by regulated transcript fusion events are operative during inflammation and immunity.

## Abstract (web summary)

Inflammation induces interchromosomal DNA interactions that bring parent genes into close proximity, facilitating the formation of chimeric mRNAs that encode physiologically relevant, functional proteins.

## Main

The diversity of proteins produced by cells is typically defined by the repertoire of individual genes encoded in the genome. This diversity can be amplified through pre-mRNA *cis*-splicing, where varied pairings of exons within a given transcript can be differentially fused. It is unclear whether mechanisms beyond *cis*-splicing exist to diversify the protein-coding capacity of mammalian cells. In unicellular and invertebrate organisms, the process of *trans*-splicing, whereby exons from distinct pre-mRNAs are fused to create hybrid proteins, has been reported. In trypanosomes and nematodes, a common pre-mRNA is *trans*-spliced to various other mRNAs to promote transcript stability and translation^3^; by contrast, in *Drosophila*, this process generates functionally diverse transcripts and proteins^4^. In healthy mammals, few examples of *trans*-splicing exist, and functional chimeric mRNAs (chRNAs) are typically associated with oncogenic transformation of cells where genomic translocations fuse disparate genes at the DNA level^5,6^. Expression of some chRNAs known to be produced by DNA translocation have also been described in non-malignant tissue and are proposed to form through *trans*-splicing^7–9^. However, it remains to be elucidated whether, in healthy mammalian cells, widespread fusion of mRNA from distinct genes can produce chRNAs that encode functional proteins. However, one can predict that such fusion events would expand the number of physiological protein-encoding mRNAs far beyond what is currently appreciated in our genomes.

Despite advances and widespread adoption of RNA sequencing (RNA-seq) methodologies, endogenously expressed chRNAs in mammals have largely evaded discovery because of technical limitations. cDNA synthesis-based approaches are used extensively and rely on viral reverse transcriptase enzymes to convert RNA to cDNA. However, viral reverse transcriptase enzymes can generate artificial fusion transcripts through template switching^10^. Furthermore, as mammalian chRNAs are not annotated in reference transcriptomes, candidate chRNAs multimap to different parts of the genome during alignment, resulting in their routine removal during standard RNA-seq analyses. Short-read sequencing also limits chRNA detection, as read fragments are assigned as chimeric only if they span the junction at which one gene meets the other; otherwise, these reads are assigned to the parent genes of the chRNA^11^. Moreover, additional challenges arise from difficulties in accurately resolving multimapping reads that display short junction sequences^12^ and/or map to multiple similar loci^13^. Notwithstanding these caveats, conventional RNA-seq datasets have consistently identified putative chRNAs in healthy human tissue^14,15^. Whether these chRNAs arise artificially during library preparation, or whether they are physiologically relevant, remains an open question.

## Inflammation drives chRNA expression

**Fig. 1:**
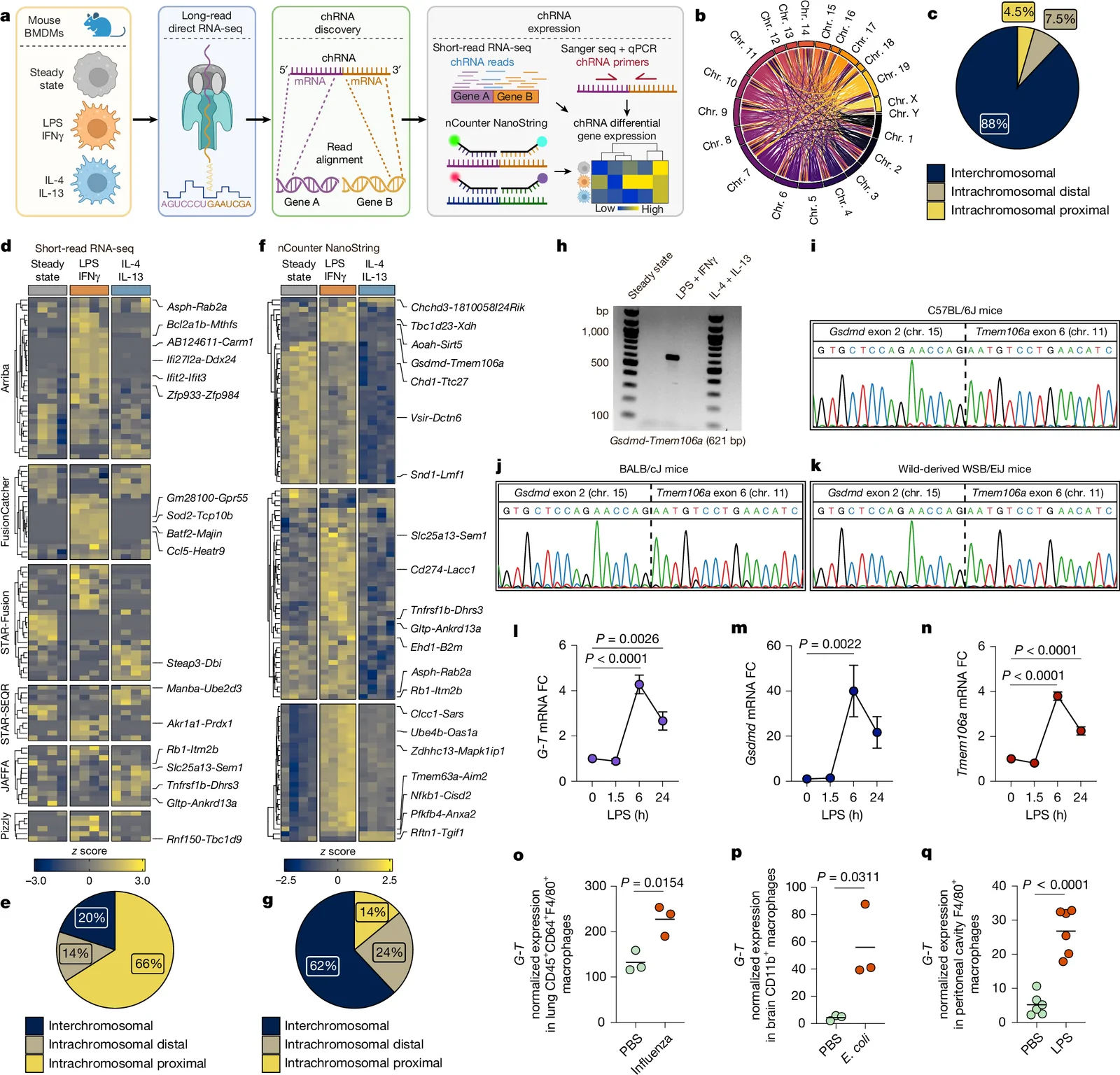
Inflammation drives chRNA expression. **a**, Schematic of chRNA identification and differential expression analysis in mouse BMDMs. The diagram was created using BioRender; Jackson, R. https://BioRender.com/3le8vsp (2026). **b**, The chromosomal distribution of 30,390 exon–exon chRNAs detected for downstream analysis. **c**, Classes of chRNA species identified using long-read RNA-seq. **d**, Scaled normalized short-read fusion fragments per million (FFPM)-like values for chRNAs co-detected in long-read and short-read RNA-seq data in BMDMs stimulated as indicated for 24 h. Selected chRNAs are highlighted. **e**, Classes of chRNA species co-detected in long-read and short-read RNA-seq. **f**, Scaled normalized counts for chRNAs using NanoString nCounter analysis in BMDMs stimulated as indicated for 24 h. Selected chRNAs are highlighted. **g**, Classes of chRNA species co-detected using NanoString nCounter and long-read RNA-seq. **h**, PCR analysis of *Gsdmd-Tmem106a* (*G-T*) cDNA expression in BMDMs. The gel image is representative of three independent experiments. **i**, Chromatogram of the *Gsdmd-Tmem106a* junction in cDNA from C57BL/6J BMDMs. For **i**–**k**, the dotted line denotes the division between *Gsdmd*- and *Tmem106a*-derived sequences. **j**,**k**, Chromatograms of the *Gsdmd-Tmem106a* junction in cDNA from BMDMs from BALB/cJ (**j**) and WSB/EiJ (**k**) mice. **l**–**n**, BMDMs were treated as indicated and the gene expression of *Gsdmd-Tmem106a* (**l**), *Gsdmd* (**m**) and *Tmem106a* (**n**) was analysed using RT–qPCR. Gene expression normalized to *Polr2a* fold change (FC) over steady state is depicted. Data are mean ± s.e.m. *n* = 5 biological replicates. **o**–**q**, RNA extracted from macrophages after infection or mock infection for NanoString nCounter analysis. The mean of normalized counts is shown. The line represents the mean; each datapoint represents a biologically independent animal. **o**, Mice were treated intranasally with influenza A virus or PBS (7 days) and CD45^+^F4/80^+^CD64^+^ cells were isolated from the lungs. **p**, Mice were infected intracisternally with *E*. *coli* or PBS (18 h) and CD11b^+^ cells were isolated from the brain. **q**, Mice were treated intraperitoneally with LPS or PBS (16 h) and F4/80^+^ cells were isolated from peritoneal lavage. *P* values were determined using one-way analysis of variance (ANOVA) (**l**–**n**) and unpaired Student’s two-tailed *t*-tests (**o**–**q**). Source data

**Extended Data Fig. 1:**
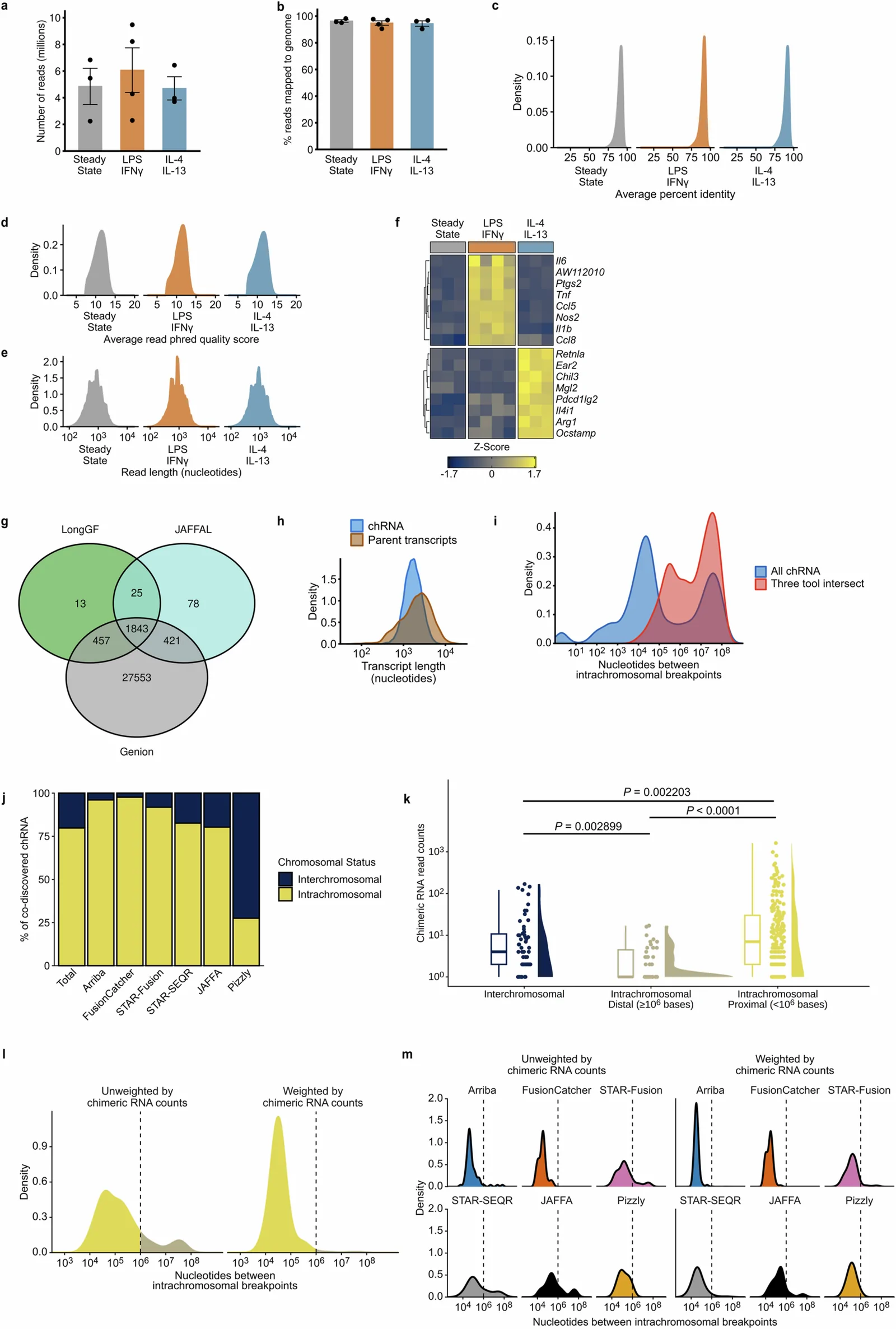
chRNA identification in long-read direct RNA-seq and short-read RNA-seq. **a**, Bar plot showing number of reads (millions) for each long-read direct RNA-seq replicate of bone marrow derived macrophages (BMDMs). BMDMs were left untreated (Steady State) or were polarized for 24 h with LPS and IFNγ or IL-4 and IL-13. **b**, Bar plot showing the percentage of reads aligning to the murine genome for the replicates in (**a**). **c**, Density plot showing average read percentage identity for the data in (**a**). Average percent identity is defined as the average base-level concordance between a read and its aligned sequence. **d**, Density plot showing average read phred quality score for the data in (**c**). **e**, Density plot showing average read length in nucleotides for the data in (**c**). **f**, Scaled normalized heatmap of Salmon pseudocounts for the data shown in **(c)**. Selected differentially expressed marker genes for macrophage polarization states are shown. **g**, Venn diagram showing overlap between exon-exon exon-repaired chRNA detected by LongGF, JAFFAL, and Genion. **h**, Density plot showing transcript lengths of the chRNA detected in (**g**) compared to the transcript lengths of the parent transcripts of these chRNA. **i**, Density plot of intrachromosomal nucleotide distances between the breakpoints of chRNA detected in (**g**), including all chRNA (blue) and chRNA co-detected by LongGF, JAFFAL, and Genion (red). **j**, Bar plot showing the percentage of interchromosomal vs intrachromosomal chRNA co-discovered in long- and short-read RNA-seq data as detected in all tools combined and by each individual short-read RNA-seq chRNA detection tool. **k**, Raincloud, dot and box plot indicating the relative short-read RNA-seq read counts for co-detected interchromosomal, distal intrachromosomal and proximal intrachromosomal reads. Significance testing was performed using the R t.test function with default settings. RNA-seq was performed on n = 12 biologically independent animals with 4 animals used per group. n = 287 chRNA. **l**, Density plots showing the distribution of nucleotides between intrachromosomal breakpoints for co-detected chRNA junctions. The densities were either unweighted (default, left-side distribution) or weighted by chRNA read counts (right-side distribution). The dotted line demarcates the 10^6^ nucleotide breakpoint on the x axis. **m**, Density plots showing the distribution of nucleotides between intrachromosomal breakpoints for co-detected chRNA junctions in each of the individual short-read chRNA detection tools. The densities were either unweighted (default, left-side distribution) or weighted by chRNA read counts (right-side distribution). The dotted lines demarcate the 10^6^ nucleotide breakpoints on the x-axes. Each data point represents a direct RNA-seq sample; all bar graphs depict mean ± s.e.m. (**a,b**). Source data

To identify chRNAs while avoiding artifacts from cDNA synthesis, we performed Oxford Nanopore PromethION direct RNA-seq analysis of polyadenylated RNA from steady-state, tissue-reparative and inflammatory mouse bone-marrow-derived macrophages (BMDMs; Fig. 1a). Ten biological replicates yielded 52.9 million passing reads (Extended Data Fig. 1a and Supplementary Table 1), of which over 90% mapped to the mouse genome (Extended Data Fig. 1b). Read identity (Extended Data Fig. 1c), average Phred scores (Extended Data Fig. 1d) and read lengths (Extended Data Fig. 1e) were consistent with high-quality published datasets, and marker-gene expression confirmed macrophage polarization states (Extended Data Fig. 1f).

The computational tools LongGF, JAFFAL and Genion^16–18^ were benchmarked to detect *BCR-ABL1* in K562 cells^19^ (Supplementary Table 2) and used to identify chRNAs containing annotated splice donor and acceptor sites from two distinct genes. This identified 30,390 candidates across activation states (Fig. 1b, Extended Data Fig. 1g and Supplementary Tables 3 and 4). These chRNAs had a median length of 1,681 nucleotides, compared with 2,190 nucleotides for their parent transcripts (Extended Data Fig. 1h). Parent genes were distributed throughout the genome (Fig. 1b), with around 88% of chRNA species arising from interchromosomal pairings (Fig. 1c) and about 4.5% and 7.5% from proximal (<10^6^ bases) and distal (≥10^6^ bases) intrachromosomal loci, respectively (Fig. 1c and Extended Data Fig. 1i), suggesting contributions from both readthrough transcription via *cis*-splicing and *trans*-splicing from distal loci.

Limited sequencing depth and modest agreement among the long-read detection tools (Extended Data Fig. 1g and Supplementary Table 1) precluded robust quantification, prompting orthogonal validation. We generated high-depth Illumina RNA-seq data (Supplementary Table 5) and analysed these data using six fusion-detection algorithms^20–24^, producing an implausibly large candidate set (Supplementary Table 6) consistent with false positives generated through reverse-transcription and PCR template switching^10^. Nevertheless, comparison with the direct RNA catalogue identified over 250 chRNAs with matching junctions (Supplementary Table 7), including candidates associated with differential macrophage polarization (Fig. 1d). Although direct RNA-seq analysis predominantly detected interchromosomal chRNAs, co-detected chRNA species were largely intrachromosomal (Fig. 1e and Extended Data Fig. 1j), with proximal species detected more frequently and supported by more reads than distal or interchromosomal species (Extended Data Fig. 1k–m), consistent with the prevalence of local and readthrough transcription^25,26^.

As short reads rarely span sufficient sequence on both sides of a chRNA junction, this approach can also generate false negatives for specific isoforms^11^. We therefore designed tandem NanoString nCounter probes that require simultaneous hybridization across each RNA junction (Fig. 1a). Over 500 probe sets were designed to target chRNAs detected by all three long-read tools (Supplementary Table 8), alongside scrambled controls with the expectation of false negatives. More than 100 high-quality probes detected constitutive or polarization-regulated chRNAs (Fig. 1f and Supplementary Table 7). Some chRNA species were validated by both Illumina and NanoString, whereas others were unique to either platform (Fig. 1d,f and Supplementary Table 7); notably, NanoString validated a greater proportion of interchromosomal species (Fig. 1e,g).

**Extended Data Fig. 2:**
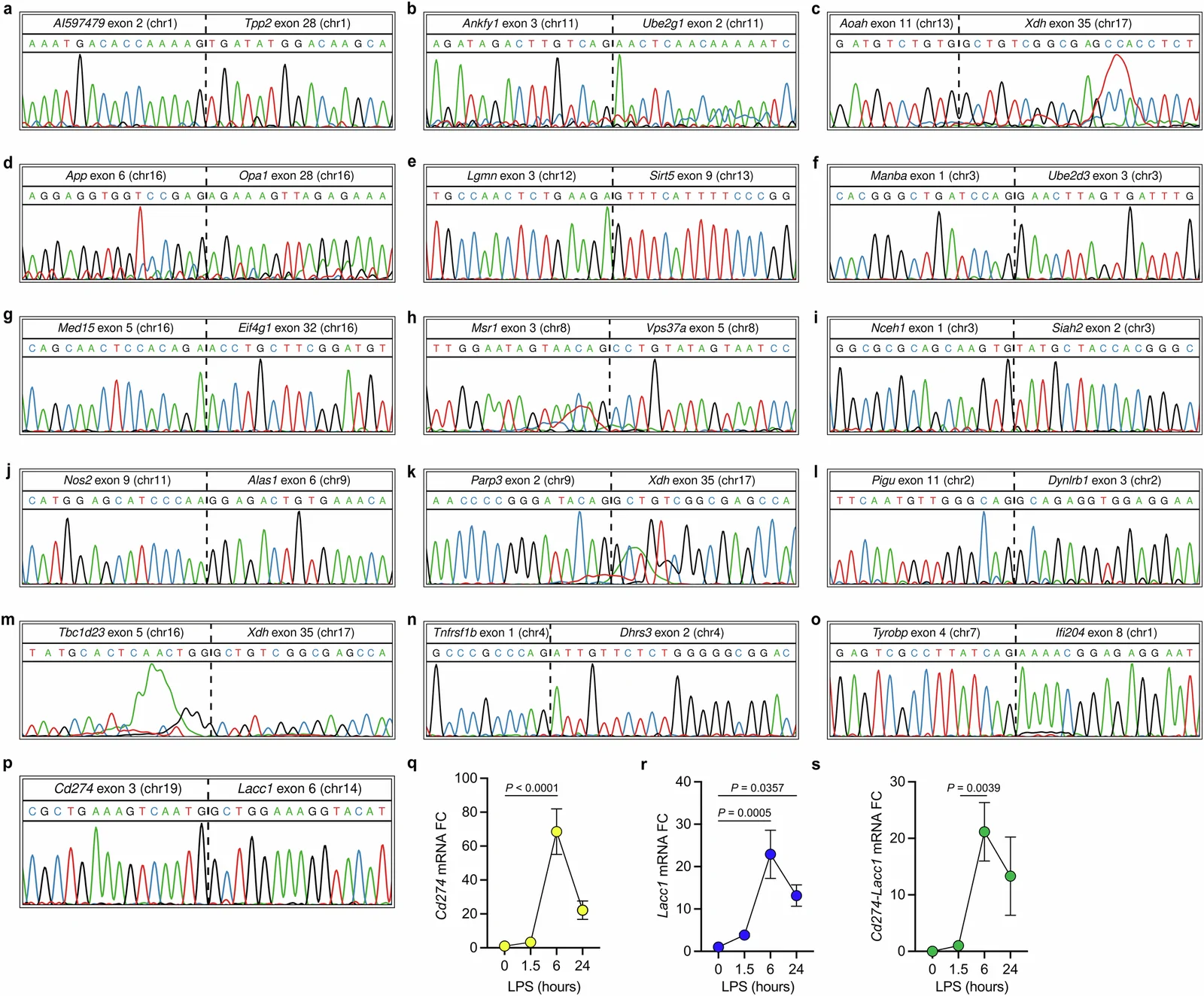
chRNA validation by PCR and Sanger Sequencing. **a-p**, Chromatograms showing the 30-nucleotide region around the junction breakpoints of (**a**) AI597479*:Tpp2*, (**b**) *Ankfy1-Ube2g1*, (**c**) *Aoah-Xdh*, (**d**) *App-Opa1*, (**e**) *Lgmn-Sirt5*, (**f**) *Manba-Ube2d3*, (**g**) *Med15-Eif4g1*, (**h**) *Msr1-Vps37a*, (**i**) *Nceh1-Siah2*, (**j**) *Nos2-Alas1*, (**k**) *Parp3-Xdh*, (**l**) *Pigu-Dynlrb1*, (**m**) *Tbc1d23-Xdh*, (**n**) *Tnfrsf1b-Dhrs3*, (**o**) *Tyrobp-Ifi204*, and (**p**) *Cd274-Lacc1* with dotted lines denoting the division between the two parent gene sequences present. RNA was extracted from murine BMDMs for cDNA synthesis and PCR using chRNA specific primers. Sanger sequencing was performed on PCR amplicons. **q-s**, BMDMs were treated with LPS for the denoted timepoints in hours, and RNA was extracted for RT-qPCR; gene expression normalized to *Polr2a* and plotted as fold change (FC) over 0 h (*Lacc1* and *Cd274*) or 1.5 h (*Cd274-Lacc1*); mean ± s.e.m.; n = 5 biologically independent animals. **q**, *Cd274* gene expression. **r**, *Lacc1* gene expression. **s**, *Cd274-Lacc1* gene expression. P value was determined by one-way ANOVA. Source data

PCR and Sanger sequencing independently validated chRNA junctions (Extended Data Fig. 2a–o and Supplementary Table 9), including a *Gsdmd-Tmem106a* amplicon spanning *Gsdmd* exons 1–2 fused to *Tmem106a* exons 6–9 (Fig. 1h–i). *Gsdmd-Tmem106a* was among the most inflammation-induced NanoString candidates (Fig. 1f), and both parent genes have established roles in lipopolysaccharide (LPS)-induced inflammation^2,27^. The same exon–exon junction was detected in C57BL/6J, BALB/cJ and wild-derived mice (Fig. 1j–k). Analysis using quantitative PCR with reverse transcription (RT–qPCR) further confirmed LPS-inducible expression, peaking at 6 h in parallel with the parent genes (Fig. 1l–n). The junction and LPS responsiveness of *Cd274-Lacc1* were similarly confirmed (Extended Data Fig. 2p–s). In vivo, *Gsdmd-Tmem106a* was detected and induced in lung macrophages after influenza A infection, brain macrophages/microglia during *Escherichia* *coli* meningitis and peritoneal macrophages after LPS administration (Fig. 1o–q).

**Extended Data Fig. 3:**
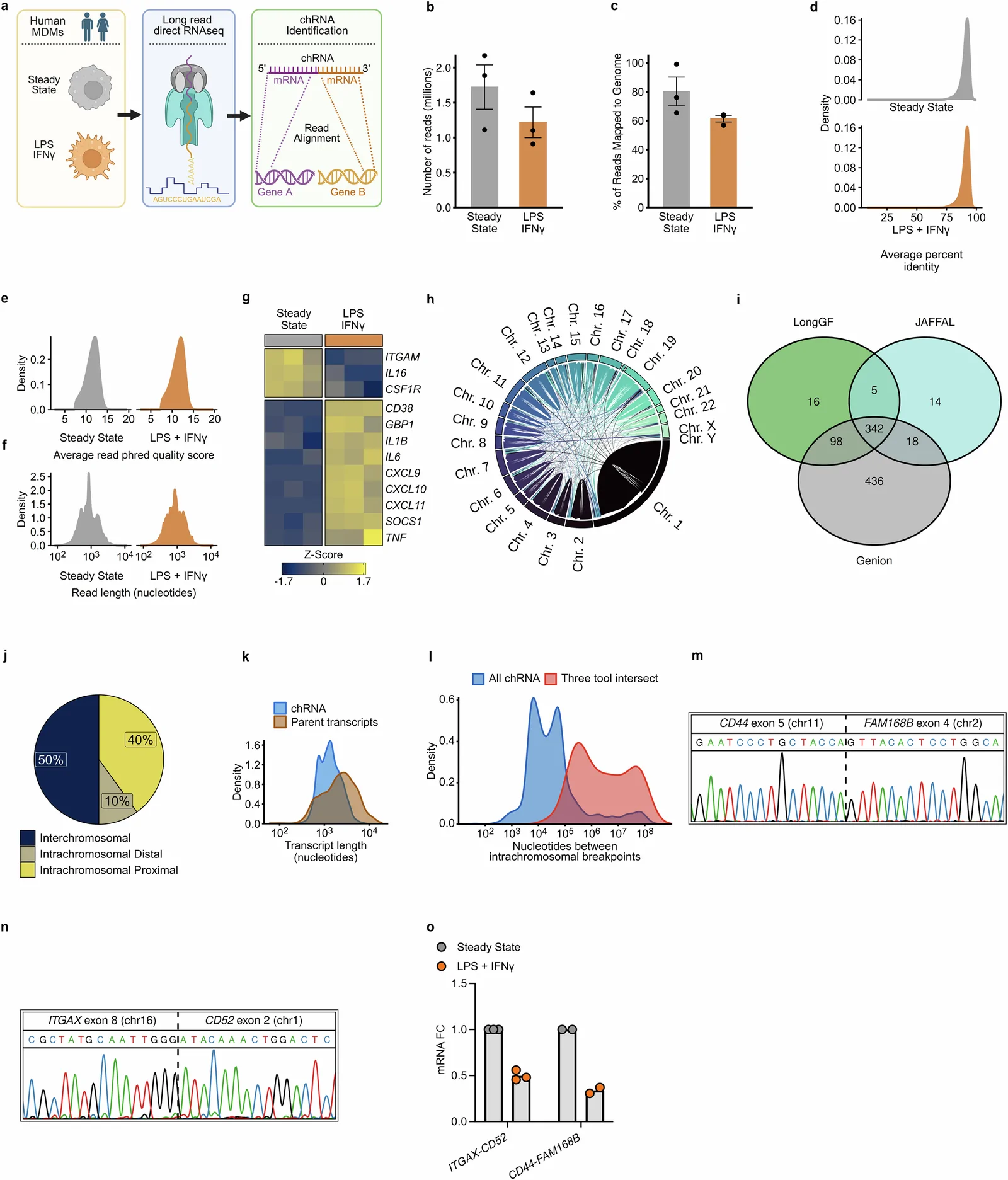
Discovery of chRNA in human innate immune cells. **a**, Schematic of long-read direct RNA-seq data generation, chimeric mRNA (chRNA) identification in human monocyte-derived macrophages (MDMs). The diagram was created using BioRender; Jackson, R. https://BioRender.com/y3l44x2 (2026). **b**, Bar plot showing number of reads (millions) for each long-read direct RNA-seq replicate of monocyte derived macrophages (MDMs). MDMs were left untreated (Steady State) or were polarized for 24 h with LPS and IFNγ. **c**, Bar plot showing the percentage of reads aligning to the human genome for the replicates in (**b**). **d**, Density plot showing average read percentage identity for the data in (**b**). Average percent identity is defined as the average base-level concordance between a read and its aligned sequence. **e**, Density plot showing average read phred quality score for the data in (**b**). **f**, Density plot showing average read length in nucleotides for the data in (**b**). **g**, Scaled normalized heatmap of Salmon pseudocounts for the data shown in (**b**). Selected differentially expressed marker genes for macrophage polarization states are shown. **h**, Chord diagram showing chromosomal distribution of 929 exon-exon chRNA (1,441 reads) used for downstream analysis. **i**, Venn diagram showing overlap between exon-exon exon-repaired chRNA detected by LongGF, JAFFAL, and Genion. **j**, Pie chart of interchromosomal versus distal and proximal intrachromosomal chRNA species. **k**, Density plot showing transcript lengths of the chRNA detected in (**i**) compared to the transcript lengths of the parent transcripts of these chRNA. **l**, Density plot of intrachromosomal nucleotide distances between the breakpoints of chRNA detected in (**i**), including all chRNA (blue) and chRNA co-detected by LongGF, JAFFAL, and Genion (red). **m-n**, Chromatograms showing the 30-nucleotide region around the junction breakpoints of *CD44-FAM168B* (**m**) *and ITGAX-CD52* (**n**), with dotted lines denoting the division between the parent gene sequences present. **o**, MDMs treated with 100 ng/mL LPS for 24 hours, and RNA extracted for RT-qPCR; gene expression normalized to *Polr2a* and plotted as fold change (FC) over steady state. Each data point represents a direct RNA-seq sample and bar graphs depict mean ± s.e.m. (**b,c**). Each data point represents a biologically independent Donor (**o**). Source data

Applying this pipeline to steady-state and inflammatory human monocyte-derived macrophages (Extended Data Fig. 3a–g and Supplementary Table 10), we identified over 900 chRNAs (Extended Data Fig. 3h–i and Supplementary Table 11), including inter- and intrachromosomal species (Extended Data Fig. 3j) with properties resembling those of mouse chRNAs (Extended Data Fig. 3k–l). qPCR further confirmed their detection and differential regulation by LPS (Extended Data Fig. 3m–o).

**Extended Data Fig. 4:**
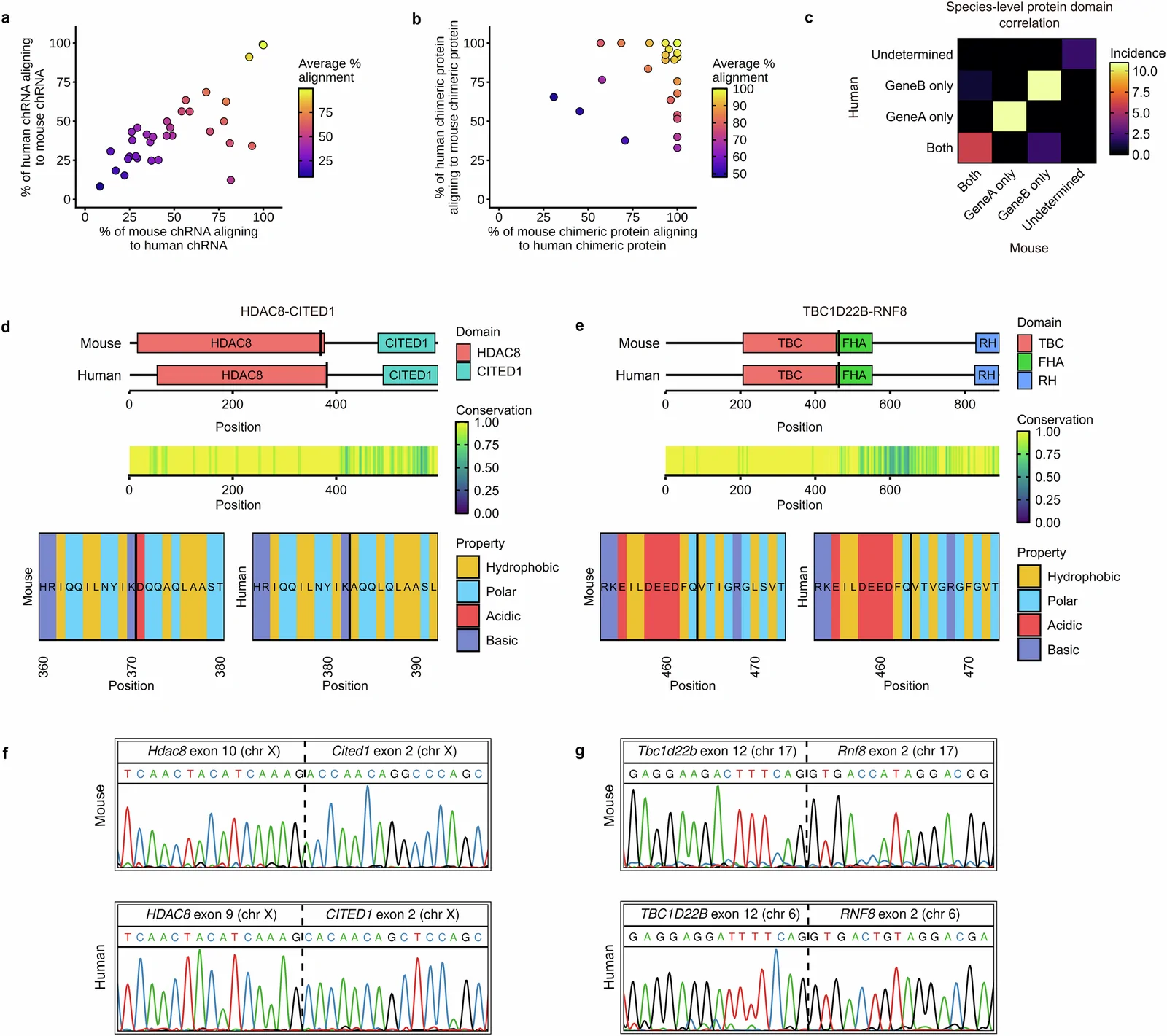
Discovery of conserved murine and human chRNA. **a**, Scatter plot showing percentage blast alignment of matched conserved murine versus human chRNA transcripts. **b**, Scatter plot showing percentage blast alignment of matched conserved murine versus human chimeric proteins. **c**, Species-level protein domain correlation heatmap of domain blast mapping in murine and human chimeric proteins. **d**, Comparative protein analysis plot of murine and human *HDAC8-CITED1*. Top panel shows the domain structure of the chimeric proteins with thick vertical black lines indicating the chRNA breakpoints. Middle panel shows an amino acid conservation heatmap. Bottom panel shows junction plots of amino acids either side of the chimeric protein junction. **e**, Comparative protein analysis plot of murine and human *TBC1D22B-RNF8*. Top panel shows the domain structure of the chimeric proteins with thick vertical black lines indicating the chRNA breakpoints. Middle panel shows an amino acid conservation heatmap. Bottom panel shows junction plots of amino acids located either side of the chimeric protein junction. **f**, Chromatogram showing the 30-nucleotide regions around the mouse (top) and human (bottom) *Hdac8-Cited1* and *HDAC8-CITED1* chimeric junctions, with dotted lines denoting the division between the parent gene sequences present. Mouse *Hdac8* exon 10 corresponds to human *HDAC8* exon 9. n = 33 conserved pairs (**a**,**b**).**g**, Chromatogram showing the 30-nucleotide regions around the mouse (top) and human (bottom) *Tbc1d22b-Rnf8* and *TBC1D22B-RNF8* chimeric junctions, with dotted lines denoting the division between the parent gene sequences present. Source data

Despite lower sequencing depth in the human dataset (Extended Data Fig. 3b and Supplementary Table 10), cross-species analysis identified 33 chRNAs sharing parent genes between mice and humans (Extended Data Fig. 4a and Supplementary Table 12). Although we did not identify a *GSDMD-TMEM106A* chRNA in these cells, approximately 8 displayed at least 50% mRNA sequence homology and 28 encoded predicted proteins with at least 50% conservation (Extended Data Fig. 4a,b and Supplementary Table 12), accompanied by closely matched domain architectures (Extended Data Fig. 4c). These included *HDAC8-CITED1* and *TBC1D22B-RNF8*, which retained functional domains from both parent genes (Extended Data Fig. 4d–e) and were validated by Sanger sequencing in mouse and human macrophages (Extended Data Fig. 4f–g).

Thus, integrating direct RNA-seq with short-read RNA-seq, NanoString, PCR and expression analyses provides a generalizable framework for identifying, validating and prioritizing endogenous chRNAs for functional studies in immunity and inflammation.

## Inflammation controls chRNA gene contact

**Extended Data Fig. 5:**
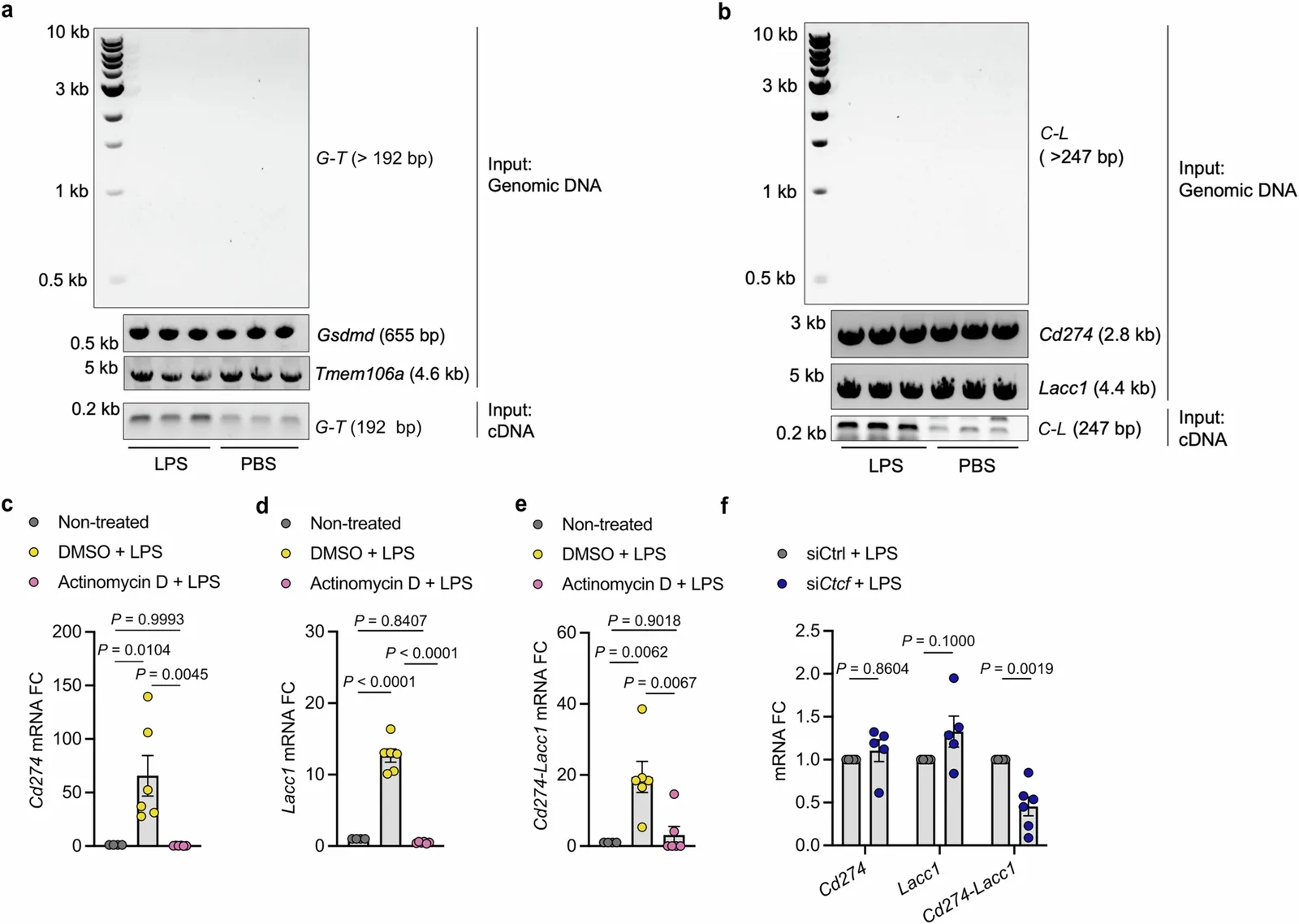
chRNA are derived from nascent mRNA not chromosomal translocation. **a-b**, Genomic DNA was isolated from non-treated and LPS stimulated BMDMs and long-range genomic PCRs were performed. **a**, PCR was performed using a forward primer in exon 2 of *Gsdmd* and a reverse primer in exon 6 of *Tmem106a (G-T)*. For controls, exon spanning primers for native *Gsdmd* and *Tmem106a* were used, as well as the same *Gsdmd-Tmem106a* junction primers with cDNA input. **b**, PCR was performed using a forward primer in exon 3 of *Cd274* and a reverse primer in exon 6 of *Lacc1 (C-L)*. For controls, exon spanning primers for native *Cd274* and *Lacc1* were used, as well as the same *Cd274-Lacc1* junction primers with cDNA input. PCR gel images are representative of two independent experiments. **c-e**, BMDMs left non-treated or treated with LPS and DMSO or Actinomycin D for 3 h. RNA extracted for RT-qPCR; gene expression of (**c**) *Cd274*, (**d**) *Lacc1*, and (**e**) *Cd274-Lacc1* normalized to *Polr2a* and plotted as fold change (FC) over non-treated. n = 4 non-treated, n = 6 DMSO + LPS and Actinomycin D + LPS. **f**, BMDMs were transfected with siRNA targeting *Ctcf* (si*Ctcf*) (n = 5 mice *Cd274* and *Lacc1* or n = 6 mice *Cd274-Lacc1*) or non-targeting siRNA (siCtrl) (n = 5 mice all genes). RT-qPCR of LPS stimulated (100 ng/ml) BMDMs; gene expression normalized to *Polr2a* and plotted as fold change (FC) over siCtrl. Each data point represents a biologically independent animal; bar graphs depict mean ± s.e.m.; one-way ANOVA (**c-e**) and two-way ANOVA (**f**) were used for analysis. For gel source data see Supplementary Fig. 1 (**a-b**). Source data

To distinguish chromosomal translocation from RNA-level fusion, we performed long-range genomic PCR analysis of DNA from untreated and LPS-stimulated BMDMs using primers positioned in the junction exons, including *Gsdmd* exon 2 and *Tmem106a* exon 6. Neither *Gsdmd-Tmem106a* nor *Cd274-Lacc1* produced the expected kilobase-scale genomic amplicon under either condition (Extended Data Fig. 5a,b), arguing against detectable chromosomal translocations and supporting RNA-level fusion through *trans*-splicing.

**Fig. 2:**
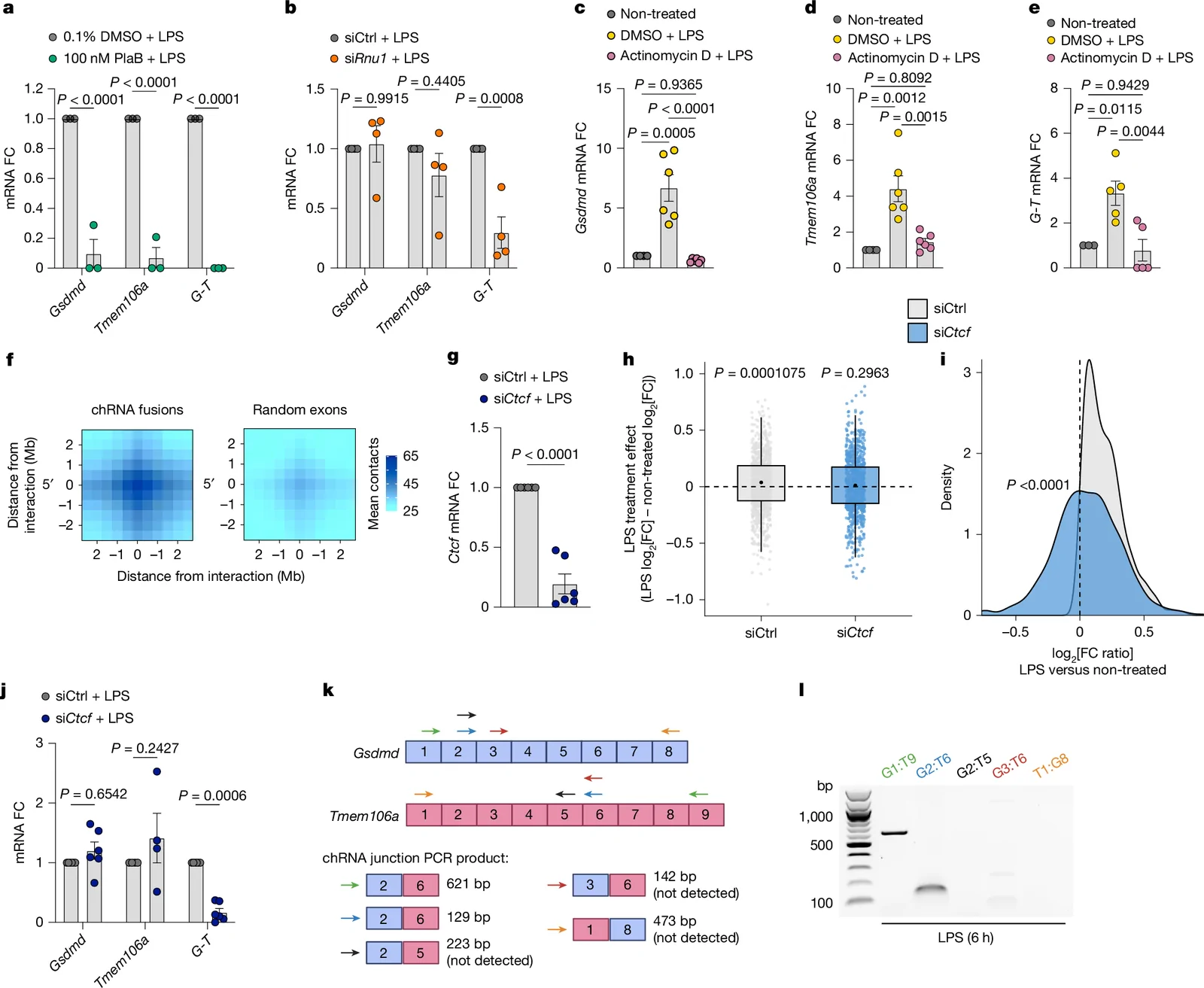
Inflammation controls chRNA gene contact. **a**, BMDMs were treated with LPS for 6 h in the presence of DMSO or pladienolide B (PlaB) and analysed using RT–qPCR. **b**, BMDMs were transfected with non-targeting (siCtrl) or *Rnu1*-targeting (si*Rnu1*) siRNA, treated with LPS for 6 h and analysed using RT–qPCR. *n* = 4 mice per group. **c**–**e**, BMDMs were left non-treated (*n *= 4 mice) or treated with LPS and DMSO or actinomycin D for 3 h (*n* = 5 (*Gsdmd-Tmem106a*) (*G*-*T*) and *n* = 6 (*Gsdmd* and *Tmem106a*) mice per group). The relative expression of *Gsdmd* (**c**), *Tmem106a* (**d**) and *Gsdmd-Tmem106a* (**e**) was analysed using RT–qPCR. **f**, Aggregate peak analysis (APA) of in situ Hi-C contacts between paired exons of interchromosomal chRNAs or random exons. **g**, RT–qPCR analysis of BMDMs that were transfected with siCtrl or *Ctcf*-targeting siRNA (si*Ctcf*) and treated with LPS for 6 h.* n* = 6 mice per group. **h**, LPS-induced changes in the contact frequency at chRNA interaction sites in siCtrl- and si*Ctcf*-transfected BMDMs. For the box plots, the central dot represents the median, the upper and lower hinges represent the 25th and 75th percentiles, and the upper and lower whiskers extend to 1.5× the interquartile range (IQR) of the upper and lower hinges, respectively. **i**, Density plot of log_2_[fold change (FC) ratio] interactions with FC ratios > 1 in LPS-stimulated siCtrl BMDMs. Ratios were calculated by dividing the LPS-stimulated FC by the corresponding unstimulated FC for each siRNA condition with log_2_[FC ratio] values plotted. **j**, RT–qPCR analysis of LPS-stimulated BMDMs that were transfected with siCtrl or si*Ctcf*. *n* = 6 (*Gsdmd* and *Gsdmd-Tmem106a*) and *n* = 4 (*Tmem106a*) mice per group. **k**, The primer sets used to amplify alternative *Gsdmd-Tmem106a* exon combinations. The diagram was created using BioRender; Venezia, O. https://BioRender.com/xvepcox (2026). **l**, Gel electrophoresis of LPS-treated BMDM cDNA using primers shown in **k**. The gel image is representative of two independent experiments. RT–qPCR expression was normalized to *Polr2a* and plotted as fold change (FC) over the indicated control. Each datapoint represents a biologically independent animal. For all bar graphs, data are mean ± s.e.m. (**a**–**e**,** g** and **j**). Hi-C data were combined from three independent biological replicates (**f**, **h** and **i**). *P* values were calculated using paired two-tailed *t*-tests (**h**), two-sided Kolmogorov–Smirnov tests (**i**), one-way ANOVA (**c**–**e**), two-way ANOVA (**a**,** b** and **j**) and unpaired Student’s two-tailed *t*-tests (**g**). Source data

As our chRNA catalogue was defined using annotated splice sites, we tested whether canonical splicing machinery contributes to chRNA formation. Inhibition of the SF3B spliceosome complex with pladienolide B^28,29^ or knockdown of U1 small nuclear RNA abolished LPS-induced *Gsdmd-Tmem106a* expression (Fig. 2a,b). Inhibition of RNA polymerase II with actinomycin D^30^ similarly eliminated *Gsdmd-Tmem106a* and *Cd274-Lacc1*, as well as their parent transcripts (Fig. 2c–e and Extended Data Fig. 5c–e), supporting the co-transcriptional joining of newly synthesized RNAs.

We next examined whether chRNA formation is facilitated by spatial proximity between parent genes. Genome-wide in situ Hi-C analysis revealed that interchromosomal parent–gene pairs identified by direct RNA-seq (Extended Data Fig. 1g) were significantly enriched for DNA–DNA interactions relative to randomized exon pairs (Fig. 2f). As CTCF regulates genome organization^31^, we depleted *Ctcf* using small interfering RNA (siRNA; Fig. 2g) and performed Hi-C after 6 h of LPS stimulation. LPS enhanced contacts between chRNA parent genes, whereas *Ctcf* depletion abolished this increase, demonstrating that these interactions are dynamically regulated and CTCF dependent (Fig. 2h,i).

Functionally, *Ctcf* depletion reduced LPS-induced *Gsdmd-Tmem106a* without affecting *Gsdmd* or *Tmem106a* expression (Fig. 2j), and similarly reduced *Cd274-Lacc1* without altering either parent gene (Extended Data Fig. 5f). Finally, junction-specific primers (Fig. 2k) detected only the *Gsdmd* exon 2 and *Tmem106a* exon 6 junction isoform (Fig. 2l), demonstrating that fusion is highly selective rather than a random consequence of proximity. Together, these findings show that inflammation promotes CTCF-dependent interactions between chRNA parent genes, enabling specific *trans*-splicing and chRNA formation in macrophages.

## *Gsdmd-Tmem106a* encodes a fusion protein

**Fig. 3:**
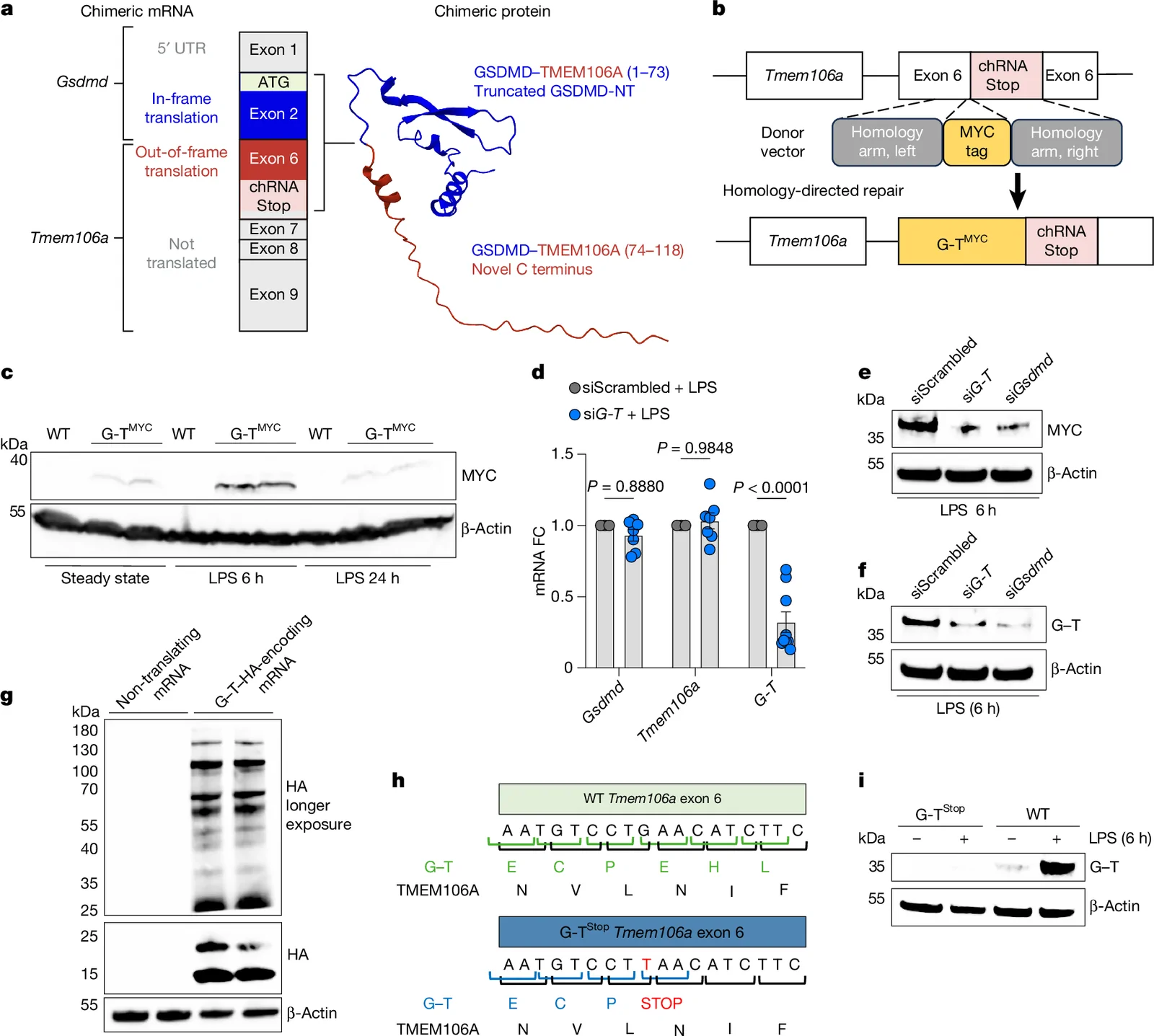
*Gsdmd-Tmem106a* encodes a fusion protein. **a**, Schematic of the *Gsdmd-Tmem106a* mRNA exon composition and open reading frame (the *Gsdmd-*encoded portion is shown in blue and the *Tmem106a*-encoded portion is shown in red) and AlphaFold 3 protein prediction. The diagram was created using BioRender; Venezia, O. https://BioRender.com/ttwt0ch (2026). **b**, Schematic of the design of the GSDMD–TMEM106A–MYC-tag knock-in (G-T^MYC^) mouse using CRISPR–Cas9. **c**, BMDMs isolated from wild-type (WT) or G-T^MYC^ mice were left unstimulated (steady state) or treated with LPS for 6 or 24 h. Western blotting of MYC was performed on cell lysates. **d**, RT–qPCR analysis of LPS-stimulated (6 h) BMDMs transfected with *Gsdmd-Tmem106a* scrambled control siRNA (siScrambled) or siRNA against *Gsdmd-Tmem106a* (si*G-T*); the relative gene expression over *Polr2a* normalized to siScrambled is shown. Each datapoint represents a biologically independent animal. Data are mean ± s.e.m. *n* = 3 (siScrambled, all genes), *n* = 7 (si*G-T*, *Gsdmd* and *Tmem106a*) and *n* = 9 (siG-T, *Gsdmd-Tmem106a*). **e**,**f**, BMDMs derived from G-T^MYC^ mice were transfected with siScrambled control, si*G-T* or si*Gsdmd* and treated with LPS. Western blotting of MYC tag (**e**) or GSDMD–TMEM106A (**f**) using an anti-GSDMD–TMEM106A (G–T) antibody was performed on cell lysates. **g**, BMDMs were transfected with non-translating mRNA or mRNA encoding GSDMD–TMEM106A with a C-terminal HA tag (G–T–HA). Western blotting of HA-tagged protein was performed on cell lysates. **h**, Schematic of the point mutation used to design G-T^Stop^ mice. **i**, BMDMs were generated from G-T^Stop^ and wild-type mice and treated with PBS or LPS. Western blot analysis of GSDMD–TMEM106A using anti-GSDMD–TMEM106A was performed on cell lysates. All blots are representative of two independent experiments. β-Actin was used as the loading control. Statistical analysis was performed using two-way ANOVA (**d**). Gel source data are provided in Supplementary Fig. 2. Source data

To test chRNA protein-coding potential, we focused on *Gsdmd-Tmem106a*, which retains the *Gsdmd* 5′ untranslated region and canonical start codon. The *Tmem106a*-derived sequence was predicted to be translated out of frame, producing a novel C terminus that is absent in the annotated mouse proteome (Fig. 3a).

**Extended Data Fig. 6:**
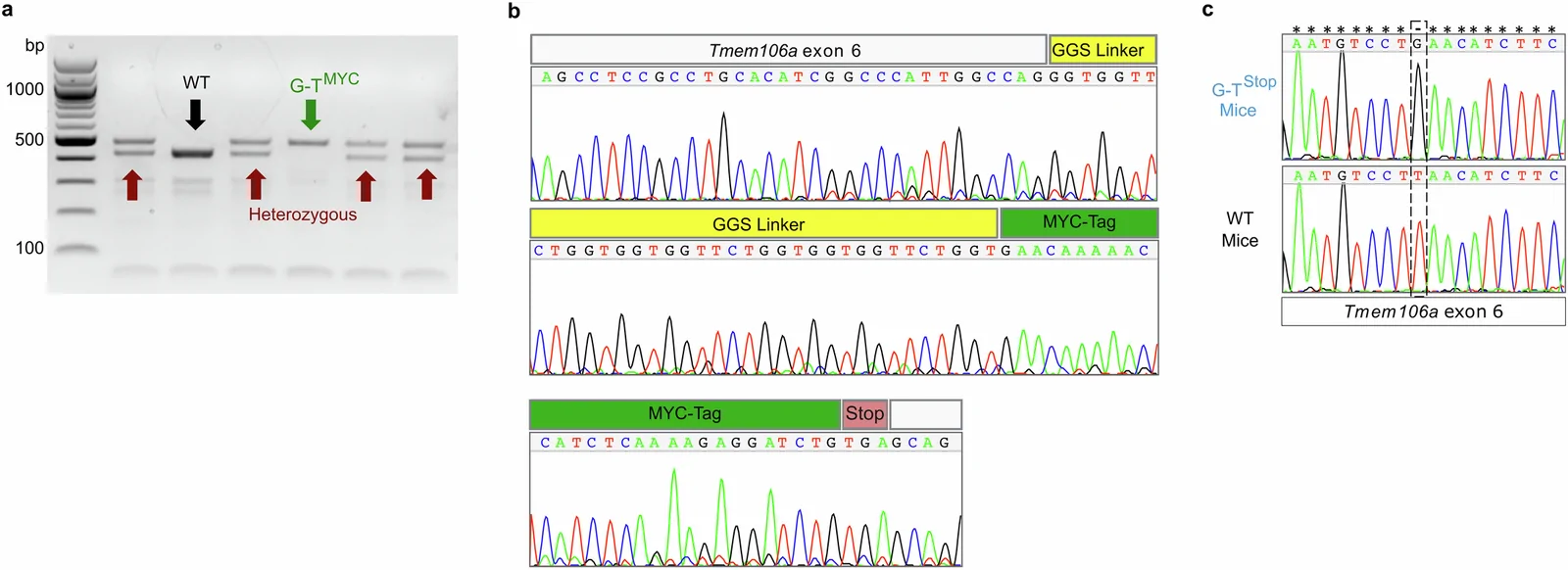
Generation of G-T^MYC^ and G-T^Stop^ mice. **a**, PCR and gel electrophoresis of G-T^MYC^ mice. Knock-in depicted by G-T^MYC^ in green (homozygous) and red (heterozygous) and wild-type (WT) labelled in black. Image is representative of three independent experiments. **b**, Chromatogram of G-T^MYC^ knock-in gel purified PCR product. **c**, Chromatogram depicting successful generation of G-T^Stop^ mice with a G to T mutation in exon 6 of *Tmem106a*.

We generated *Gsdmd-Tmem106a*-MYC knock-in mice (G-T^MYC^ mice) carrying a C-terminal MYC tag within the chimeric ORF located in exon 6 of *Tmem106a* before the predicted chimeric stop codon (Fig. 3b and Extended Data Fig. 6a,b). As the *Tmem106a*-derived sequence would be translated out of frame, the tag is exclusive to GSDMD–TMEM106A. MYC immunoblotting detected an LPS-inducible protein in G-T^MYC^ BMDMs but not in wild-type BMDMs (Fig. 3c). A junction-specific siRNA selectively depleted *Gsdmd-Tmem106a* (Fig. 3d), while an independent siRNA targeting *Gsdmd* exon 2 (encoded on chromosome 15) was used to test whether this *Gsdmd* exon is required to produce the chromosome-11-encoded MYC tag. Both reduced the MYC signal, confirming its chimeric origin (Fig. 3e).

A custom antibody against the novel C-terminal peptide similarly detected an LPS-inducible protein diminished by both siRNAs (Fig. 3f). The detected endogenous GSDMD–TMEM106A migrated above its predicted molecular mass, whereas overexpressed GSDMD–TMEM106A with a C-terminal HA tag produced both the expected approximately 15 kDa protein and higher-molecular-mass species (Fig. 3g), suggesting multiple modified states.

Finally, we generated *Gsdmd-Tmem106a*^Stop^ (G-T^Stop^) mice by introducing a single G-to-T substitution into *Tmem106a* exon 6 (Extended Data Fig. 6c). This created a stop codon after the third *Tmem106a*-derived amino acid without altering canonical GSDMD or TMEM106A translation (Fig. 3h). GSDMD–TMEM106A was absent from G-T^Stop^ BMDMs but robustly induced by LPS in wild-type cells (Fig. 3i), establishing that *Gsdmd-Tmem106a* encodes a stable, LPS-inducible protein.

## GSDMD–TMEM106A drives IL-1β immunity

**Fig. 4:**
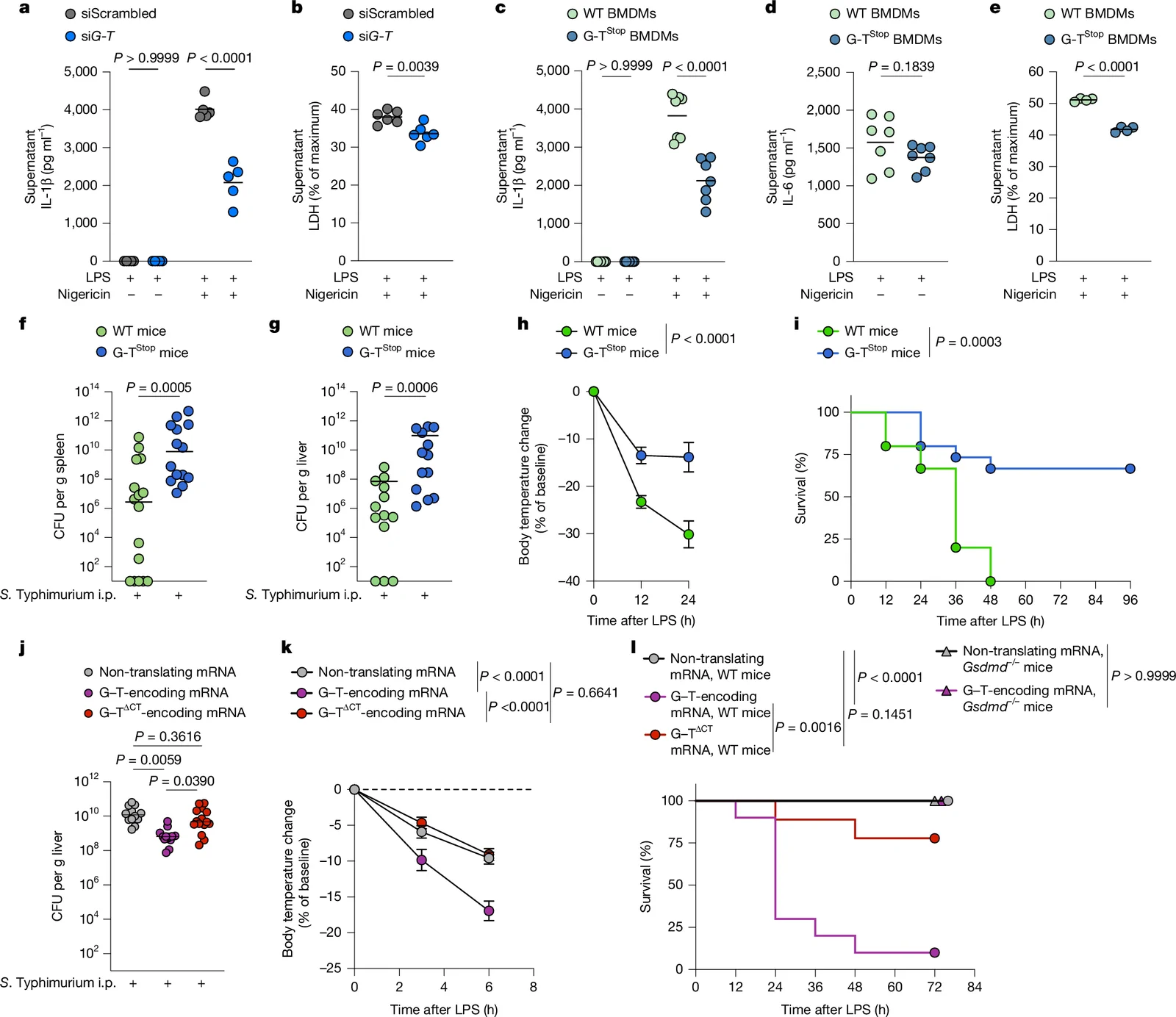
GSDMD–TMEM106A drives IL-1β immunity. **a**,**b**, BMDMs were transfected with siRNA targeting *Gsdmd-Tmem106a* (si*G-T*) or scrambled siRNA control and treated with LPS and nigericin. **a**, Enzyme-linked immunosorbent assay (ELISA) of BMDM culture supernatants 30 min after nigericin treatment. *n* = 5 mice per group. **b**, Measurement of LDH in BMDM supernatants 2 h after treatment with nigericin. **c**–**e**, BMDMs were generated from G-T^Stop^ and wild-type (WT) mice and treated with LPS and nigericin. **c**, ELISA analysis of BMDM supernatants 30 min after nigericin.* n *= 7 mice per group. **d**, ELISA was performed on supernatants after LPS priming with no nigericin. **e**, Measurement of LDH in BMDM supernatants at 2 h after treatment with nigericin. **f**,**g**, G-T^Stop^ and wild-type mice were infected with *S*. Typhimurium through intraperitoneal (i.p.) injection (10^2^ colony-forming units (CFU)). Then, 24 h later, spleens (**f**) and livers (**g**) were collected for colony count (CFUs) analysis. **h**,**i**, G-T^Stop^ and wild-type mice were administered LPS (15 mg per kg) intraperitoneally, and the body temperature (**h**) was measured and mice were assessed for signs of morbidity (**i**). *n* = 15 mice per group. **j**–**l**, Mice received the indicated LNP-encapsulated mRNA. **j**, At 18 h after mRNA delivery, *S*. Typhimurium was injected intraperitoneally (10^5^ CFU). Then, 24 h later, livers were collected for CFU counts. *n* = 12 (non-translating), *n* = 13 (*Gsdmd-Tmem106a* mRNA) and *n* = 15 (G-T^ΔCT^ mRNA) mice per group. **k**, At 18 h after mRNA delivery, LPS was injected intraperitoneally (3 mg per kg) and the body temperature was measured. *n* = 19 (non-translating and G-T) or *n* = 18 (G-T^ΔCT^) mice per group. **l**, At 18 h after mRNA delivery, LPS was injected intraperitoneally (6 mg per kg) and mice were assessed for signs of morbidity. Experiments using *Gsdmd*^*−/−*^ mice were completed separately. *n* = 9 (wild-type non-translating and G-T^ΔCT^), *n *= 10 (wild-type G-T), *n* = 6 (*Gsdmd*^*−/− *^G-T), *n* = 5 (*Gsdmd*^–/–^ non-translating) mice. Data are the mean of three independent experiments. Each datapoint represents a biologically independent animal, with the mean bar depicted (**a**–**g** and **j**). For **h** and **k**, data are mean ± s.e.m. Pairwise comparisons were performed using unpaired two-tailed *t*-tests (**b**, **d** and **e**) or two-tailed Mann–Whitney *U*-tests (**f** and **g**). For **a**, **c** and **h**–**l**, *P* values were calculated using one-way ANOVA (**j**), two-way ANOVA (**a**,** c**, **h** and** k**) and Kaplan–Meier with Mantel–Cox test (**i** and **l**). Source data

**Extended Data Fig. 7:**
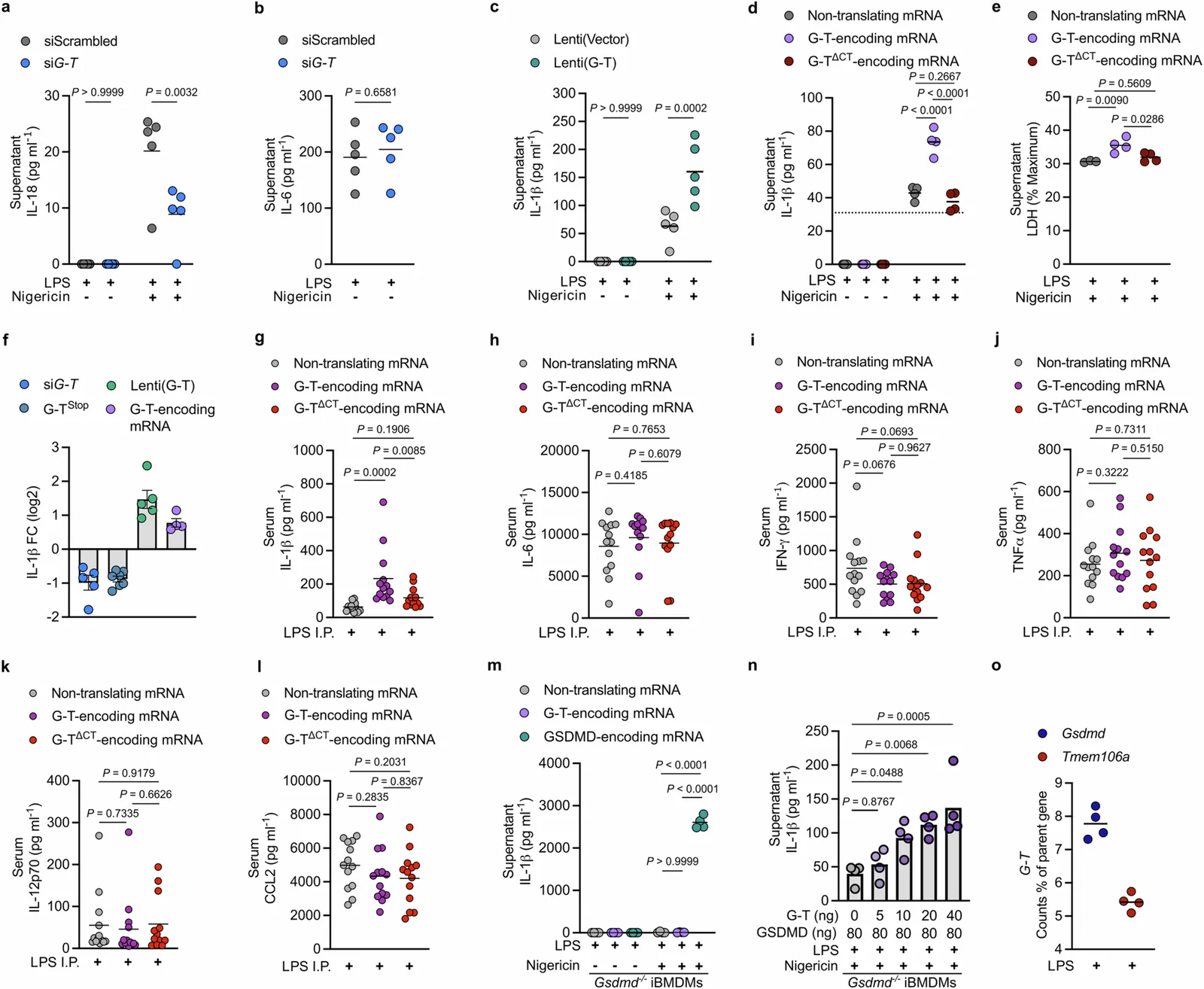
G-T selectively enhances IL-1β release. **a-b**, BMDMs were transfected with siRNA targeting *Gsdmd-Tmem106a* (si*G-T*) or scrambled (siScrambled) siRNA. **a**, BMDMs were treated with LPS (10 ng/ml; 6 h) and nigericin (0 or 10 μM; 30 min) and ELISA was performed on cell culture supernatant. n = 5 mice per group. **b**, BMDMs were treated with LPS (10 ng/ml; 6 h) and ELISA was performed on cell culture supernatant. **c**, ELISA of BMDM culture supernatant following transduction with lentivirus expressing *Gsdmd-Tmem106a* (Lenti(G-T)) or empty vector (Lenti(Vector)) and treatment with LPS (100 ng/ml; 6 h) and nigericin (10 μM; 30 min). n = 5 mice per group. **d-e**, Wild-type BMDMs transfected with mRNA encoding GSDMD-TMEM106A protein (G-T), G-T^ΔCT^ or a non-translating mRNA and treated with LPS (100 ng/ml) and nigericin (0 or 10 μM). n = 5 mice per group. **d**, ELISA was performed on cell culture supernatant 30 min after nigericin treatment. Dashed line indicates limit-of-detection for cytokine ELISA assay. **e**, Measurement of LDH in BMDM culture supernatant 2 h post-nigericin treatment. **f**, Log2 fold change (FC) analysis of IL-1β secretion across different G-T depletion and overexpression models; mean ± s.e.m. **g-l**, Mice received LNP encapsulated mRNA encoding GSDMD-TMEM106A (G-T), G-T^Δ^^CT^ or a non-translating control via tail-vein injection (1 mg/kg). 18 h after mRNA delivery, LPS was injected intraperitoneally (3 mg/kg) to induce sepsis. 6 h after LPS treatment, serum was collected for cytokine measurement. **m**, ELISA of *Gsdmd*^−/−^ iBMDM culture supernatant following transfection of mRNA encoding G-T, GSDMD, or a non-translating mRNA and treatment with LPS (100 ng/ml) for 6 h and nigericin (10 μM) for 30 min. n = 4 per group. **n**, ELISA of *Gsdmd*^−/−^ iBMDM supernatant following mRNA transfection with mRNA encoding GSDMD and titrated amounts of G-T, as well as treatment with LPS and nigericin. **o**, NanoString nCounter data showing *Gsdmd-Tmem106a* (*G-T*) normalized counts as a percentage of *Gsdmd* or *Tmem106a* normalized counts. Each data point represents a biologically independent animal (**a-l,o**) or cells (**m,n**) with mean bar depicted. Unpaired two-tailed t test (**b**) was used for pairwise comparison. One-way ANOVA (**e,g-l,n**) and two-way ANOVA (**a,c,d,m**) were used for analysis. Source data

To define GSDMD–TMEM106A function, we examined its contribution to inflammasome activity. Canonical GSDMD is cleaved by inflammatory caspases, releasing GSDMD-NT to oligomerize and form membrane pores that enable secretion of caspase-1 (CASP-1)-processed IL-1β and IL-18 (refs. ^1,2,32^). After siRNA-mediated *Gsdmd-Tmem106a* knockdown (Fig. 3d), LPS- and nigericin-induced IL-1β and IL-18 release was significantly reduced (Fig. 4a and Extended Data Fig. 7a). LDH release after prolonged nigericin treatment was also reduced, although less markedly than early cytokine secretion (Fig. 4b), whereas non-inflammasome-dependent IL-6 secretion was unaffected (Extended Data Fig. 7b).

BMDMs from G-T^Stop^ mice similarly displayed reduced early IL-1β release but normal IL-6 secretion (Fig. 4c,d), with a modest reduction in late-stage cell lysis (Fig. 4e). Thus, both acute depletion and genetic loss of GSDMD–TMEM106A selectively impair inflammasome-dependent cytokine release and pyroptosis.

As IL-1β promotes antibacterial immunity^33,34^ and GSDMD protects against *Salmonella enterica* subspecies *enterica* serovar Typhimurium (hereafter, *S*. Typhimurium)^35^, we challenged G-T^Stop^ and wild-type mice with *S*. Typhimurium. G-T^Stop^ mice exhibited significantly greater bacterial burdens in the spleen and liver compared with their co-housed littermates (Fig. 4f,g). Conversely, in lethal LPS-induced sepsis, in which GSDMD contributes to disease severity^32^, G-T^Stop^ mice were protected from early hypothermia (Fig. 4h) and, whereas the majority of G-T^Stop^ mice survived for 96 h, all of the wild-type controls succumbed within 48 h (Fig. 4i).

Gain-of-function experiments produced reciprocal effects. Lentiviral GSDMD–TMEM106A overexpression increased IL-1β release after NLRP3 activation (Extended Data Fig. 7c). Similarly, transfection with *Gsdmd-Tmem106a* mRNA enhanced IL-1β secretion and accelerated LDH release, whereas mRNA encoding only the *Gsdmd*-derived region (*Gsdmd*-*Tmem106a*^ΔCT^ (G-T^ΔCT^-encoding mRNA)) did not (Extended Data Fig. 7d–e), demonstrating that the *Tmem106a*-derived sequence is required. Although absolute IL-1β release differed between overexpression and loss-of-function approaches, the relative effects were consistent across models (Extended Data Fig. 7f).

We next administered LNP-encapsulated *Gsdmd-Tmem106a*, *Gsdmd-Tmem106a*^ΔCT^ or non-translating control mRNA 18 h before *S.* Typhimurium challenge. *Gsdmd-Tmem106a* administration significantly reduced liver bacterial burden at 24 h compared with both controls (Fig. 4j), demonstrating that exogenous GSDMD–TMEM106A enhances antibacterial defence. However, in LPS-induced sepsis, *Gsdmd*-*Tmem106a* administration accelerated hypothermia within 3 h compared with *Gsdmd-Tmem106a*^ΔCT^ and control mRNAs (Fig. 4k). As inflammasome cytokines and GSDMD activity promote sepsis lethality^32^, we measured serum cytokines 6 h after LPS. *Gsdmd-Tmem106a* specifically increased IL-1β (Extended Data Fig. 7g) without altering IL-6, IFNγ, TNF, CCL2 or IL-12p70 (Extended Data Fig. 7h–l). Nearly all *Gsdmd-Tmem106a*-treated mice subsequently reached the humane end points within 72 h, whereas *Gsdmd-Tmem106a*^ΔCT^ and control-treated mice were protected (Fig. 4l).

Finally, because *Gsdmd*^*−/−*^ mice resist lethal LPS sepsis^32^, we tested whether *Gsdmd-Tmem106a* requires endogenous GSDMD. *Gsdmd-Tmem106a*-induced sepsis lethality was completely abolished in *Gsdmd*^*−/−*^ mice (Fig. 4l), and *Gsdmd-Tmem106a* overexpression did not induce IL-1β release in *Gsdmd*^*−/−*^ immortalized BMDMs (iBMDMs) (Extended Data Fig. 7m). Thus, GSDMD–TMEM106A functions through canonical GSDMD to amplify IL-1β release, enhancing antibacterial defence while exacerbating sepsis immunopathology.

## GSDMD–TMEM106A enhances pore formation

**Fig. 5:**
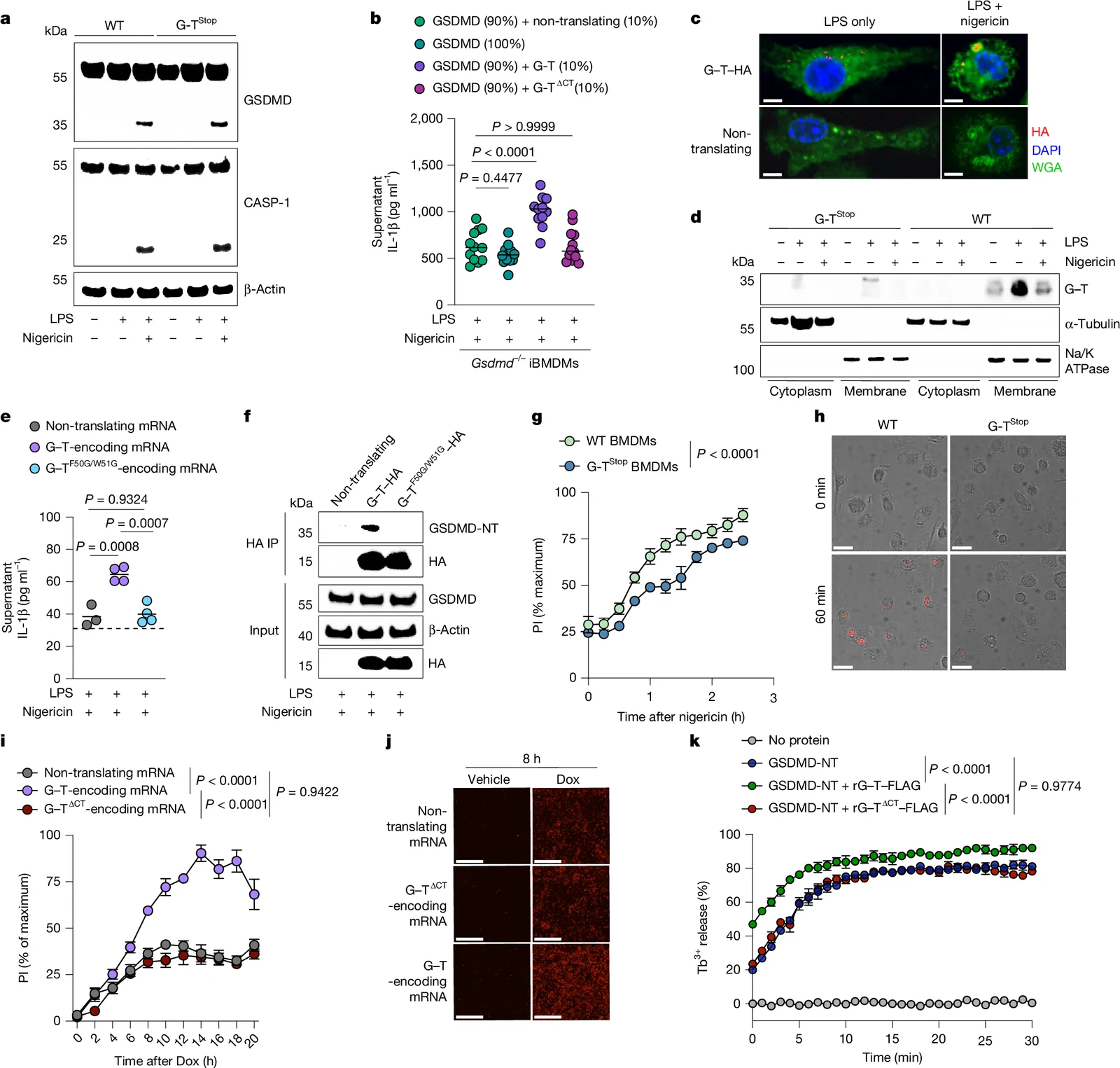
GSDMD–TMEM106A enhances pore formation. **a**, Western blot analysis of full-length and cleaved GSDMD and CASP-1 in wild-type (WT) and G-T^Stop^ BMDM lysates. **b**, ELISA of *Gsdmd*^*−/−*^ iBMDM supernatants after mRNA transfection with the indicated proportions of mRNA and stimulation with LPS and nigericin. The bar represents the mean. *n* = 11 (GSDMD (90%) + G-T (10%)) and *n* = 12 (all other conditions) biologically independent cells. **c**, BMDMs were transfected with mRNA and treated with LPS alone or nigericin. WGA (green), DAPI (blue) and HA-tagged protein (red) are shown. Scale bars, 5 μm. **d**, Western blot analysis of wild-type and G-T^Stop^ BMDM lysates using an anti-GSDMD–TMEM106A antibody in the cytoplasm and membrane cellular fractions. **e**, BMDMs were transfected with mRNA and treated with LPS and nigericin. ELISA of BMDM supernatants was performed at 30 min after treatment with nigericin. The dashed line indicates the limit of detection. Each datapoint represents a biologically independent animal; the bar shows the mean. **f**, BMDMs were transfected with mRNA. Immunoprecipitation was performed for the HA-tag and western blotting was performed. **g**, Wild-type and G-T^Stop^ BMDMs were treated with LPS, nigericin and PI. PI uptake was measured for the indicated time using live imaging.* n* = 4 biologically independent animals per group. **h**, Wild-type and G-T^Stop^ BMDMs were treated with LPS and nigericin and PI. Still-frame images of Supplementary Videos 1 and 2 at 0 and 60 min after nigericin treatment. Scale bars, 25 μm. **i**, PI uptake time course by GSDMD-NT^Dox^ iBMDMs after transfection with mRNA and treatment with PI and doxycycline (Dox). *n* = 4 (non-translating) and *n* = 5 (*Gsdmd-Tmem106a* and *Gsdmd-Tmem106a*^ΔCT^) biologically independent cells. **j**, Representative images of cells from **i** not treated with doxycycline or 8 h after doxycycline treatment. Scale bars, 400 μm. **k**, Liposome leakage was monitored by terbium (Tb^3+^) fluorescence after incubation with the indicated recombinant proteins. *n* = 3 independent experiments per condition. Blots and images are representative of two independent experiments (**a**,** c**,** d**,** f**, **h** and **j**). For **g**, **i** and **k**, data are mean ± s.e.m. *P* values were calculated using one-way ANOVA (**b**, **e**) and two-way ANOVA (**g**, **i** and **k**). Gel source data are provided in Supplementary Fig. 3. Source data

**Extended Data Fig. 8:**
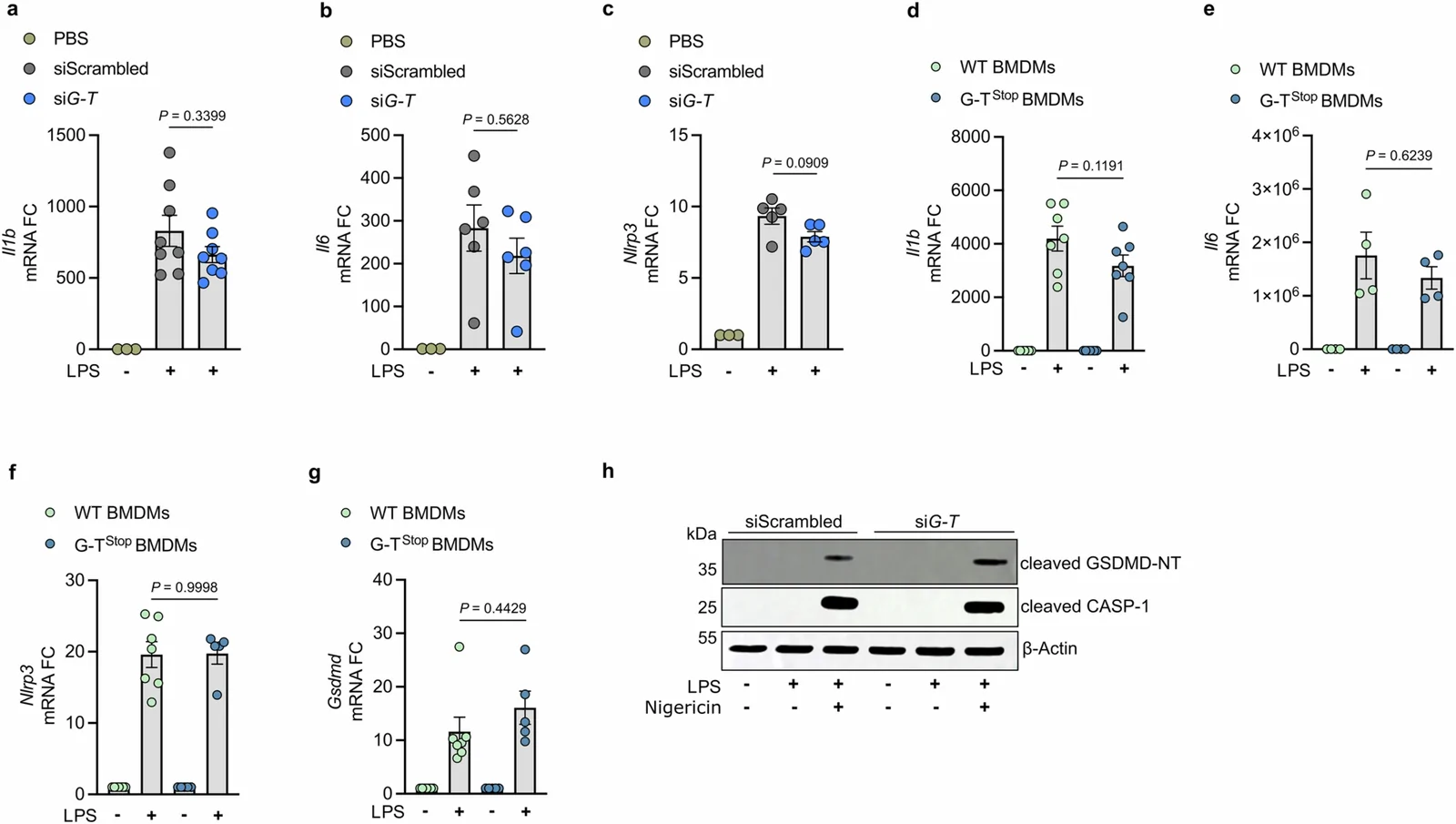
G-T does not alter LPS priming. **a-c**, BMDMs were transfected with siRNA targeting *Gsdmd-Tmem106a* (si*G-T*) or scrambled (siScrambled) siRNA. RT-qPCR; relative gene expression of *Il1b* (**a**), *Il6* (**b**), and *Nlrp3* (**c**) normalized to *Polr2a* fold change (FC) over PBS-treated controls. **d-g**, BMDMs were generated from G-T^Stop^ and wild-type mice and treated with LPS (100 ng/ml; 6 h). RT-qPCR was performed, relative gene expression of (**d**) *Il1b* (n = 7 per group), (**e**) *Il6* (n = 4 per group), (**f**) *Nlrp3* (n = 7 wild-type per group; n = 5 G-T^Stop^ per group), and (**g**) *Gsdmd* (n = 7 wild-type per group; n = 5 G-T^Stop^ per group) normalized to *Polr2a* fold change (FC) over PBS-treated controls. **h**, BMDMs were transfected with siRNA targeting *Gsdmd-Tmem106a* (si*G-T*) or scrambled (siScrambled) siRNA and treated with LPS (100 ng/ml; 6 h) and nigericin (10 μM; 15 min). Western blot of cleaved GSDMD and CASP-1 was performed on cell lysates. β-actin was used as loading control. Each data point represents a biologically independent animal; all bar graphs depict mean ± s.e.m.; one-way ANOVA was used for analysis (**a-g**). For blot source data see Supplementary Fig. 4 (**h**). Source data

To position GSDMD–TMEM106A within the inflammasome pathway, we first examined priming and proteolytic activation. Neither siRNA-mediated depletion nor genetic loss in G-T^Stop^ macrophages altered LPS-induced *Il1b*, *Il6*, *Nlrp3* or *Gsdmd* expression (Fig. 3d and Extended Data Fig. 8a–g). *Gsdmd-Tmem106a* loss also did not affect GSDMD or CASP-1 cleavage after LPS and nigericin treatment (Fig. 5a and Extended Data Fig. 8h), placing its activity downstream of inflammasome assembly and GSDMD cleavage.

As GSDMD–TMEM106A requires canonical GSDMD (Fig. 4l and Extended Data Fig. 7m), we tested whether the proteins function cooperatively. In *Gsdmd*^*−/−*^ iBMDMs expressing a fixed amount of GSDMD, increasing GSDMD–TMEM106A-encoding mRNA dose-dependently enhanced IL-1β release (Extended Data Fig. 7n). Although *Gsdmd-Tmem106a* represents less than 10% of parental *Gsdmd* expression (Extended Data Fig. 7o), supplementing GSDMD-encoding mRNA with 10% GSDMD–TMEM106A-encoding mRNA nearly doubled IL-1β release, whereas an equivalent increase in GSDMD had little effect (Fig. 5b). GSDMD–TMEM106A^ΔCT^-encoding mRNA did not enhance secretion, demonstrating that the TMEM106A-derived C terminus is required for synergy with GSDMD-NT.

**Extended Data Fig. 9:**
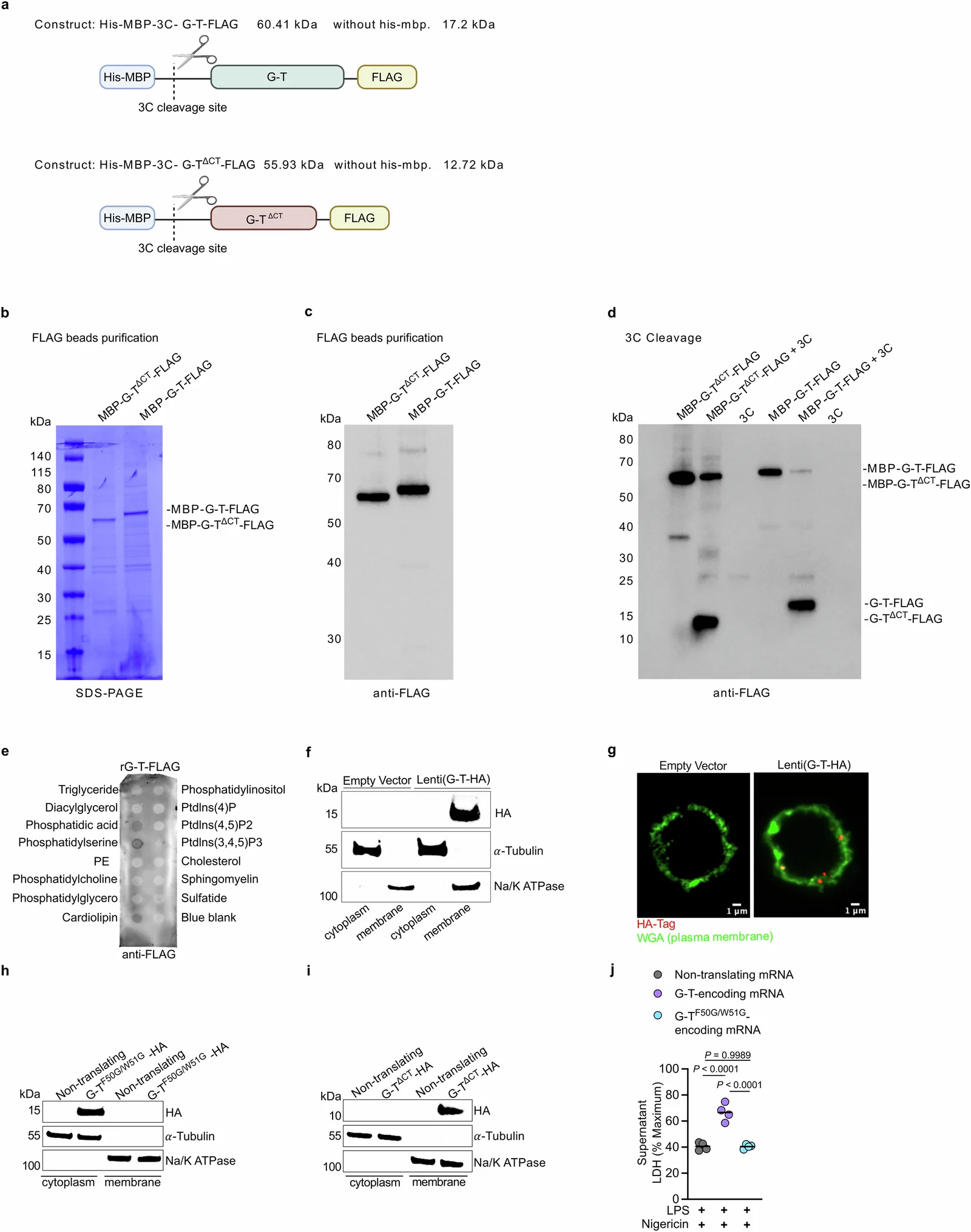
G-T recombinant protein generation and membrane localization. **a**, Schematic of constructs used for generation of recombinant G-T and G-T^ΔCT^ proteins with N-terminal His-MBP and C-terminal FLAG-tags. The diagram was created using BioRender; Venezia, O. https://BioRender.com/z53iqsj (2026). **b**, Coomassie blue staining of SDS-PAGE gel following immunoprecipitation of FLAG protein. **c**, Western blot against FLAG following immunoprecipitation of FLAG protein. **d**, Western blot against FLAG following 3C-protease cleavage of immunoprecipitation of FLAG protein. **e**, Lipids were incubated with GSDMD-TMEM106A recombinant protein with a FLAG-tag (rG-T^FLAG^). Blotting was conducted against FLAG. **f-g**, BMDMs were transduced with lentivirus expressing *Gsdmd-Tmem106a* with a C-terminal HA-tag (Lenti(G-T-HA)) or an empty vector control. (**f**) Western blot against HA in cytoplasm and membrane cellular fractions. Alpha tubulin was used as a cytoplasmic fraction marker and Na/K ATPase was used as a membrane fraction marker. (**g**) Immunofluorescence microscopy z-stack to visualize G-T-HA (red). Plasma membrane (green) was labelled with WGA-AF488. **h-j**, BMDMs were transfected with mRNA encoding a (**h,j**) G-T anchoring residue mutant with a C-terminal HA-tag (G-T^F50G/W51G^-HA) or (**i**) G-T^ΔCT^-HA and control mRNA. Western blot of HA in cytoplasm and membrane cellular fractions. Alpha tubulin was used as a cytoplasmic fraction marker and Na/K ATPase was used as a membrane fraction marker. **j**, Measurement of LDH in BMDM culture supernatant 2 h post-nigericin. Each data point represents a biologically independent animal with mean bar shown. All blots and images representative of two independent experiments. One-way ANOVA (**j**) was used for analysis. For gel source data, see Supplementary Fig. 5. Source data

As GSDMD–TMEM106A lacks the inhibitory GSDMD C terminus, we investigated whether it associates with cellular membranes before inflammasome activation. Recombinant FLAG-tagged GSDMD–TMEM106A (Extended Data Fig. 9a–d) modestly bound to the inner-leaflet lipids phosphatidic acid and phosphatidylserine^36,37^ as well as the mitochondrial lipid cardiolipin^1^ (Extended Data Fig. 9e). Epitope-tagged GSDMD–TMEM106A localized to the plasma membrane without inflammasome activation (Extended Data Fig. 9f,g) and accumulated at membrane protrusions during pyroptosis, when cells become rounded and swollen^38^ (Fig. 5c). Subcellular fractionation further showed that endogenous GSDMD–TMEM106A translocates to membranes during LPS priming (Fig. 5d).

Mutation of the GSDMD-derived membrane-anchor residues Phe50 and Trp51 (ref. ^39^) in GSDMD–TMEM106A-encoding mRNA caused the GSDMD–TMEM106A^F50G/W51G^–HA protein to remain cytoplasmic (Extended Data Fig. 9h). By contrast, the non-functional GSDMD–TMEM106A^ΔCT^–HA protein still localized to membranes, showing that membrane targeting depends solely on the GSDMD-derived region (Extended Data Fig. 9i). Only full-length, membrane-localized GSDMD–TMEM106A enhanced IL-1β release and cell lysis (Fig. 5e and Extended Data Fig. 9j), indicating that membrane localization is necessary for activity.

To test whether membrane-localized GSDMD–TMEM106A interacts with GSDMD-NT, we performed co-immunoprecipitation after NLRP3 activation. Endogenous GSDMD-NT co-precipitated with GSDMD–TMEM106A–HA but not cytoplasmic GSDMD–TMEM106A^F50G/W51G^–HA or the non-translating control (Fig. 5f), demonstrating a physical interaction restricted to the plasma membrane.

**Extended Data Fig. 10:**
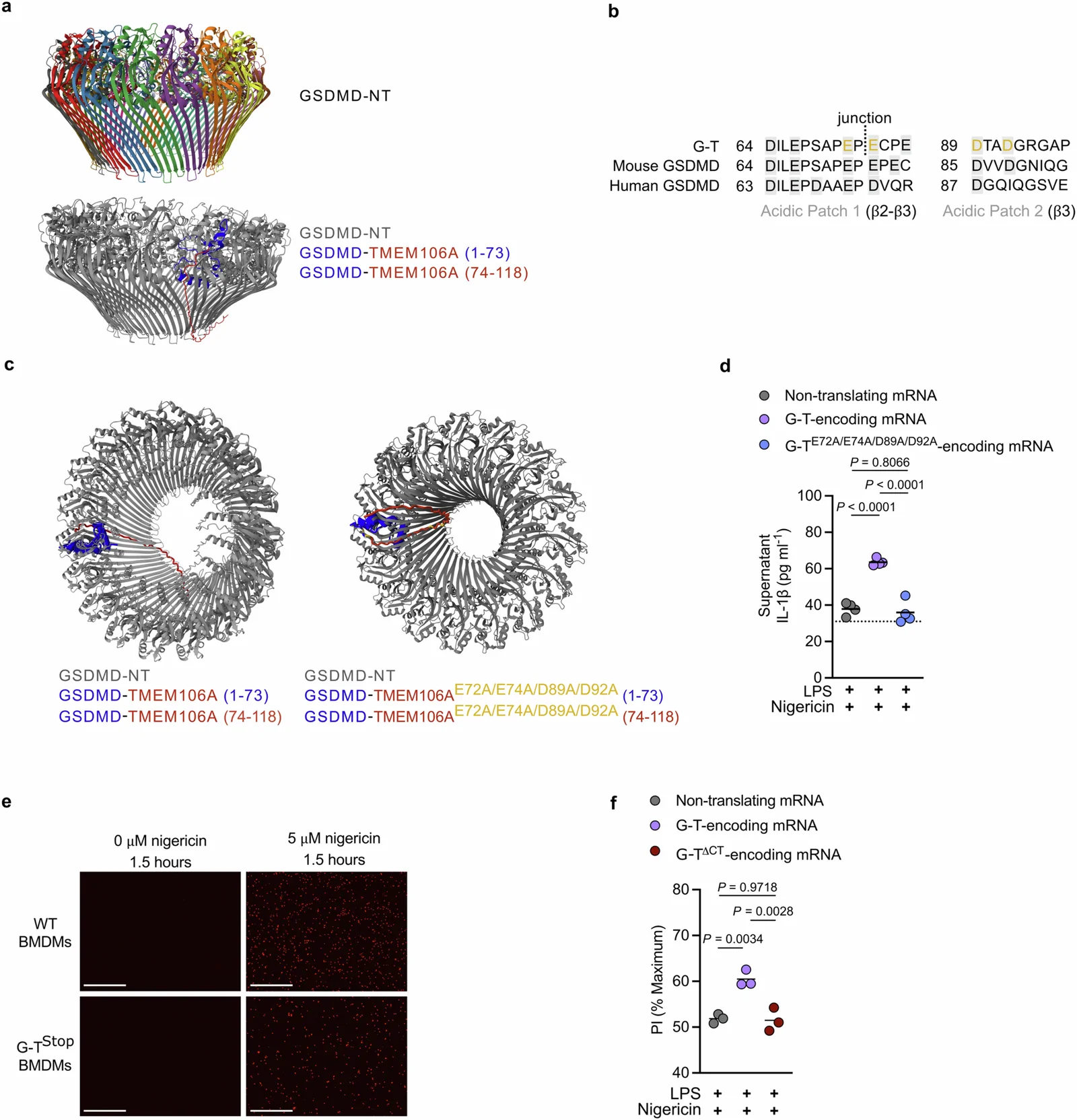
G-T C-terminus is required for enhanced IL-1β release and pore formation. **a**, Schematic depicting folded GSDMD-NT (top) and GSDMD-NT co-folded with G-T (bottom) using AlphaFold 3. **b**, Schematic depicting alignment of acidic amino acids (highlighted in grey) in the region of G-T, mouse GSDMD, and human GSDMD. Mutated acidic residues in G-T^E72A/E74A/D89A/D92A^ in yellow text. **c**, Schematic depicting GSDMD-NT co-folded with G-T (left) and GSDMD-NT co-folded with G-T^E72A/E74A/D89A/D92A^ (right) using AlphaFold 3. Mutated acidic residues in G-T^E72A/E74A/D89A/D92A^ in yellow. **d**, BMDMs were transfected with the indicated mRNA and treated with LPS (100 ng/ml; 6 h) and nigericin (10 μM; 30 min). ELISA was performed on BMDM culture supernatant. Dashed line indicates limit-of-detection for cytokine ELISA assay. **e**, Representative images of PI uptake by wild-type and G-T^Stop^ BMDMs quantified in Fig. 5g. Scale bar depicts 400 μm. Images are representative of two independent experiments. **f**, BMDMs were transfected with the indicated mRNA and treated with LPS (100 ng/ml; 6 h) and nigericin (10 μM) and Propidium iodide (PI) (1:300) for 1 h. PI uptake was measured by plate reader. Each data point represents a biologically independent animal with mean bar shown; one-way ANOVA was used for analysis (**d**,**f**). Source data

AlphaFold 3 predicted co-folding of GSDMD–TMEM106A with the GSDMD-NT pore, positioning its TMEM106A-derived region within the pore (Extended Data Fig. 10a). This region contains four residues conserved in GSDMD-NT acidic patches that are required for IL-1β release^39^ (Extended Data Fig. 10b). Substitution of these residues (E72A, E74A, D89A and D92A) altered predicted co-folding (Extended Data Fig. 10c) and abolished GSDMD–TMEM106A-mediated enhancement of IL-1β release (Extended Data Fig. 10d), establishing their functional importance.

Endogenous GSDMD–TMEM106A loss slowed GSDMD-NT pore formation, as measured by uptake of propidium iodide (PI) (Fig. 5g–h, Extended Data Fig. 10e and Supplementary Videos 1 and 2), whereas overexpression accelerated it (Extended Data Fig. 10f). In cells with doxycycline-inducible GSDMD-NT^40^, *Gsdmd-Tmem106a*, but not *Gsdmd-Tmem106a*^ΔCT^ or control mRNA, increased the rate of PI uptake (Fig. 5i,j), further demonstrating activity downstream of inflammasome activation. Finally, in cell-free liposome-permeabilization assays using purified proteins (Extended Data Fig. 9a–d), recombinant GSDMD–TMEM106A–FLAG, but not recombinant GSDMD–TMEM106A^ΔCT^–FLAG, directly enhanced GSDMD-NT-mediated pore formation (Fig. 5k).

Together, these findings establish GSDMD–TMEM106A as a membrane-localized cofactor that physically and functionally cooperates with GSDMD-NT to accelerate pore formation, amplify early cytokine release and enhance subsequent pyroptosis.

## Discussion

Mammalian cells contain considerable genomic dark matter of functional importance. Over the past several decades, the constricting central tenets of what defines a eukaryotic protein-coding gene have slowly relaxed in lockstep with technological advancement, enabling the identification of novel protein-coding entities. Non-AUG start codons driving translation initiation, evidence of bicistronic translation and small ORF-encoded microproteins have now all been described in mammalian cells in diverse cellular processes ranging from immunity to muscle performance^41–43^. Indeed, in addition to classical linear splicing of exons in a gene, backsplicing events have been identified that create circular mRNA with protein coding potential^44–46^. Here we demonstrate that distinct genes distantly located throughout the genome can create chRNAs during normal physiological responses.

A central finding of this study is that chRNA biogenesis is a rapid, stimulus-responsive process rather than a consequence of genomic instability or splicing noise. Pro-inflammatory signals robustly induce chRNAs in macrophages, with formation occurring at annotated exon boundaries and requiring the canonical spliceosome. For a subset of chRNAs, parent genes are brought into proximity through interchromosomal DNA contacts, which are enhanced within 6 h of inflammation and depend on the genome-organizing protein CTCF^31^. chRNA formation is also highly precise: *Gsdmd* and *Tmem106a*, for example, consistently join at *Gsdmd* exon 2 and *Tmem106a* exon 6. The determinants of this specificity remain unclear but may involve repetitive elements analogous to those involved in circular RNA biogenesis^47^, specialized *trans*-splicing or RNA-binding factors, nuclear condensates, and chromatin or RNA modifications. Notably, tumour-associated fusion transcripts such as *JAZF1-JJAZ1* (ref. ^7^) and *PAX3-FOXO1* (ref. ^9^) can also arise through physiological *trans*-splicing without chromosomal rearrangement. Together with the proposed RNA-before-DNA model^48^ and evidence that three-dimensional genome organization influences translocation-partner selection^49,50^, our findings raise the possibility that chRNAs mark loci that recurrently interact in nuclear space and may become susceptible to translocation during genotoxic DNA repair. Determining whether our chRNA catalogue is enriched for recurrent translocation partners, and directly visualizing how chromatin proximity is coordinated with pre-mRNA joining, will be important future directions.

Our findings support a model in which LPS-induced GSDMD–TMEM106A localizes to the plasma membrane before inflammasome-mediated GSDMD cleavage, creating a prepositioned landing zone that accelerates recruitment and assembly of liberated GSDMD-NT. Consistent with this model, GSDMD–TMEM106A physically interacts with cleaved GSDMD-NT at the membrane, and AlphaFold 3 predicts that its TMEM106A-derived region cooperatively folds with the membrane-inserting region of the GSDMD-NT oligomer. As GSDMD pores are thought to assemble from membrane-associated monomers^40^, this interaction may stabilize only after GSDMD-NT oligomerization, potentially explaining its absence with the cytoplasmic GSDMD–TMEM106A^F50G/W51G^–HA mutant. Analogous to *Drosophila* Myd88 and mammalian PIP2-binding adaptors that concentrate immune effectors at specific membrane domains^51^, binding of GSDMD–TMEM106A to phosphatidic acid and phosphatidylserine may enhance the spatial precision and kinetics of pore formation. Accordingly, its loss attenuates—but does not abolish—cytokine release and pyroptosis, indicating that it amplifies rather than enables canonical pore formation. Its additional binding to cardiolipin raises the possibility that it contributes to GSDMD-mediated mitochondrial damage^52^. Defining its interaction domains, accessory factors and pore architecture will require structural and biophysical approaches, particularly cryo-electron microscopy^39^. Notably, endogenous GSDMD–TMEM106A migrates predominantly above its predicted molecular mass, whereas HA-tagged overexpression produces both higher- and expected-molecular-mass species, suggesting distinct modified, oligomeric and monomeric pools. As the retained GSDMD region contains known sites of phosphorylation^53^, oxidation^40,54^, succination^55^ and ubiquitination^56,57^, defining these biochemical states and their effects on GSDMD–TMEM106A activity represents an important future direction.

Our study reveals a hidden layer of mammalian gene regulation in which spatial genome organization and regulated splicing unite distant genes to generate protein-coding chRNAs. Their potential conservation in mouse and human macrophages suggests that transcript fusion is an underappreciated mechanism for expanding transcriptomic and proteomic diversity. By encoding proteins with novel domain architectures and functions, chRNAs may also broaden the pharmacologically targetable proteome. Their physiological prevalence, including potential fusions involving multiple parent genes and cell type- or context-specific networks, remains largely unclear. Our study provides a generalizable framework for chRNA discovery, validation and manipulation, demonstrating that chRNA-specific interference, mRNA delivery and gene editing can modulate their activity in cells and in vivo. Together, these findings establish chRNAs as a therapeutically actionable layer of mammalian gene regulation that expands the functional proteome and opens a landscape for biological discovery and therapeutic intervention.

## Methods

### Long-read direct RNA-seq

RNA libraries were prepared using the Direct RNA Sequencing kit (SQK-RNA002). Sequencing was performed on the Oxford Nanopore PromethION P24 sequencer using the FLO-PRO002 flow cell. Sequencing was performed using MinKNOW v.22.03.4 and MinKNOW Core v.5.0.0 with a pore scan frequency of 1.5 h, active channel selection set to on and reserved pores set to on. Libraries were run for 72 h. Basecalling was performed using Bream v.7.0.9 and Guppy v.6.0.7. Data processing was performed on an Ubuntu 20.04 system. Sequencing was performed by the BioMicro Center at Massachusetts Institute of Technology.

### Computational analysis of long-read direct RNA-seq data

Individual passing FASTQ files from the same sample were merged to generate a single FASTQ file per sample. Except in the case of JAFFAL^17^ v.2.2 and v.2.3, all mouse data analyses were performed using reference files from GENCODE^58^ release M28 (GRCm39), and all human data analyses were performed using reference files from GENCODE Release 43 (GRCh38.p13). For JAFFAL, matched reference files were downloaded from the UCSC genome browser^59^ and prepared using the instructions provided on the JAFFAL wiki (https://github.com/Oshlack/JAFFA/wiki). Genomic alignments for NanoPlot^60^ v.1.42.0 were performed using Minimap2^61^ v.2.24 with the parameters -ax splice -uf -k14 and aligned reads were processed and analysed using SAMtools^62^ v.1.15. Gene quantification was performed by aligning FASTQ files to the reference transcriptome with Minimap2 using the parameters -ax splice -uf -k14 and then quantifying these alignments with Salmon^63^ v.1.10.2 using the command salmon quant --ont -t <in.fasta> -lU --numBootstraps 30 -a <in.bam> -o <Results>. Salmon data files were imported into R using tximport^64^ v.1.38.2 with counts scaled using the lengthScaledTPM method. Differential gene expression analysis was performed using DESeq2^65^ v.1.50.2 with cooksCutoff=TRUE using the resulting Salmon quant files. log_2_(transcripts per million (TPM) + 1) summary gene quantification data were derived directly from Salmon TPM abundance estimates for genes with expression > 0 in at least one sample.

We created a dedicated pipeline for chimeric RNA analysis called TYPHON, which is available at GitHub (https://github.com/erenada/TYPHON) and reimplements the manually computed chimeric RNA analysis performed in this study using Python modules as part of an integrated, modular workflow. TYPHON provides the ability for users to easily install and run the computational tools and commands used for chimeric RNA analysis in this paper. Please see the TYPHON GitHub page for more details and instructions on running TYPHON.

Chimeric RNA identification using LongGF^16^ v.0.1.2, JAFFAL^17^ and Genion^18^ v.1.2.3. Genomic alignments for LongGF were performed with Minimap2 using the parameters -ax splice -uf -k14–secondary=no -G 50k and processed using SAMtools. LongGF was run on name-sorted .bam files using the command LongGF <bam_file_in> <gtf_file_in> 100 50 100 2 0 1 0 > <Results.log>. JAFFAL was run with MIN_LOW_SPANNING_READS=1 in the make_final_table.R script using the previously generated JAFFAL-specific reference files. As Genion expects reference files to be formatted in the format of Ensembl^66^ reference files, GENCODE reference files were reformatted to be compatible with Genion using a reference script from 10x Genomics^67^ (https://support.10xgenomics.com/single-cell-gene-expression/software/release-notes/build), the command sed ‘s/^chrM/MT/;s/^chrX/X/;s/^chrY/Y/;s/^chr//’ and pygtftk^68^ v.1.6.2 using the command gtftk convert_ensembl -i <input_gtf_file> -o <output_modified_gtf_file>. For running Genion, we patched the tool to be able to output the read IDs for all chimeric RNA detected. This was done by modifying line 1037 of the annotate.cpp file (located in ./genion/src from: “bool full_debug_output = false;” to “bool full_debug_output = true;” and line 1195 from “outfile ≪ fusion_id ≪ “\t” ≪ cand.second.forward.size() ≪ “\t”” to “outfile ≪ fusion_id ≪ “\t” ≪ read_id ≪ “\t” ≪ cand.second.forward.size() ≪ “\t””.). In TYPHON, we enabled full debug output and modified the Genion annotation step to emit one line per supporting read with the Read_ID appended as the final column. Genion was then compiled with this modified annotate.cpp file according to the author’s instructions, and this modified Genion installation was used to run Genion for all chimeric RNA analysis except the analysis of K562 cell data for *BCR-ABL1* benchmarking^19,69^, which was run using the standard Genion installation without any modifications. The selfalign.tsv file required for running Genion was generated by aligning the reference transcriptome fasta file against itself using Minimap2 with the parameters -X -c and the resulting selfalign.paf file was processed to tsv format using pafr v.0.0.2 and tidyverse v.2.0.0. Input .paf files for running each sample through Genion were generated from the same alignment files previously used for running LongGF using the Minimap2 command paftools.js sam2paf <input_sam_file> > <output_paf_file>. Genion was run using the parameters --min-support 1 and with a blank genomicSuperDups.txt file, as an updated version of the genomicSuperDups.txt file for GRCm39 was not available at the time of analysis (see the Genion GitHub page (https://github.com/vpc-ccg/genion) for further details). To keep mouse and human data analyses consistent, a blank genomicSuperDups.txt file was also used for running Genion on human long-read direct RNA-seq data.

For compiling results from LongGF, JAFFAL and Genion, all chimeras in the following results files were considered: the log results files from LongGF, the summary results files from JAFFAL, and the result.tsv files from Genion. For *BCR*-*ABL1* benchmarking with Genion, results.tsv and .fail files were considered. Results from LongGF, JAFFAL and Genion were combined and made distinct by Read ID using tidyverse. Chimeric RNA fasta files were prepared from alignment files using SAMtools and SeqKit^70^ v.2.4.0. Chimeric RNA reads were filtered using BLAST+ (blast)^71^ v.2.13.0 by generating blast reference files using the reference transcriptome and makeblastdb with the parameters -in <reference.fasta.fa> -parse_seqids -blastdb_version 5 -title <reference_title> -dbtype nucl -out <db_name> command, and then running blastn with the parameters -query <fasta_containing_chimeric_RNA_sequences> -db <db_name> -outfmt 6 -out <output_results.txt> on a single fasta file containing chimeric RNA reads. Processing of blast results and generation of exon-repaired chimeric RNA reads was performed using SeqKit, BEDOPS^72^ v.2.4.41, BEDTools^73^ v.2.31.0, Clustalo^74^ v.1.2.4, Clustalw^75^ v.2.1, Biopython^76^ v.1.81, Seqtk v.1.4, tidyverse and data.table v.1.15.2. In brief, chimeric RNAs that did not map to at least one isoform of each chimeric RNA parent gene were removed. Mapping to isoforms containing retained introns was disallowed. BLAST mappings were ranked by descending bit.score and the BLAST mapping with the highest bit.score was selected for each chimeric RNA parent gene. Blast mappings for each chimeric RNA were ordered according to BLAST mapping order in the original chimeric RNA read. The genomic coordinates for every exon present in each selected chimeric RNA isoform were imported from the reference GTF file using BEDTools and the breakpoint exons for each chimeric RNA transcript were estimated by calculating cumulative exon length on a per-transcript basis and comparing against the length of each chimeric RNA segment provided by BLAST. This method enabled the calculation of which exons were expected to be present in each repaired chimeric RNA transcript. Selected exon coordinates were then ordered and added to bed files. Corresponding exonic sequences were extracted from the reference genome and fasta files containing the exon-repaired reads for each chimeric RNA were generated.

Conservation analysis between chimeric RNA present in mice and humans was performed by conducting predicted chimeric protein analysis, constructing BLAST and BLASTp references using human chimeric RNA sequences and predicted chimeric protein sequences, and then running BLAST or BLASTp on mouse chimeric RNA transcripts and predicted chimeric protein sequences against the computed human chimeric RNA and chimeric protein databases. Chimeric RNA peptide prediction was performed using orfipy^77^ v.0.0.4 using the parameters <chimeric_RNA_sequence_fasta.fa> --min 90 --max 1000000000 --procs 1 --strand f --start ATG --stop TAA,TAG,TGA --table 1 --outdir <out_directory> --pep <Predicted_peptide_fasta.fa>. The BLASTp reference construction command was makeblastdb with the parameters -in <reference_proteome.fasta> -parse_seqids -blastdb_version 5 -title <reference_title> -dbtype prot -out <db_name>. Nichenetr^78^ v.2.2.0 was used to convert human to mouse gene symbols using the convert_human_to_mouse_symbols R function, and mouse and human transcript BLAST matches were required to be derived from parent gene orthologues in both species. BLASTp was run using the parameters -query <in_fasta> -db <db_name> -outfmt 6 -out <blast_result.txt>. The resulting BLAST and BLASTp results were ordered by decreasing bit.score (high to low) and alignment length (longer to shorter) and the top hit for each mouse chimeric RNA Read ID was selected. dplyr v.1.1.4, stringr v.1.5.1, data.table and rtracklayer^79^ v.1.64.0 were used for processing of BLAST data. For applications downstream of exon repair, long-read RNA-seq-derived chimeric RNA chimera IDs were corrected to appear in biological order as determined using BLAST+ and exon repair, with coordinates and other information also reordered appropriately.

### Short-read RNA-seq

RNA libraries were prepared using the KAPA mRNA HyperPrep Kit (Roche). Total-RNA samples were quantified using the Agilent 4200 TapeStation instrument, with the corresponding Agilent TapeStation RNA assay. The resulting RNA-integrity number (RIN) scores and concentrations were considered when qualifying samples to proceed. An average RIN score of >9 was recorded for all samples. Samples were normalized to 200 ng of input in 50 μl (4 ng μl^−1^), and the mRNA was captured using oligo-dT beads as part of the KAPA mRNA HyperPrep workflow. cDNA synthesis, adapter ligation and amplification were conducted subsequently as part of the same workflow. After amplification, residual primers were eluted away using KAPA Pure Beads in a 0.63× SPRI-based cleanup. The resulting purified libraries were run on the Agilent 4200 Tapestation instrument, with the corresponding Agilent High Sensitivity D1000 ScreenTape assay to visualize the libraries and check that the size and concentrations of the libraries matched the expected product. qPCR using the KAPA Library Quantification kit, which uses primers complementary to the sequencing flowcell oligos, was run to confirm the functional concentration. Molarity values obtained from this assay were used to normalize all samples in equimolar ratio for one final pool. The pool was denatured and loaded onto the Illumina NovaSeq6000 instrument, with an S4 300-cycle kit to obtain paired-end 150 bp reads. The pool was loaded at 1.2 pM, with 5% PhiX spiked in as a sequencing control. Basecall files were demultiplexed through the Harvard BPF Genomics Core’s pipeline, and the resulting FASTQ files were used in subsequent analysis.

### Computational analysis of short-read RNA-seq data

FASTQ files were trimmed using fastp v.0.23.2 with the default parameters and trimmed FASTQ files were used as input for short-read chimeric RNA detection unless otherwise specified. GENCODE M28 reference files were used unless otherwise specified. Chimeric RNA were detected in short-read RNA-seq data using *n* = 6 different computational tools, Arriba, FusionCatcher, STAR-Fusion, STAR-SEQR, JAFFA and Pizzly^20–24^. Arriba v.2.4.0 was run by first running star v.2.7.11b with the parameters --outSAMattributes Standard --outSAMtype BAM Unsorted --outSAMunmapped Within --outFilterMultimapNmax 50 --peOverlapNbasesMin 10 --alignSplicedMateMapLminOverLmate 0.5 --alignSJstitchMismatchNmax 5 -1 5 5 --chimSegmentMin 10 --chimOutType WithinBAM HardClip --chimJunctionOverhangMin 10 --chimScoreDropMax 30 --chimScoreJunctionNonGTAG 0 --chimScoreSeparation 1 --chimSegmentReadGapMax 3 --chimMultimapNmax 50, and then running Arriba with the default settings and a blank blacklist.txt file. Reference files for running Arriba were generated using STAR --runMode genomeGenerate --genomeFastaFiles <input_fasta> --sjdbOverhang 150. Results from Arriba were processed to remove ambiguous intergenic chimeric RNA reads. The reference for FusionCatcher v.1.33 was built on 13 March 2025 using Ensembl 113 mouse reference files using fusioncatcher-build -g mus_musculus. FusionCatcher was run on untrimmed FASTQ files using the default settings other than --limitSjdbInsertNsj 3000000 --limitOutSJcollapsed 3000000. STAR-Fusion references were generated by downloading the Mouse_GRCm39_M31_CTAT_lib_Nov092022.source.tar.gz archive from https://data.broadinstitute.org/Trinity/CTAT_RESOURCE_LIB/ and following the ‘Building a Custom Genome Resource Library for Fusion Detection’ instructions from https://github.com/TrinityCTAT/ctat-genome-lib-builder/wiki to build the reference with GENCODE M28 reference files. STAR-Fusion v.1.12.0 was run with the parameters --no_annotation_filter --min_FFPM 0 --min_sum_frags 1.

STAR-SEQR was run using STAR-SEQR v.0.6.7 by first generating reference files using STAR --runMode genomeGenerate --genomeFastaFiles --sjdbOverhang 150 --genomeSAsparseD 1 with GENCODE M28 reference files. Individual FASTQ from different lanes for the same sample were concatenated into single R1 and R2 files and the parameters -m 1 -vv --keep_mito were used. JAFFA v.2.3 was run using the same reference files as used for long-read RNA-seq analysis on sample-level concatenated single R1 and R2 FASTQ files using the parameters <JAFFA-version-2.3/tools/bin/bpipe> run <JAFFA-version-2.3/JAFFA_direct.groovy>. pizzly v.0.37.3 was run on untrimmed fastq files by first running kallisto^80^ v.0.48.0. The kallisto reference was generated by modifying the GENCODE M28 transcriptome reference using zcat </path/transcripts.fa.gz> | tr '|' ' ' | gzip -1> </path/transcripts.fixed.fa.gz> and building the reference using kallisto index -i </path/index.idx> </path/transcripts.fixed.fa.gz>. Kallisto was then run using kallisto quant with the --fusion parameter and pizzly was run using pizzly with the parameters -k 31 --align-score 2.

For calculation of junction-level overlap between chimeric RNA from long-read and short-read RNA-seq data, coordinates provided by Arriba, FusionCatcher, STAR-Fusion, STAR-SEQR and JAFFA were used and compared against long-read coordinates derived from exon-repair (that is, breakpoint exon coordinates). For FusionCatcher, coordinates were first translated from Ensembl 113 to GENCODE M28 coordinates by mapping the FusionCatcher-provided gene symbol to the corresponding GENCODE M28 gene symbol using Mouse Genome Informatics (MGI) (https://www.informatics.jax.org/) as an intermediate with the scCustomize^81^ v.3.2.0 Updated_MGI_Symbols function. Then, using exon breakpoints, the closest (in terms of absolute nucleotide distance) matching exon in the Ensembl reference was calculated against GENCODE coordinates to determine GENCODE M28 coordinates for each chimeric RNA found using FusionCatcher. These inferred GENCODE M28 coordinates were then used for junction-level overlap calculation. For Pizzly, which provides transcriptomic information, a strategy similar to that used for exon-repair was used to infer coordinate breakpoints for junction-level overlap. The transcript breakpoints in the annotated Pizzly chimeric RNA parent transcripts were compared against matched cumulative exon length metrics for GENCODE M28 transcripts and breakpoint exons were inferred based on minimum absolute nucleotide distance. An absolute nucleotide distance of ≤10 nucleotides between the short-read RNA-seq chimeric RNA gene A and gene B breakpoints and the long-read gene A and gene B breakpoints was required for a chimeric RNA to be considered to have the same breakpoint junction. For breakpoint junction calculation, long-read RNA-seq-derived chimeric RNA chimera IDs were corrected to appear in biological order as determined using BLAST+ and exon repair, with coordinates and other information also reordered appropriately. The chimera IDs from chimeric RNA derived from short-read RNA-seq were not reordered, as these chimeric RNA were not processed with BLAST/exon-repair.

FFPM-like metrics for each short-read RNA-seq tool were calculated using the broad strategy of FFPM_metric = (split_reads1 + split_reads2 + discordant_mates)/(total_mapped_reads/1,000,000) on a per tool basis (as non-identical methodologies and outputs are used/provided by each chimeric RNA detection tool). Notably, the detection of FFPM-like metrics for fusion genes, as well as any downstream differential analysis, is difficult and estimative by nature, and was used in this study for the purpose of selecting potentially interesting downstream candidates in tandem with long-read RNA-seq co-identification; metrics derived using this methodology should be considered an estimate and should not be interpreted in the same manner as standard gene expression and differential analysis metrics. For Arriba, the sum of split_reads1 + split_reads2 + discordant_mates was calculated on a chimera ID level with total mapped reads being derived from STAR-generated bam files using samtools view -@ 30 -c -F 1028. For FusionCatcher, Spanning_unique_reads was used in place of (split_reads1 + split_reads2 + discordant_mates) and the number for total mapped reads was derived from the ‘# reads with at least one reported alignment’ in the info.txt files provided by FusionCatcher. Metrics for STAR-Fusion were calculated using JunctionReadCount + SpanningFragCount in place of (split_reads1 + split_reads2 + discordant_mates) using the same total mapped reads counts as used for Arriba. For STAR-SEQR, the total mapped reads counts were extracted from the STAR-SEQR Log.final.out log files as the ‘Uniquely mapped reads number’ number, and metrics were calculated using NREAD_SPANS + NREAD_JXNLEFT + NREAD_JXNRIGHT in place of (split_reads1 + split_reads2 + discordant_mates). For Pizzly, the total mapped reads counts were extracted from the run_info.json files n_processed numbers provided by Pizzly, and metrics were calculated using total_paircount + total_splitcount instead of (split_reads1 + split_reads2 + discordant_mates). Finally, for JAFFA, total mapped reads counts were calculated using the result of (seqkit stats -T -j 4 <fastq_file> | tail -n +2 | cut -f4)*2 with the <sample>_filtered_reads.fastq.1.gz file for each sample as the input, with spanning.pairs + spanning.reads used in place of (split_reads1 + split_reads2 + discordant_mates). All FFPM-like metric results were filtered to only apply to short-read chimeric RNA with breakpoint junctions overlapping long-read RNA-seq-derived chimeric RNA breakpoint junctions. FFPM calculations were performed by taking individually derived FFPM-like metrics for each tool and calculating log_ffpm <- log_2_(total_filter + 0.01). Differential testing was performed using limma^82^. Calculation of relative enrichment between interchromosomal and distal/proximal intrachromosomal chRNA was performed using the summed counts originally derived for ffpm-like metrics. Counts were combined for individual read IDs corresponding to the same chRNA ID or for the same chRNA ID and junction in the case of density plots. Significance testing was performed using the R t.test function with the default settings.

### NanoString nCounter analysis

NanoString nCounter analysis was conducted on purified, extracted RNA by the Genetics and Genomics Core at Boston Children’s Hospital in accordance with standard NanoString protocols using a NanoString nCounter Max System. Analysis of NanoString RCC files from BMDMs was performed using the NanoTube^83^ v.1.6.0 package using nSolver normalization, a background proportion of 0.33 and a background threshold of 2. The normalized count matrix of passing chimeric RNA expression data was converted to log_2_ format using the formula log_2_(*x* + 0.5), where *x* is defined as the normalized count matrix. For BMDM data, chimeric RNAs that were expressed below the background, defined as a value of −1 in the log_2_ normalized matrix, in less than 75% of replicates of any one polarization condition were removed from the analysis. Differential expression analysis was performed using the runLimmaAnalysis function. For analysis of *Gsdmd-Tmem106a* expression in vivo, the nSolver analytical pipeline was followed using standard settings using nSolver version ≤4.0.

### Hi-C processing

The Hi-C libraries were processed as previously described with changes in parameters^84^. Libraries were aligned with the presplit_map.py script in the diffHic package (v.1.38.0)^85^. Reads were split into 5′ and 3′ segments if they contained the MboI ligation signature (GATCGATC), in cutadapt (v.4.8)^86^ with the default parameters. Reads were aligned to the mm39 build of the mouse genome with bowtie2 (v.2.5.3)^87^ in single-end mode. The FixMateInformation command from the Picard suite v.1.117 (https://broadinstitute.github.io/picard/) was applied to synchronize mate information for each read pair. Duplicates were marked with MarkDuplicates from Picard and sorted by name with SAMtools. Each BAM file was processed to identify alignments for each read to a specific MboI restriction fragment with the preparePairs function in diffHic. Duplicate reads and those with mapping quality scores below 10 were discarded. Read pairs were determined to be dangling ends and removed if the pairs of inward-facing reads or outward-facing reads on the same chromosome were separated by less than 1,000 bp for inward-facing and outward-facing reads. Read pairs with fragment sizes above 1,000 bp were removed. Biological replicates were summed with the mergePairs function. HDF files of read pairs were converted to the Juicer format .hic with juicer tools (v.1.22.01)^88^.

### APA

Aggregate peak analysis (APA) was performed using the R package GENOVA^89^ v.1.0.1. Files were loaded using the load_contacts function with resolution 500 kb and balancing equal TRUE. A total of 1,047 interchromosomal chRNA fusions found in BMDMs and co-detected by LongGF, JAFFAL and Genion were used for APA. Two controls were created for comparisons. For randomized chRNA fusion partners, 10,000 random combinations of the interchromosomal fusion regions were created, intrachromosomal combinations were removed. For random exons, 10,000 exon combinations were created by sampling from all exons in the genome; the inbuilt mm39 annotation from Rsubread^90^ version ≤2.26.0 was used to define exons and intrachromosomal combinations were removed. APA plots were created using the APA function with size_bin=11 and dis_thres = c(500000, Inf) and all plotted on the same scale. APA was quantified with the quantify function and the fold-change value used for each interaction (the ratio of the foreground and background). Fold-change values in cases in which the background was 0 were removed for all samples. *P* values were calculated using a paired *t*-test of the log_2_(0.5 + fold change) values.

### Mice

All mouse experiments were carried out in compliance with Harvard Medical School Animal Care and Use Committee protocols. For most experiments unless otherwise specified in the text, experiments were conducted using male and female, 8–12-week-old C57BL/6J (000664) mice from Jackson Laboratory in an unblinded manner. BALB/cJ (000651) and wild-derived WSB/EiJ (001145) mice were also procured from Jackson Labs. *Gsdmd-*knockout mice were gifted by I. Chiu.

To generate G-T^MYC^ mice, a ssDNA donor oligonucleotide containing a flexible linker sequence followed by a MYC epitope tag sequence flanked with homology arms was provided to facilitate homology-directed repair insertion of the sequence into the C terminus of the *Gsdmd-Tmem106a* open reading frame. In brief, a guide RNA (GAGGGACGTTACCTGCTCAC) and ssDNA Ultramer (IDT) donor template (GGCCCATTGGCCAG**GGTGGTTCTGGTGGTGGTTCTGGTGGTGGTTCTGGTGAACAAAAACTCATCTCAGAAGAGGATCTG**TGAGCAGGTAACGTCCCTCTTCCTGGCCAGCCCTCTACACAGGCATGTAATGTGGTGACGGAGAGGGGCCAGCCTGAGGT) (bold font represents the knock-in cassette; non-bold represents homology arms) were designed targeting the stop codon locus of *Gsdmd-Tmem106a* in exon 6 of *Tmem106a*. The sequence of the knock-in mouse was confirmed using Sanger sequencing of the genomic loci. The forward primer (TTTCTGGTGCATCTGAAGACA) and reverse primer (GAGACCAAAGGGCACTCACT) were designed to amplify the mutation site. Knock-in alleles were identified as 496 bp PCR products and wild-type alleles were identified as 430 bp PCR products by gel electrophoresis.

To generate G-T^Stop^ mice, a ssDNA donor oligonucleotide containing a point mutation with homology arms was provided to facilitate homology-directed repair insertion of the sequence into exon 6 of *Tmem106a*. Two guide RNAs (AGTGACACAGCTGACGGCCG; AGATGTTCAGGACATTCTGG) and ssDNA Ultramer (IDT) donor template (TCTTTTTTAAAAAAGGTCTGAAAAAAATTTTAAATTATGCTTTTCCTTGCTGTCAACTCCAGAATGTCCTTAACATCTTCAACAGCAACTTCTATCCCATCACAGTGACACAGCTGACGGCCGAAGTGCTCCACCAGGCCTCT) were designed targeting exon 6 of *Tmem106a*. The sequence of knock-in mice was confirmed using Sanger sequencing of the genomic loci. The forward primer (ACACCCTCTTTCTGGTGCAT) and reverse primer (TACCTCAGCAGCCCTTCAGT) were designed to amplify the mutation site.

### Mouse BMDM generation and polarization

BMDMs were generated from progenitor cells isolated from the femurs and tibias of C57BL/6J (obtained from the Jackson Laboratory), G-T^Stop^ or G-T^MYC^ mice and maintained in macrophage-colony stimulating factor (M-CSF) (50 ng ml^−1^) for 7 days. Medium containing M-CSF was refreshed after 4 days. To induce an inflammatory phenotype, cells were stimulated with LPS (100 ng ml^−1^) (serotype O55:B5; Enzo, ALX-581-013-L001) and recombinant mouse IFNγ (20 ng ml^−1^) (PeproTech, 315-05) for 24 h. To induce a tissue reparative phenotype, cells were stimulated with recombinant mouse IL-4 (20 ng ml^−1^) (PeproTech, 214-14) and mouse recombinant IL-13 (20 ng ml^−1^) (PeproTech, 210-13) for 24 h.

### Human MDM generation and polarization

MDMs were generated from CD14^+^ cells. For Oxford Nanopore Sequencing, anonymous peripheral blood samples were collected from the Mississippi Valley Regional Blood Center as waste cellular products and CD14^+^ cells were isolated using the MojoSort Human CD14 Selection Kit (BioLegend, 480026). For PCR and Sanger sequencing, anonymous cryopreserved CD14^+^ monocytes were purchased from Lonza (2W-400C). All monocytes were stimulated with M-CSF (50 ng ml^−1^) (BioLegend, 574806) and granulocyte-macrophage-colony stimulating factor (GM-CSF) (50 ng ml^−1^) (BioLegend, 572904) in RPMI 1640 containing 10% FBS for 7 days. To induce an inflammatory phenotype, cells were stimulated with LPS (100 ng ml^−1^) (serotype O55:B5; Enzo, ALX-581-013-L001) and recombinant IFNγ (20 ng ml^−1^) (BioLegend, 570204) for 24 h.

### DNA and RNA extraction, Sanger sequencing and PCR

RNA and DNA were extracted from BMDMs using Trizol according to the manufacturer’s protocol. cDNA synthesis for PCR and RT–qPCR was performed using Maxima Reverse Transcriptase (Thermo Fisher Scientific, EP0741). PCR and RT–qPCR for chRNA were performed using a forward primer in gene A and a reverse primer in gene B. The primers used for PCR and Sanger sequencing of chRNAs that were co-identified by all three long-read RNA-seq tools and have a parent gene with an annotated function in immunity are provided in Supplementary Table 9. PCR and RT–qPCR for *CD44-FAM168B* was performed using a forward primer against *CD44* (CCCATCCCAGACGAAGACAG) and a reverse primer against *FAM168B* (GTACACGGCAGTCTGGTAGG). PCR and RT–qPCR for *ITGAX-CD52* was performed using a forward primer against *ITGAX* (GGGATGCCGCCAAAATTCTC) and a reverse primer against *CD52* (GCTGAGACGTGTCACCTCAA). PCR and Sanger sequencing for *HDAC8-CITED1* in human cDNA and *Hdac8-Cited1* in mouse cDNA were performed using a forward primer in *HDAC8* (human: TTATGACTGCCCAGCCACTG; mouse: TTGCGACGGAAATTTGACCG) and a reverse primer in *CITED1* (human: TCCCGAGGAACTAGTGGGAG; mouse: TGCCTTGCGATCCTTCACTC). PCR and Sanger sequencing for *TBC1D22B-RNF8* in human cDNA and *Tbc1d22b-Rnf8* in mouse cDNA were performed using a forward primer in *TBC1D22B* (human: CGTCAACTTCTCTCCAGCCA; mouse: TGACATTCCGAGGACGAACC) and a reverse primer in *RNF8* (human: TCCGACAAATGGGGCATTCT; mouse: CCTTCACGTCTGAGCTTAGGTC). PCR was performed using a ProFlex 3×32-well PCR system from Applied Biosystems. For long-range genomic PCRs, Phusion high fidelity DNA polymerase was used (New England Biolabs, M0530S) and the extension time was increased to amplify products up to 10 kb. Sanger sequencing was performed on a 1% agarose-gel-purified PCR product using a forward primer. Gel purification was conducted using the Monarch Spin DNA Gel Extraction kit (New England Biolabs, T1120L). RT–qPCR was performed using Sybr Green (Thermo Fisher Scientific, S7567) on the QuantStudio 5 Real-Time PCR System and all gene expression was assayed in duplicate.

### LPS injection for NanoString peritoneal macrophage isolation

C57BL/6J mice (male, aged 8 weeks) from Jackson Laboratory were treated intraperitoneally with 100 μl PBS or LPS (*E. coli* strain O111:B4; InvivoGen, tlrl-3pelps) in PBS (5 mg kg^−1^) for 16 h. Peritoneal lavage was collected in PBS containing 3% FBS and F4/80^+^ cells were isolated using MACS magnetic bead enrichment with anti-F4/80 MicroBeads (Miltenyi Biotec, 130-110-443) according to the manufacturer’s protocol. F4/80^+^ cells from three mice within the same treatment group were pooled in Trizol and RNA was extracted for NanoString nCounter analysis.

### Intracisternal infection and brain macrophage isolation

C57BL/6J mice (male, aged 8 weeks) from Jackson Laboratory were subjected to intracisternal injection of 5 μl PBS or 1.2 × 10^3^ CFU *E. coli* (strain C5; ATCC, 700973) in 5 μl PBS. Then, 18 h after treatment, the mice were euthanized and perfused for collection and homogenization of the hindbrain. Isolated cells were resuspended in HBSS and an enrichment for myeloid cells was performed by adding 90% Percoll and centrifuging at 500*g* for 60 min at 4 °C. CD11b^+^ cells were isolated using MACS magnetic bead enrichment with anti-CD11b MicroBeads (Miltenyi Biotec, 130-126-725) according to the manufacturer’s protocol. Cells were collected in Trizol and RNA was extracted for NanoString nCounter analysis.

### Intranasal infection and pulmonary macrophage isolation

C57BL/6J mice (female, aged 8 weeks) from Jackson Laboratory were infected intranasally with 30 μl PBS or influenza A (strain PR8) for 7 days. In brief, after infection, mice were euthanized and the perfused lung was collected. Lung tissue was digested in 2 mg ml^−1^ collagenase IV solution in PBS with DNase I (100 μg ml^−1^) at 37 °C shaking for 30 min. Homogenized and filtered lung tissue was subjected to a 33%/66% Percoll gradient and centrifuged at 3,000 rpm for 30 min at room temperature. Immune cells in the interface were isolated for FACS. Live CD45^+^F4/80^+^CD64^+^ single cells were sorted and stored in Trizol for RNA extraction.

### Pladienolide B splice inhibitor treatment

BMDMs were plated 1.5 × 10^6^ cells per well in a 6-well plate and co-treated with 100 ng ml^−1^ LPS (Serotype O55:B5; Enzo, ALX-581-013-L001) and 100 nM pladienolide B (PlaB) (Santa Cruz Biotechnology, sc-391691) or 0.1% DMSO (Sigma-Aldrich, D2438) for 6 h. Cells were collected in 1 ml of Trizol per well for RNA extraction.

### Actinomycin D transcription inhibitor treatment

BMDMs were plated 1.5 × 10^6^ cells per well in a 6-well plate and co-treated with 100 ng ml^−1^ LPS (Serotype O55:B5; Enzo, ALX-581-013-L001) and 5 μg ml^−1^ actinomycin D (Gibco, 11805017) or 0.1% DMSO (Sigma-Aldrich, D2438) for 3 h. Cells were collected in 1 ml of Trizol per well for RNA extraction.

### siRNA transfection for knockdown

Two siRNA were designed and pooled to target the junction sequence of *Gsdmd-Tmem106a* (CAGAACCAGAAUGUCCUGAACAU; AGAACCAGAAUGUCCUGAACA). Scrambled target sequences were generated for scrambled control siRNA design (AUAGCGAUACCAACAUAUCGACG; GAUUAACACGAACUAGCGAAC). siRNAs targeting *Ctcf* (CGAUCAGUUUCUGUCCUGAAAG), *Gsdmd* (GUGGUCAAGAAUGUGAUCAAGG) and *Rnu1a1* (GGAGAUACCAUGAUCACGAAGGUGG) were additionally designed and synthesized by IDT. Negative control siRNA (siCtrl) was purchased from IDT (51-01-14-03). BMDMs were plated at a density of 2 × 10^5^ cells per well in a 24-well-plate for cytokine secretion and LDH assays, and 1 × 10^6^ cells per well in a 6-well-plate for all RT–qPCR and western blot analyses. Once cells adhered (over 12 h after plating), siRNA was transfected in Lipofectamine RNAiMAX (Thermo Fisher Scientific, 13778100) according to the manufacturer’s protocol. Assays examining the effects of si*G-T* and si*Gsdmd* were performed 48 h after transfection. Assays examining the effects of si*Ctcf* and si*Rnu1a1* were conducted 24 h after transfection.

### Endogenous GSDMD–TMEM106A western blots

BMDMs were generated from homozygous G-T^MYC^ and wild-type littermate control mice. Cells were plated at 8 × 10^6^ and left untreated or stimulated with LPS (100 ng ml^−1^) (serotype O55:B5; Enzo, ALX-581-013-L001) for 6 or 24 h. After stimulation, whole-cell lysates were collected using NP40 lysis buffer containing 10% PMSF and 1× protease inhibitor and subjected to western blot using anti-MYC antibody (9B11) (Cell Signaling Technology, 2276S) (1:1,000) and anti-mouse HRP-linked secondary antibody (Cell Signaling Technology, 7076S) (1:3,000). Anti-β-Actin antibody (13E5) (Cell Signaling Technology, 4970, 1:1,000) was used as the loading control for whole-cell lysates.

Production and purification of a custom antibody against GSDMD–TMEM106A were performed by Thermo Fisher Scientific. In brief, rabbits were immunized with peptide antigen CGRPGHQQPPPAHRPIGQ, including an initial injection and three booster injections over the course of 56 days. Antibody was affinity purified from serum and titred by indirect ELISA against our target peptide. For detection of endogenous GSDMD–TMEM106A in wild-type and G-T^Stop^ mice, BMDMs were left untreated or were treated with LPS (100 ng ml^−1^) (serotype O55:B5; Enzo, ALX-581-013-L001) for 6 h. Whole-cell lysates were collected using NP40 lysis buffer containing 10% PMSF and 1× protease inhibitor and processed for western blotting using anti-GSDMD–TMEM106A custom antibody (1:500) and anti-rabbit HRP-linked secondary antibody (Cell Signaling Technology, 7074S, 1:3,000). For detection of endogenous GSDMD–TMEM106A in different cellular fractions of wild-type and G-T^Stop^ mice, 5 × 10^6^ BMDMs were collected after no stimulation, after 6 hr LPS (10 ng ml^−1^) treatment alone, or after LPS prime and nigericin treatment (10 μM) for 15 min. Subcellular fractionation was conducted using the Thermo Fisher Scientific Subcellular Protein Fractionation Kit for Cultured Cells (Thermo Fisher Scientific, 78840) according to the manufacturer’s instructions. Western blotting was performed using anti-GSDMD–TMEM106A custom antibody (1:500) and anti-rabbit HRP-linked secondary antibody (Cell Signaling Technology, 7074S, 1:3,000). Anti-α-tubulin antibody (DM1A, Cell Signaling Technology, 3873, 1:1,000) was used as subcellular fractionation control for cytoplasmic protein isolation. Anti-Na,K-ATPase antibody (Cell Signaling Technology, 3010, 1:1,000) was used as subcellular fractionation control for membrane protein isolation.

All western blots were conducted using 4–12% NuPAGE Bis-Tris gels (Thermo Fisher Scientific, NP0321BOX) according to the manufacturer’s protocol for the XCell SureLock Mini-Cell system and transferred using the XCell II Blot Module (Thermo Fisher Scientific, EI9051).

### mRNA transfection for overexpression

Codon optimized mRNA expressing the amino acid sequence of interest was designed and synthesized by Moderna. *Gsdmd-Tmem106a* mRNA was designed to encode the wild-type GSDMD–TMEM106A sequence, while *Gsdmd*-*Tmem106a*^ΔCT^ mRNA encodes the same truncated sequence expressed by G-T^Stop^ mice, whereby a stop codon is placed just three amino acids into the *Tmem106a*-encoded C terminus. mRNA was transfected into BMDMs using MessengerMax lipofectamine (Thermo Fisher Scientific, LMRNA001) for 24 h before the assay. Single mRNA transfections for measuring IL-1β secretion were performed in 24-well plates in which cells were plated at 2E5 cells-per-well in 500 μl medium and treated with 500 ng mRNA, according to the MessengerMax lipofectamine protocol. Transfections of mRNA encoding GSDMD and GSDMD–TMEM106A or GSDMD–TMEM106A^ΔCT^ encoding mRNA together were performed in 96-well plates in which cells were plated at 4 × 10^4^ cells per well in 100 μl medium and treated with 120 ng mRNA. In experiments using different doses of GSDMD–TMEM106A, non-translating control mRNA was used to normalize total mRNA input across samples.

Subcellular fractionation was conducted using a Thermo Fisher Scientific Subcellular Protein Fractionation Kit for Cultured Cells (Thermo Fisher Scientific, 78840) according to the manufacturer’s instructions. Western blotting for HA was performed using anti-HA antibody (C29F4) (Cell Signaling Technology, 3724S, 1:1,000) and anti-rabbit HRP-linked secondary antibody (Cell Signaling Technology, 7074S, 1:3,000). Anti-α-tubulin antibody (DM1A) (Cell Signaling Technology, 3873, 1:1,000) was used as the subcellular fractionation control for cytoplasmic protein isolation. Anti-Na,K-ATPase antibody (Cell Signaling Technology, 3010, 1:1,000) was used as subcellular fractionation control for membrane protein isolation.

BMDMs transfected with mRNA expressing GSDMD–TMEM106A–HA (G–T–HA mRNA) or a non-translating control mRNA were observed for GSDMD–TMEM106A localization under a Nikon Ti inverted microscope using plan apo ×60 oil-immersion objective lens. Wheat germ agglutinin (WGA) with Alexa fluor 488 conjugate (Thermo Fisher Scientific, W11261) was used to stain the plasma membrane before cell fixation, using 2% PFA for 10 min, and cell permeabilization, using a PBS solution containing 2% BSA and 0.1% Triton X-100. Blocking was conducted using 2% BSA for 1 h at room temperature. HA-tagged GSDMD–TMEM106A was marked with an anti-HA antibody (16B12) conjugated to PE (1:500) (BioLegend, 901518) and visualized using a 561 nm laser. Membrane stain was visualized using a 488 nm laser.

### Pyroptosis assay, including cytokine measurement, GSDMD and CASP-1 western blotting, LDH and PI uptake

IL-1β, IL-18 and IL-6 were measured in cell-free supernatants. R&D Systems ELISA kits were used according to the manufacturer’s instructions for IL-18 (R&D Systems, DY7625-05) and IL-6 (R&D Systems, DY406-05), and BioLegend kits were used for IL-1β measurements (BioLegend, 432616). IL-1β and IL-18 were measured in the supernatant after 6 h LPS (10 ng ml^−1^ for siRNA and G-T^Stop^ experiments; 100 ng ml^−1^ for lentivirus and mRNA transfection experiments; serotype O55:B5; Enzo, ALX-581-013-L001) prime alone and after addition of nigericin (10 μM) (InvivoGen, Tlrl-nig-5) for 30 min to activate the NLRP3 inflammasome. IL-6 secretion was measured after a 6 h LPS treatment alone. Absorbance was read using the Synergy HTX plate reader at 450 nm. The samples were diluted appropriately to fall within the standard range of 2,000 to 31.3 pg ml^−1^ for IL-1β ELISAs and 250 to 7.8 pg ml^−1^ for IL-6 ELISAs. *Gsdmd-*knockout iBMDMs were gifted from the laboratory of J. Kagan and were not tested for mycoplasma contamination.

Western blots for GSDMD and CASP-1 were performed on BMDM cell lysates left at steady state, after 6 h LPS (10 ng ml^−1^) alone, or after LPS prime and nigericin treatment (10 μM) for 15 min. Whole-cell lysates were collected using NP40 lysis buffer containing 10% PMSF and 1× protease inhibitor. Western blots for GSDMD and CASP-1 were performed using anti-GSDMD antibody (E9S1X, Cell Signaling Technology, 39754, 1:1,000) and mixed anti-CASP-1 (E9R2D, Cell Signaling Technology, 83383) and anti-cleaved CASP-1 (E2G2I, Cell Signaling Technology, 89332) antibodies (1:1,000), respectively, and anti-rabbit HRP-linked secondary antibody (Cell Signaling Technology, 7074S, 1:3,000). Anti-β-actin antibody (13E5, Cell Signaling Technology, 4970, 1:1,000) was used as the loading control for whole-cell lysates.

LDH was measured in cell-free supernatants after 6 h LPS prime (10 ng ml^−1^ for siRNA and G-T^Stop^ experiments; 100 ng ml^−1^ for lentivirus and mRNA transfection experiments; serotype O55:B5; Enzo ALX-581-013-L001) and 2 h nigericin treatment (10 μM; InvivoGen, Tlrl-nig-5). Promega CytoTox 96 Non-Radioactive Cytotoxicity Assay kits were used according to the manufacturer’s instructions (Promega, G1780). Maximum LDH was determined by replacing the medium with fresh medium containing 1% Triton X-100 for 15 min. The absorbance was read using the Synergy HTX plate reader at 490 nm.

For the measurement of PI uptake, cells were plated in black 96-well plates with optically clear bottoms. After mRNA transfection for GSDMD–TMEM106A overexpression, PI uptake was assessed after 6 h LPS prime (100 ng ml^−1^) and 1 h nigericin treatment (10 μM). Diluted PI solution (1:500, Sigma-Aldrich, P4864-10ML) was added to the cell culture medium 30 min before the timepoint. The plate was centrifuged at 500*g* for 5 min and population PI staining was assayed using the Synergy HTX plate reader, conducting bottom reading of fluorescence with an excitation wavelength of 530 nm and emission wavelength of 617 nm. Maximum PI was determined after lysis of cells with 1% Triton X-100. For analysis of PI uptake in G–T^Stop^ and wild-type BMDMs, diluted PI solution (1:500) was added to the cell culture medium after LPS priming at the time of nigericin treatment (5 μM) and the plate was imaged using an Incucyte Live-Cell Analysis System with 5% CO_2_ and 37 °C incubation with phase and red channel images taken every 15 min for 5 h. The counts of red fluorescent cells in a well were quantified to determine the total PI uptake. Maximum PI uptake is reported as the percentage of the number of red fluorescent cells at a time over the number of red fluorescent cells in the same well after 5 h of nigericin treatment. For live imaging of PI uptake by G-T^Stop^ and wild-type BMDMs, after LPS priming, diluted PI solution (1:500) was added to the cell culture medium at the time of nigericin treatment (5 μM). The plate was imaged using the Nikon Ti inverted microscope with the Physik Instrumente Piezo Z motor using a ×20 objective lens under 5% CO_2_ and 37 °C incubation; bright-field and 561 nm laser channel images taken every 2.5 min for 2 h.

### LNP-encapsulated mRNA treatment

Wild-type littermate mice were randomly assigned to mRNA treatment groups and treated intravenously through tail vein injection with 100 μl of lipid nanoparticle (LNP)-encapsulated mRNA (1 mg kg^−1^) for 18 h before LPS sepsis or *S*. Typhimurium infection.

### LPS sepsis

For serum cytokine analysis, female mice treated with LNP-encapsulated mRNA were injected intraperitoneally with 100 μl LPS (*E. coli* strain O111:B4; InvivoGen, tlrl-3pelps) in PBS (3 mg kg^−1^) and monitored every 3 h for change in body temperature using a thermometer equipped with a rectal probe. Then, at 6 h after LPS injection, mice were bled retro-orbitally for serum cytokine analysis by Eve Technologies using the Mouse Cytokine Proinflammatory Focused 10-Plex Discovery Assay Array (Eve Technologies, MDF10). For assessment of survival, female mice treated with LNP-encapsulated mRNA were injected intraperitoneally with 100 μl LPS (*E. coli* strain O111:B4; InvivoGen, tlrl-3pelps) in PBS (6 mg kg^−1^). G-T^Stop^ and wild-type littermate control males were injected intraperitoneally with 100 μl LPS (*E. coli* strain O111:B4; InvivoGen, tlrl-3pelps) in PBS (15 mg kg^−1^) and the body temperature was recorded every 12 h for 24 h. All mice were regularly monitored for humane end-point survival, defined as having lost greater than 25% of their body temperature, severely hunched posturing and a severe lack of normal ambulation. Sample sizes were selected based on previous experiments.

### *S.* Typhimurium Infection

*Salmonella* was grown from glycerol stock overnight in Luria broth (LB) with ampicillin (100 μg ml^−1^) then diluted to obtain an optical density at 600 nm (OD_600_) of 0.9 (10^9^ CFU ml^−1^). Mice were injected intraperitoneally with 100 μl stationary-phase *S.* Typhimurium in PBS (10^2^ for G-T^Stop^ experiments) (10^5^ CFU for in vivo LNP experiments). Then, 24 h after infection, tissue was collected and homogenized in 2 ml of PBS. Tissue homogenate supernatants were plated onto LB agar with ampicillin (100 μg ml^−1^) for CFU calculations. Sample sizes were based on previous experiments.

### Co-immunoprecipitation

Wild-type BMDMs (10^7^) were transfected with mRNA encoding GSDMD–TMEM106A–HA, GSDMD–TMEM106A^F50G/W51G^−HA or a non-translating control. Then, 24 h later, cells were stimulated with LPS (100 ng ml^−1^) for 4 h, followed by treatment with nigericin (10 μM) and glycine (10 mM) for 30 min. Cells were lysed by adding 1 ml of Pierce IP lysis buffer (Thermo Fisher Scientific, 87787) to the dish for 30 min with gentle rocking at 4 °C. Cleared lysates were added to 25 μl of prewashed Pierce Anti-HA magnetic beads (Thermo Fisher Scientific, 88836) and incubated overnight at 4 °C for immunoprecipitation of HA-tagged protein. After thorough washing, protein was eluted from anti-HA magnetic beads by adding NuPAGE LDS sample buffer and NuPAGE sample reducing agent (Thermo Fisher Scientific, NP0007 and NP0004) and heating at 70 °C for 10 min. Immunoblotting was conducted using an antibody against cleaved GSDMD (Cell Signaling Technology, 10137S, 1:1,000), and against the HA-tag (C29F4) (Cell Signaling Technology, 3724S, 1:1,000) with anti-rabbit HRP-linked secondary antibody (Cell Signaling Technology, 7074S, 1:3,000). Immunoblotting of input control lysates was performed using an anti-GSDMD antibody (E9S1X, Cell Signaling Technology, 39754, 1:1,000), β-actin antibody (13E5, Cell Signaling Technology, 4970, 1:1,000) and an HA-tag antibody (C29F4, Cell Signaling Technology, 3724S, 1:1,000).

### Lentiviral transduction

Plasmids and lentiviral packaging were designed and performed by Vector Builder. For immunofluorescence microscopy, BMDMs were plated 2 × 10^5^ cells per well in a black 24-well-plate optically clear bottom pretreated with poly-l-lysine. For inflammasome activation and IL-1β-release assays, BMDMs were plated 2 × 10^5^ cells per well in standard tissue-culture-treated 24-well-plates. For subcellular protein fractionation BMDMs were plated at 1.5 × 10^6^ cells per well in standard tissue-culture-treated 6-well plates. In all experiments cells were transduced at a multiplicity of infection of 10 in medium containing polybrene (8 μg ml^−1^). Fresh medium was added 3 h after transduction and cells were assayed 72 h later.

BMDMs transduced with lentivirus expressing GSDMD–TMEM106A–HA (Lenti(G-T-HA)) or an empty vector control were observed for GSDMD–TMEM106A localization under a Nikon Ti inverted microscope with the Physik Instrumente Piezo Z motor using a plan apo ×100 oil-immersion objective lens. WGA with Alexa fluor 488 conjugate (Thermo Fisher Scientific, W11261) was used to stain the plasma membrane before cell fixation, using 4% PFA for 10 min, and cell permeabilization, using a PBS solution containing 2% BSA and 0.1% Triton X-100. Blocking was conducted using 2% BSA for 1 h at room temperature. HA-tagged GSDMD–TMEM106A was marked with an anti-HA antibody (16B12) conjugated to PE (1:500, BioLegend, 901518) and visualized using the 561 nm laser. Membrane staining was visualized using a 488 nm laser.

Subcellular fractionation was conducted using the Thermo Fisher Scientific Subcellular Protein Fractionation Kit for Cultured Cells (Thermo Fisher Scientific, 78840) according to the manufacturer’s instructions. Western blotting for HA was performed using anti-HA antibody (C29F4, Cell Signaling Technology, 3724S, 1:1,000) and anti-rabbit HRP-linked secondary antibody (Cell Signaling Technology, 7074S, 1:3,000). Anti-α-tubulin antibody (DM1A, Cell Signaling Technology, 3873, 1:1,000) was used as the subcellular fractionation control for cytoplasmic protein isolation. Anti-Na,K-ATPase antibody (Cell Signaling Technology, 3010, 1:1,000) was used as subcellular fractionation control for membrane protein isolation.

### Doxycycline-inducible GSDMD-NT experiments

iBMDMs stably expressing doxycycline-inducible GSDMD-NT^40^ were gifted from the laboratory of J. Kagan and were not tested for mycoplasma contamination. Cells were plated at 3 × 10^4^ cells per well in a black 96-well plate with optically clear bottoms and maintained in medium containing puromycin and G418. Once cells adhered (over 12 h after plating), mRNA was transfected for GSDMD–TMEM106A, GSDMD–TMEM106A^ΔCT^ or a non-translating control for 24 h. To induce GSDMD-NT expression, cells were treated with doxycycline hyclate (Sigma-Aldrich, D9891-1G) in fresh medium (0.25 µg ml^−1^). Diluted PI solution (1:300, Sigma-Aldrich, P4864-10ML) was added to the cell culture medium at the time of doxycycline treatment and the plate was imaged using the Incucyte Live-Cell Analysis System under 5% CO_2_ and 37 °C incubation; phase and red channel images were taken every hour for 24 h. The count of red fluorescent cells in a well was quantified to determine PI uptake. Maximum PI uptake is reported as the percentage of the number of red fluorescent cells at a time over the number of red fluorescent cells in the same well after 24 h of doxycycline treatment.

### Recombinant protein constructs

Full-length GSDMD was cloned into the pDB.His.SUMO vector using Gibson Assembly Master Mix (New England Biolabs, E2611L). GSDMD–TMEM106A–FLAG (rG–T–FLAG) or rGSDMD–TMEM106A^ΔCT^−FLAG were cloned into the pDB.His.MBP-3C vector, downstream of the N-terminal His_6_–maltose-binding protein (MBP) tag and human rhinovirus 3C protease site, using the Gibson Assembly Master Mix.

### Expression and purification of proteins in bacteria

pDB-His-SUMO-GSDMD, pDB.His.MBP-3C-rG-T-FLAG and pDB.His.MBP-3C-rG-T^Δ^^CT^-FLAG constructs were transformed into *E. coli* BL21 (DE3) cells (Agilent Technologies, 230280). Transformants were plated and incubated overnight at 37 °C. Single colonies were inoculated into LB medium containing 50 μg ml^−1^ kanamycin and cultured at 37 °C. At an OD_600_ of 0.6, protein expression was induced with 500 μM isopropyl β-d-1-thiogalactopyranoside, and cells were grown for 16 h at 18 °C before collection.

For His-SUMO-GSDMD, cells were pelleted by centrifugation at 4,000*g* for 30 min and resuspended in buffer A (40 mM HEPES, pH 7.0, 150 mM NaCl) supplemented with 5 mM imidazole. Cells were lysed by sonication, and His–SUMO-tagged GSDMD was captured on Ni-NTA resin and eluted with buffer A containing 300 mM imidazole. The His–SUMO tag was removed by ULP1 protease at 4 °C for 4 h. Cleaved tag was separated using Ni-NTA, and the flow-through containing GSDMD was further purified on the Superdex 200 Increase 10/300 GL size-exclusion column (Cytiva) equilibrated in buffer A. Peak fractions were assessed by SDS–PAGE and snap-frozen for downstream assays.

For pDB-His-MBP-3C-rG-T-FLAG or pDB.His.MBP-3C-rG-T^Δ^^CT^-FLAG, cells were pelleted by centrifugation at 4,000*g* for 30 min and resuspended in buffer A. Cells were lysed by sonication (2 s on, 8 s off; 5 min total on time; 40% amplitude) on ice, and clarified by centrifugation at 18,000*g* for 1 h. The supernatant was incubated with anti-FLAG G1 affinity resin (GenScript, L00432-25) for 2 h at 4 °C with gentle rotation. After extensive washing, proteins were eluted with buffer A containing 150 ng µl^−1^ 3×FLAG peptide (Sigma-Aldrich, F4799-25MG), analysed for purity and snap-frozen for subsequent assays.

### Expression and purification of proteins in mammalian cells

pcDNA3.1-GSDMD-Flag constructs were transfected into Expi293 cells that were maintained in 1,000 ml Expi293 expression medium, fed with 6 mM KCl and grown to 2.5 × 10^6^ cells per ml, using polyethylenimine (PEI, Polysciences). At 12 h after transfection, the culture was fed with 10 mM sodium butyrate and 10 ml of a 45% d-(+)-glucose solution. The cells were then grown for an additional 2 days before being collected by centrifugation at 4,000*g* for 30 min.

The cell pellet was resuspended in buffer A and lysed by sonication (2 s on, 8 s off, for a total on time of 3.5 min at 40% power). The lysates were clarified by ultracentrifugation at 40,000 rpm for 1 h. The supernatant was collected and incubated with Flag G1 affinity resin (GenScript, L00432-25) for 2 h at 4 °C with gentle rotation. After washing, the protein was eluted using buffer A with 150 ng μl^−1^ 3× Flag peptide (Sigma-Aldrich, F4799-25MG). The eluted GSDMD protein was snap-frozen for other assays. To obtain highly palmitoylated GSDMD, ROT (Sigma-Aldrich, R8875-1G) at 10 μM was used to treat Expi293 cells for 4 h before collection.

### Liposome leakage assay

Liposomes were prepared as previously described^39,91^. In brief, cardiolipin (CL), 1-palmitoyl-2-oleoyl-*sn*-glycero-3-phosphoethanolamine (PE) and phosphatidylcholine (PC) (Avanti Polar Lipids) were mixed at a mass ratio of 5:8:4. The solvent was evaporated under a gentle stream of N_2_ gas, and the dried lipid film was resuspended in 200 μl buffer B (20 mM HEPES, pH 7.4, 150 mM NaCl, 50 mM sodium citrate and 15 mM TbCl_3_). The suspension was extruded 45 times through 100 nm Whatman Nuclepore Track-Etched membranes to obtain homogeneous liposomes. The filtered suspension was purified through a size-exclusion column (Superose 6, 10/300 GL) in buffer C (20 mM HEPES, 150 mM NaCl) to remove TbCl_3_ outside liposomes. Fractions close to void were pooled to produce a stock of PC/PE/CL liposomes.

Bacteria or mammalian expressed full-length GSDMD was precleaved by CASP-1 for 1 h. His–MBP–3C–G–T–Flag or His–MBP–3C–G–T^ΔCT^−Flag was precleaved by 3C for 30 min. Catalytically active CASP-1 was expressed and purified as previously described^91^. In brief, the p20 and p10 subunits (without tags) were individually expressed in *E. coli* BL21 (DE3) as inclusion bodies. The p20–p10 complex was assembled by denaturation and refolding, and further purified by HiTrap SP cation-exchange chromatography (GE Healthcare Life Sciences).

For leakage assays, liposomes were diluted four times in buffer D (20 mM HEPES, 150 mM NaCl and 15 μM dipicolinic acid). Leakage of Tb^3+^ from liposomes was monitored by the increase in fluorescence after binding to dipicolinic acid in buffer D. Pre-cleaved proteins were added to 384-well plates (Corning, 3820) containing PC/PE/CL liposomes. The fluorescence intensity was recorded at 545 nm (excitation 276 nm) immediately after mixing and monitored for 2 h on a SYNERGY microplate reader (Biotek). The baseline emission before protein addition was defined as *F*_0_. After 2 h, 0.1% Triton X-100 was added to achieve complete Tb^3+^ release, and the mean of the top three fluorescence readings was defined as *F*_100_. The percentage of Tb^3+^ release at each timepoint is defined as follows: Tb^3+^ release (%) = (*F* − *F*_0_) × 100/(*F*_100_ − *F*_0_).

### Protein–lipid binding assay

Lipid-binding assay for rG–T–Flag was performed using membrane lipid strips (Echelon Biosciences, P-6002) according to the manufacturer’s instructions. In brief, lipid strip membranes were blocked with 3% BSA in PBS containing 0.1% Tween-20 (PBST) for 1 h at room temperature. The strips were then incubated for 1 h with pre-cleaved rG–T–Flag (His–MBP–3C–rG–T–Flag was pre-cleaved with 3C protease for 30 min to remove the His–MBP–3C tag) at 2 μg ml^−1^ in 3% BSA/PBST. After three washes with PBST (5 min each), bound proteins were detected using anti-Flag M2-HRP antibody (1:1,000). All steps were performed at room temperature.

### Protein structure prediction and visualization

Prediction of three-dimensional protein structures was performed using AlphaFold 3^92^ with AlphaFold Server (https://alphafoldserver.com/) using a random seed value of 9999. Protein structure images were created using Mol*^93^ and the RCSB PDB platform^94^ (https://www.rcsb.org/).

### Schematic design

Schematics for Figs. 1a, 2k and 3a and Extended Data Figs. 3a and 9a were created using BioRender. All other schematics were created using PowerPoint or Inkscape software.

### Data analysis

All sequencing data analysis was performed on a Linux GNU Pop!_OS LTS 22.04 64-bit system or Ubuntu 26.04 LTS 64-bit system. Python analysis was performed using Anaconda Distribution version ≤26.1.1 and Python programming language version ≤3.13.9. Analysis in R was performed using R version ≤4.5.2 running in R Studio version ≤2026.05.0 Build 218. Graphs generated in R were produced using the ggplot2 suite^95^ version ≤4.0.3 and ComplexHeatmap^96^ v.2.26.1. Microsoft Excel (v.2607, build 16.0.20228.20110) 64-bit and LibreOffice Calc 26.2.4.2 (X86_64) were used for table generation and data organization. Microsoft Windows 11 Pro 24H2 OS and MacOS BigSur v.11.6 were used for additional data analysis. AlphaFold 3 folding was run using AlphaFold Server (https://alphafoldserver.com/welcome). Mol* and the RCSB PDB platform for AlphaFold 3 result visualization were accessed at https://www.rcsb.org/3d-view.

Except where otherwise indicated, statistical significance for experiments with multiple groups and multiple independent variables was determined using two-way ANOVA with Sidak’s multiple-comparison test. One-way ANOVA with Tukey’s multiple-comparison test was used to determine statistical significance for experiments with multiple groups and a single independent variable. Unpaired *t*-tests were performed to determine significance between two variables. All non-R/Python adjusted *P* values were calculated using GraphPad Prism v.10.0.2. GraphPad Prism was run on MacOS BigSur v.11.6.

### Ethics

All mouse experiments were performed in accordance with protocols approved by the Harvard Medical School Institutional Animal Care and Use Committee.

### Reporting summary

Further information on research design is available in the Nature Portfolio Reporting Summary linked to this article.

## Online content

Any methods, additional references, Nature Portfolio reporting summaries, source data, extended data, supplementary information, acknowledgements, peer review information; details of author contributions and competing interests; and statements of data and code availability are available at https://doi.org/10.1038/s41586-026-10982-x.

## Supplementary information

Supplementary Information Supplementary Figs. 1–5, the Supplementary Discussion, and the legends for Supplementary Tables 1–12 and Supplementary Videos 1 and 2.

Reporting Summary

Supplementary Table 1 Metadata and quality-control metrics for individual mouse macrophage long-read direct RNA-seq replicates, including the number of reads per sample, sample identity and the percentage of reads mapping to the reference genome.

Supplementary Table 2 Chimeric RNA detection results from running LongGF, Genion and JAFFAL in K562 cancer cell long-read direct RNA-seq data used to optimize parameters for analysis of mouse and human macrophage long-read direct RNA-seq data.

Supplementary Table 3 Summary information for chimeric RNA reads detected by LongGF, Genion and JAFFAL in mouse macrophage long-read direct RNA-seq data. Chimeric RNA data are corrected for biological order of the chimeric RNA parent genes.

Supplementary Table 4 Table containing tool-level chimeric RNA detection results from running LongGF, Genion and JAFFAL in mouse macrophage long-read direct RNA-seq data.

Supplementary Table 5 Table containing metadata metrics for individual mouse macrophage short-read RNA-seq replicates, including the number of reads per sample and sample identity.

Supplementary Table 6 Tool-level chimeric RNA detection results from running Arriba FusionCatcher, STAR-Fusion, STAR-SEQR, JAFFA and Pizzly in mouse macrophage short-read direct RNA-seq data.

Supplementary Table 7 Chimeric RNA cross-detected in mouse macrophage long-read RNA-seq data and either short-read mouse macrophage RNA-seq data (first column), NanoString nCounter (second column) or both short-read mouse macrophage RNA-seq data and NanoString nCounter (third column).

Supplementary Table 8 Mouse chimeric junction sequences used for NanoString nCounter probe design.

Supplementary Table 9 Primer sequences used for the PCR and Sanger sequencing of chimeric RNA.

Supplementary Table 10 Metadata and quality control metrics for individual human macrophage long-read direct RNA-seq replicates, including the number of reads per sample, sample identity and the percentage of reads mapping to the reference genome.

Supplementary Table 11 Summary information for chimeric RNA reads detected by LongGF, Genion and JAFFAL in human macrophage long-read direct RNA-seq data. Chimeric RNA data are corrected for biological order of the chimeric RNA parent genes.

Supplementary Table 12 Table of chimeric RNA conservation data for chimeric RNA conserved between mouse and human macrophages in long-read direct RNA-seq data.

Supplementary Video 1 Timelapse video of PI uptake by wild-type BMDMs. Recording started at the time of nigericin treatment. Data are related to the left panels in Fig. 5h.

Supplementary Video 2 Timelapse video of PI uptake by G-T^Stop^ BMDMs. Recording started at the time of nigericin treatment. Data are related to the right panels in Fig. 5h

## Source data

Source Data Fig. 1

Source Data Fig. 2

Source Data Fig. 3

Source Data Fig. 4

Source Data Fig. 5

Source Data Extended Data Fig. 1

Source Data Extended Data Fig. 2

Source Data Extended Data Fig. 3

Source Data Extended Data Fig. 4

Source Data Extended Data Fig. 5

Source Data Extended Data Fig. 7

Source Data Extended Data Fig. 8

Source Data Extended Data Fig. 9

Source Data Extended Data Fig. 10

## Data Availability

Long-read direct RNA-seq data have been deposited in the Gene Expression Omnibus (GEO) under accession code GSE267147 (mouse) and GSE277057 (human). Short-read RNA-seq data have been deposited in the GEO under accession code GSE324139. Hi-C sequencing data have been deposited in the GEO under accession code GSE324391. K562 cell data were downloaded from GEO accession GSE123191 (ref. ^69^). GENCODE reference files for GENCODE M28 release (GRCm39) and GENCODE 43 release (GRCh38.p13) are available online (https://www.gencodegenes.org/). UCSC reference files are available online (https://genome.ucsc.edu/) and can be accessed through the Table Browser (https://genome.ucsc.edu/cgi-bin/hgTables). Ensembl reference files are available online (https://www.ensembl.org/info/data/ftp/index.html?redirect=no) and can be downloaded through the ftp site (https://ftp.ensembl.org/pub/). Source data are provided with this paper.

## Code availability

Custom code for the TYPHON wrapper script is available at GitHub (https://github.com/erenada/TYPHON).

## References

[1] Liu, X. et al. Inflammasome-activated gasdermin D causes pyroptosis by forming membrane pores. *Nature* **535**, 153–158 (2016). 10.1038/nature18629 PMC5539988 27383986

[2] Shi, J. et al. Cleavage of GSDMD by inflammatory caspases determines pyroptotic cell death. *Nature* **526**, 660–665 (2015). 10.1038/nature15514 26375003

[3] Michaeli, S. *Trans*-splicing in trypanosomes: machinery and its impact on the parasite transcriptome. *Fut. Microbiol.* **6**, 459–474 (2011). 10.2217/fmb.11.20 21526946

[4] Dorn, R., Reuter, G. & Loewendorf, A. Transgene analysis proves mRNA trans-splicing at the complex mod(mdg4) locus in *Drosophila*. *Proc. Natl Acad. Sci. USA* **98**, 9724–9729 (2001). 10.1073/pnas.151268698 PMC55520 11493677

[5] Daley, G. Q., Van Etten, R. A. & Baltimore, D. Induction of chronic myelogenous leukemia in mice by the P210bcr/abl gene of the Philadelphia chromosome. *Science* **247**, 824–830 (1990). 10.1126/science.2406902 2406902

[6] Nowell, P. C. & Hungerford, D. A. Chromosome studies on normal and leukemic human leukocytes. *J. Natl Cancer Inst.* **25***,* 85–109 (1960). 14427847

[7] Li, H., Wang, J., Mor, G. & Sklar, J. A neoplastic gene fusion mimics *trans*-splicing of RNAs in normal human cells. *Science* **321**, 1357–1362 (2008). 10.1126/science.1156725 18772439

[8] Bracht, J. R. et al. Chromosome fusions triggered by noncoding RNA. *RNA Biol.* **14**, 620–631 (2017). 10.1080/15476286.2016.1195940 PMC5449082 27267579

[9] Yuan, H. et al. A chimeric RNA characteristic of rhabdomyosarcoma in normal myogenesis process. *Cancer Discov.* **3**, 1394–1403 (2013). 10.1158/2159-8290.CD-13-0186 24089019

[10] Cocquet, J., Chong, A., Zhang, G. & Veitia, R. A. Reverse transcriptase template switching and false alternative transcripts. *Genomics* **88**, 127–131 (2006). 10.1016/j.ygeno.2005.12.013 16457984

[11] Heyer, E. E. et al. Diagnosis of fusion genes using targeted RNA sequencing. *Nat. Commun.* 10.1038/s41467-019-09374-9 (2019). PMC6437215 30918253

[12] Latysheva, N. S. & Babu, M. M. Discovering and understanding oncogenic gene fusions through data intensive computational approaches. *Nucleic Acids Res.* **44**, 4487–4503 (2016). 10.1093/nar/gkw282 PMC4889949 27105842

[13] Almeida da Paz, M., Warger, S. & Taher, L. Disregarding multimappers leads to biases in the functional assessment of NGS data. *BMC Genom.* 10.1186/s12864-024-10344-9 (2024). PMC11078754 38720252

[14] Chen, C., Haddox, S., Tang, Y., Qin, F. & Li, H. Landscape of chimeric RNAs in non-cancerous cells. *Genes*10.3390/genes12040466 (2021). PMC8064075 33805149

[15] Singh, S. et al. The landscape of chimeric RNAs in non-diseased tissues and cells. *Nucleic Acids Res.* **48**, 1764–1778 (2020). 10.1093/nar/gkz1223 PMC7038929 31965184

[16] Liu, Q. et al. LongGF: computational algorithm and software tool for fast and accurate detection of gene fusions by long-read transcriptome sequencing. *BMC Genom.* **21**, 793 (2020). 10.1186/s12864-020-07207-4 PMC7771079 33372596

[17] Davidson, N. M. et al. JAFFAL: detecting fusion genes with long-read transcriptome sequencing. *Genome Biol.* **23**, 10 (2022). 10.1186/s13059-021-02588-5 PMC8739696 34991664

[18] Karaoglanoglu, F., Chauve, C. & Hach, F. Genion, an accurate tool to detect gene fusion from long transcriptomics reads. *BMC Genom.* **23**, 129 (2022). 10.1186/s12864-022-08339-5 PMC8842519 35164688

[19] Drexler, H. L. et al. Revealing nascent RNA processing dynamics with nano-COP. *Nat. Protoc.* **16**, 1343–1375 10.1038/s41596-020-00469-y (2021). PMC8713461 33514943

[20] Uhrig, S. et al. Accurate and efficient detection of gene fusions from RNA sequencing data. *Genome Res.* 10.1101/gr.257246.119 (2021). PMC7919457 33441414

[21] Nicorici, D. et al. FusionCatcher—a tool for finding somatic fusion genes in paired-end RNA-sequencing data. Preprint at *bioRxiv* 10.1101/011650 (2014).

[22] Haas, B. J. et al. Accuracy assessment of fusion transcript detection via read-mapping and de novo fusion transcript assembly-based methods. *Genome Biol.* **20**, 213 (2019). 10.1186/s13059-019-1842-9 PMC6802306 31639029

[23] Melsted, P. et al. Fusion detection and quantification by pseudoalignment. Preprint at *bioRxiv* 10.1101/166322 (2017).

[24] Davidson, N. M., Majewski, I. J. & Oshlack, A. JAFFA: high sensitivity transcriptome-focused fusion gene detection. *Genome Med.* 10.1186/S13073-015-0167-X (2015). PMC4445815 26019724

[25] Caldas, P. et al. Transcription readthrough is prevalent in healthy human tissues and associated with inherent genomic features. *Commun. Biol.* **7**, 100 (2024). 10.1038/s42003-024-05779-5 PMC10789751 38225287

[26] Ribeiro, D. M., Ziyani, C. & Delaneau, O. Shared regulation and functional relevance of local gene co-expression revealed by single cell analysis. *Commun. Biol.* **5**, 876 (2022). 10.1038/s42003-022-03831-w PMC9418141 36028576

[27] Zhang, X. et al. Inactivation of TMEM106A promotes lipopolysaccharide-induced inflammation via the MAPK and NF-κB signaling pathways in macrophages. *Clin. Exp. Immunol.* **203**, 125–136 (2021). 10.1111/cei.13528 PMC7744488 33006758

[28] Kotake, Y. et al. Splicing factor SF3b as a target of the antitumor natural product pladienolide. *Nat. Chem. Biol.* **3**, 570–575 (2007). 10.1038/nchembio.2007.16 17643112

[29] Cretu, C. et al. Structural basis of splicing modulation by antitumor macrolide compounds. *Mol. Cell* **70**, 265–273 (2018). 10.1016/j.molcel.2018.03.011 29656923

[30] Perry, R. P. & Kelley, D. E. Inhibition of RNA synthesis by actinomycin D: characteristic dose-response of different RNA species. *J. Cell. Physiol.* **76**, 127–139 (1970). 10.1002/jcp.1040760202 5500970

[31] Ling, J. Q. et al. CTCF mediates interchromosomal colocalization between Igf2/H19 and Wsb1/Nf1. *Science* **312**, 269–272 (2006). 10.1126/science.1123191 16614224

[32] Kayagaki, N. et al. Caspase-11 cleaves gasdermin D for non-canonical inflammasome signalling. *Nature* **526**, 666–671 (2015). 10.1038/nature15541 26375259

[33] Netea, M. G. et al. IL-1β processing in host defense: beyond the inflammasomes. *PLoS Pathog.* **6**, e1000661 (2010). 10.1371/journal.ppat.1000661 PMC2829053 20195505

[34] Schultz, M. J. et al. Role of interleukin-1 in the pulmonary immune response during *Pseudomonas aeruginosa* pneumonia. *Am. J. Physiol. Lung Cell. Mol. Physiol.* **282**, L285–L290 (2002). 10.1152/ajplung.00461.2000 11792633

[35] Chen, K. W. et al. Noncanonical inflammasome signaling elicits gasdermin D-dependent neutrophil extracellular traps. *Sci. Immunol.* **3**, eaar6676 (2018). 10.1126/sciimmunol.aar6676 30143554

[36] Leventis, P. A. & Grinstein, S. The distribution and function of phosphatidylserine in cellular membranes. *Annu. Rev. Biophys.* **39**, 407–427 (2010). 10.1146/annurev.biophys.093008.131234 20192774

[37] Van Meer, G., Voelker, D. R. & Feigenson, G. W. Membrane lipids: where they are and how they behave. *Nat. Rev. Mol. Cell Biol.* **9**, 112–124 (2008). 10.1038/nrm2330 PMC2642958 18216768

[38] Yu, P. et al. Pyroptosis: mechanisms and diseases. *Signal Transduct. Target. Ther.* **6**, 128 (2021). 10.1038/s41392-021-00507-5 PMC8005494 33776057

[39] Xia, S. et al. Gasdermin D pore structure reveals preferential release of mature interleukin-1. *Nature* **593**, 607–611 (2021). 10.1038/s41586-021-03478-3 PMC8588876 33883744

[40] Evavold, C. L. et al. Control of gasdermin D oligomerization and pyroptosis by the Ragulator-Rag-mTORC1 pathway. *Cell* **184**, 4495–4511 (2021). 10.1016/j.cell.2021.06.028 PMC8380731 34289345

[41] Jackson, R. et al. The translation of non-canonical open reading frames controls mucosal immunity. *Nature* **564**, 434–438 (2018). 10.1038/s41586-018-0794-7 PMC6939389 30542152

[42] Brubaker, S. W., Gauthier, A. E., Mills, E. W., Ingolia, N. T. & Kagan, J. C. A bicistronic MAVS transcript highlights a class of truncated variants in antiviral immunity. *Cell*10.1016/j.cell.2014.01.021 (2014). PMC3959641 24529381

[43] Anderson, D. M. et al. A micropeptide encoded by a putative long noncoding RNA regulates muscle performance. *Cell* **160**, 595–606 (2015). 10.1016/j.cell.2015.01.009 PMC4356254 25640239

[44] Wang, Y. & Wang, Z. Efficient backsplicing produces translatable circular mRNAs. *RNA*10.1261/rna.048272.114 (2015). PMC4338345 25449546

[45] Memczak, S. et al. Circular RNAs are a large class of animal RNAs with regulatory potency. *Nature*10.1038/nature11928 (2013). 23446348

[46] Chen, C.-K. et al. Structured elements drive extensive circular RNA translation. *Mol. Cell* **81**, 4300–4318 (2021). 10.1016/j.molcel.2021.07.042 PMC8567535 34437836

[47] Jeck, W. R. et al. Circular RNAs are abundant, conserved, and associated with ALU repeats. *RNA* **19**, 141–157 (2013). 10.1261/rna.035667.112 PMC3543092 23249747

[48] Rowley, J. D. & Blumenthal, T. The cart before the horse. *Science* **321**, 1302–1304 (2008). 10.1126/science.1163791 18772424

[49] Engreitz, J. M., Agarwala, V. & Mirny, L. A. Three-dimensional genome architecture influences partner selection for chromosomal translocations in human disease. *PLoS ONE* **7**, e44196 (2012). 10.1371/journal.pone.0044196 PMC3460994 23028501

[50] Lin, C. et al. Nuclear receptor-induced chromosomal proximity and DNA breaks underlie specific translocations in cancer. *Cell* **139**, 1069–1083 (2009). 10.1016/j.cell.2009.11.030 PMC2812435 19962179

[51] Marek, L. R. & Kagan, J. C. Phosphoinositide Binding by the Toll Adaptor dMyD88 controls antibacterial responses in *Drosophila*. *Immunity* **36**, 612–622 (2012). 10.1016/j.immuni.2012.01.019 PMC3354765 22464168

[52] Miao, R. et al. Gasdermin D permeabilization of mitochondrial inner and outer membranes accelerates and enhances pyroptosis. *Immunity* **56**, 2523–2541 (2023). 10.1016/j.immuni.2023.10.004 PMC10872579 37924812

[53] Chu, X. et al. Gasdermin D-mediated pyroptosis is regulated by AMPK-mediated phosphorylation in tumor cells. *Cell Death Dis.* **14**, 469 (2023). 10.1038/s41419-023-06013-6 PMC10372026 37495617

[54] Devant, P. et al. Gasdermin D pore-forming activity is redox-sensitive. *Cell Rep.* 10.1016/j.celrep.2023.112008 (2023). PMC9947919 36662620

[55] Humphries, F. et al. Succination inactivates gasdermin D and blocks pyroptosis. *Science*10.1126/science.abb9818 (2020). PMC8744141 32820063

[56] Shi, Y. et al. E3 ubiquitin ligase SYVN1 is a key positive regulator for GSDMD-mediated pyroptosis.* Cell Death Dis.* 10.1038/s41419-022-04553-x (2022). PMC8814081 35115505

[57] Chu, X. et al. Ubiquitination of gasdermin D N-terminal domain directs its membrane translocation and pore formation during pyroptosis. *Cell Death Dis.* **16**, 181 (2025). 10.1038/s41419-025-07475-6 PMC11914233 40097387

[58] Frankish, A. et al. GENCODE: reference annotation for the human and mouse genomes in 2023. *Nucleic Acids Res.* **51**, D942–D949 (2023). 10.1093/nar/gkac1071 PMC9825462 36420896

[59] Nassar, L. R. et al. The UCSC Genome Browser database: 2023 update. *Nucleic Acids Res.* **51**, D1188–D1195 (2023). 10.1093/nar/gkac1072 PMC9825520 36420891

[60] De Coster, W., D’Hert, S., Schultz, D. T., Cruts, M. & Van Broeckhoven, C. NanoPack: visualizing and processing long-read sequencing data. *Bioinformatics* **34**, 2666–2669 (2018). 10.1093/bioinformatics/bty149 PMC6061794 29547981

[61] Li, H. Minimap2: pairwise alignment for nucleotide sequences. *Bioinformatics* **34**, 3094–3100 (2018). 10.1093/bioinformatics/bty191 PMC6137996 29750242

[62] Li, H. et al. The Sequence Alignment/Map format and SAMtools. *Bioinformatics* **25**, 2078–2079 (2009). 10.1093/bioinformatics/btp352 PMC2723002 19505943

[63] Patro, R., Duggal, G., Love, M. I., Irizarry, R. A. & Kingsford, C. Salmon provides fast and bias-aware quantification of transcript expression. *Nat. Methods* **14**, 417–419 (2017). 10.1038/nmeth.4197 PMC5600148 28263959

[64] Soneson, C., Love, M. I. & Robinson, M. D. Differential analyses for RNA-seq: transcript-level estimates improve gene-level inferences. *F1000Research* 10.12688/F1000RESEARCH.7563.2 (2016). PMC4712774 26925227

[65] Love, M. I., Huber, W. & Anders, S. Moderated estimation of fold change and dispersion for RNA-seq data with DESeq2. *Genome Biol.* 10.1186/s13059-014-0550-8 (2014). PMC4302049 25516281

[66] Dyer, S. C. et al. Ensembl 2025. *Nucleic Acids Res.* **53**, D948–D957 (2025). 10.1093/nar/gkae1071 PMC11701638 39656687

[67] Zheng, G. X. Y. et al. Massively parallel digital transcriptional profiling of single cells. *Nat. Commun.* 10.1038/ncomms14049 (2017). PMC5241818 28091601

[68] Lopez, F. et al. Explore, edit and leverage genomic annotations using Python GTF toolkit. *Bioinformatics* **35**, 3487–3488 10.1093/bioinformatics/btz116 (2019). 30768152

[69] Drexler, H. L., Choquet, K. & Churchman, L. S. Splicing kinetics and coordination revealed by direct nascent RNA sequencing through nanopores. *Mol. Cell* **77**, 985–998 (2020). 10.1016/j.molcel.2019.11.017 PMC7060811 31839405

[70] Shen, W., Le, S., Li, Y. & Hu, F. SeqKit: a cross-platform and ultrafast toolkit for FASTA/Q file manipulation. *PLoS ONE*10.1371/journal.pone.0163962 (2016). PMC5051824 27706213

[71] Camacho, C. et al. BLAST+: architecture and applications. *BMC Bioinform.* 10.1186/1471-2105-10-421 (2009). PMC2803857 20003500

[72] Neph, S. et al. BEDOPS: high-performance genomic feature operations. *Bioinformatics* **28**, 1919–1920 (2012). 10.1093/bioinformatics/bts277 PMC3389768 22576172

[73] Quinlan, A. R. & Hall, I. M. BEDTools: a flexible suite of utilities for comparing genomic features. *Bioinformatics* **26**, 841–842 (2010). 10.1093/bioinformatics/btq033 PMC2832824 20110278

[74] Sievers, F. et al. Fast, scalable generation of high-quality protein multiple sequence alignments using Clustal Omega. *Mol. Syst. Biol.* **7**, 539 10.1038/msb.2011.75 (2011). PMC3261699 21988835

[75] Larkin, M. A. et al. Clustal W and Clustal X version 2.0. *Bioinformatics* **23**, 2947–2948 (2007). 10.1093/bioinformatics/btm404 17846036

[76] Cock, P. J. A. et al. Biopython: freely available Python tools for computational molecular biology and bioinformatics. *Bioinformatics* **25**, 1422–1423 (2009). 10.1093/bioinformatics/btp163 PMC2682512 19304878

[77] Singh, U. & Wurtele, E. S. orfipy: a fast and flexible tool for extracting ORFs. *Bioinformatics* **37**, 3019–3020 (2021). 10.1093/bioinformatics/btab090 PMC8479652 33576786

[78] Browaeys, R., Saelens, W. & Saeys, Y. NicheNet: modeling intercellular communication by linking ligands to target genes. *Nat. Methods* **17**, 159–162 (2020). 10.1038/s41592-019-0667-5 31819264

[79] Lawrence, M., Gentleman, R. & Carey, V. rtracklayer: an R package for interfacing with genome browsers. *Bioinformatics* **25**, 1841–1842 (2009). 10.1093/bioinformatics/btp328 PMC2705236 19468054

[80] Bray, N. L., Pimentel, H., Melsted, P. & Pachter, L. Near-optimal probabilistic RNA-seq quantification. *Nat. Biotechnol.* **34**, 525–527 10.1038/nbt.3519 (2016). 27043002

[81] Marsh, S. E. scCustomize: custom visualizations & functions for streamlined analyses of single cell sequencing. *Zenodo*10.5281/zenodo.5706430 (2021).

[82] Ritchie, M. E. et al. limma powers differential expression analyses for RNA-sequencing and microarray studies. *Nucleic Acids Res.* **43**, e47 (2015). 10.1093/nar/gkv007 PMC4402510 25605792

[83] Class, C. A., Lukan, C. J., Bristow, C. A. & Do, K. A. Easy NanoString nCounter data analysis with the NanoTube. *Bioinformatics*10.1093/bioinformatics/btac762 (2023). PMC9805552 36440915

[84] Johanson, T. M. et al. Transcription-factor-mediated supervision of global genome architecture maintains B cell identity. *Nat. Immunol.* **19**, 1257–1264 (2018). 10.1038/s41590-018-0234-8 30323344

[85] Lun, A. T. L. & Smyth, G. K. diffHic: a Bioconductor package to detect differential genomic interactions in Hi-C data. *BMC Bioinform.* **16**, 258 (2015). 10.1186/s12859-015-0683-0 PMC4539688 26283514

[86] Martin, M. Cutadapt removes adapter sequences from high-throughput sequencing reads. *EMBnet* **17**, 10–11 (2011).

[87] Langmead, B. & Salzberg, S. L. Fast gapped-read alignment with Bowtie 2. *Nat. Methods* **9**, 357–359 (2012). 10.1038/nmeth.1923 PMC3322381 22388286

[88] Durand, N. C. et al. Juicer provides a one-click system for analyzing loop-resolution Hi-C experiments. *Cell Syst.* **3**, 95–98 (2016). 10.1016/j.cels.2016.07.002 PMC5846465 27467249

[89] Van Der Weide, R. H. et al. Hi-C analyses with GENOVA: a case study with cohesin variants. *NAR Genom. Bioinform.* **3**, lqab040 (2021). 10.1093/nargab/lqab040 PMC8140737 34046591

[90] Liao, Y., Smyth, G. K. & Shi, W. The R package Rsubread is easier, faster, cheaper and better for alignment and quantification of RNA sequencing reads. *Nucleic Acids Res.* 10.1093/nar/gkz114 (2019). PMC6486549 30783653

[91] Du, G. et al. ROS-dependent *S*-palmitoylation activates cleaved and intact gasdermin D. *Nature* **630**, 437–446 (2024). 10.1038/s41586-024-07373-5 PMC11283288 38599239

[92] Abramson, J. et al. Accurate structure prediction of biomolecular interactions with AlphaFold 3. *Nature* **630**, 493–500 (2024). 10.1038/s41586-024-07487-w PMC11168924 38718835

[93] Sehnal, D. et al. Mol∗Viewer: modern web app for 3D visualization and analysis of large biomolecular structures. *Nucleic Acids Res.* **49**, W431–W437 (2021). 10.1093/nar/gkab314 PMC8262734 33956157

[94] Berman, H. M. et al. The Protein Data Bank. *Nucleic Acids Res.* **28**, 235–242 10.1093/nar/28.1.235 (2000). PMC102472 10592235

[95] Wickham, H. *ggplot2: Elegant Graphics for Data Analysis Second Edition* (Springer, 2015).

[96] Gu, Z., Eils, R. & Schlesner, M. Complex heatmaps reveal patterns and correlations in multidimensional genomic data. *Bioinformatics* **32**, 2847–2849 (2016). 10.1093/bioinformatics/btw313 27207943

