# Supplementary material for: Functional chimeric mRNAs encode proteins in mammalian immunity
Source: Nature. 2026 Sep 2;658(8137):1015–24. doi: 10.1038/s41586-026-10982-x (PMC13686932; doi:10.1038/s41586-026-10982-x)

nature portfolio

https://doi.org/10.1038/s41586-026-10982-x

Supplementary information

Functional chimeric mRNAs encode proteins in mammalian immunity

In the format provided by the authors and unedited

Supplementary Figure 1

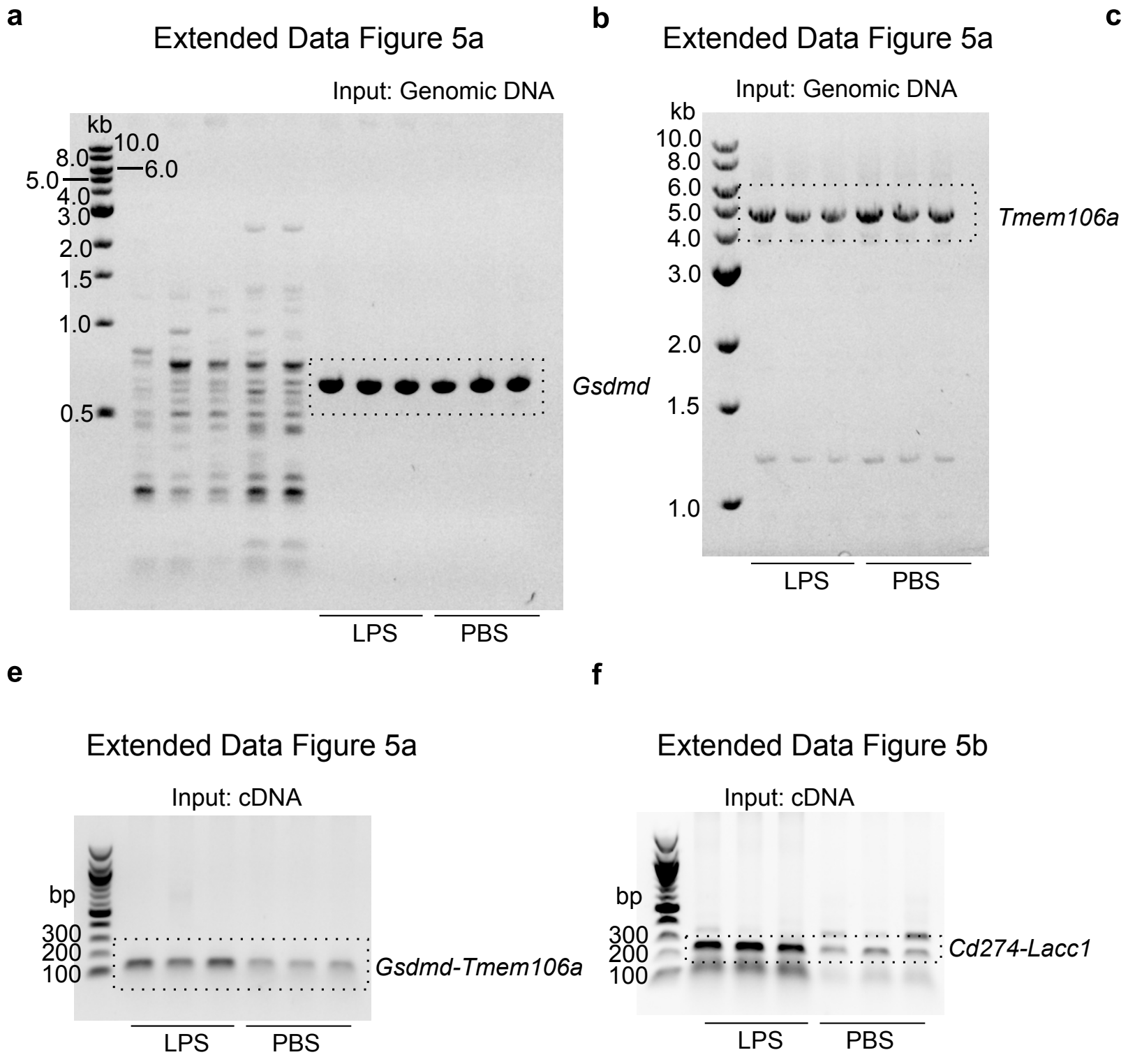

Supplementary Figure 2

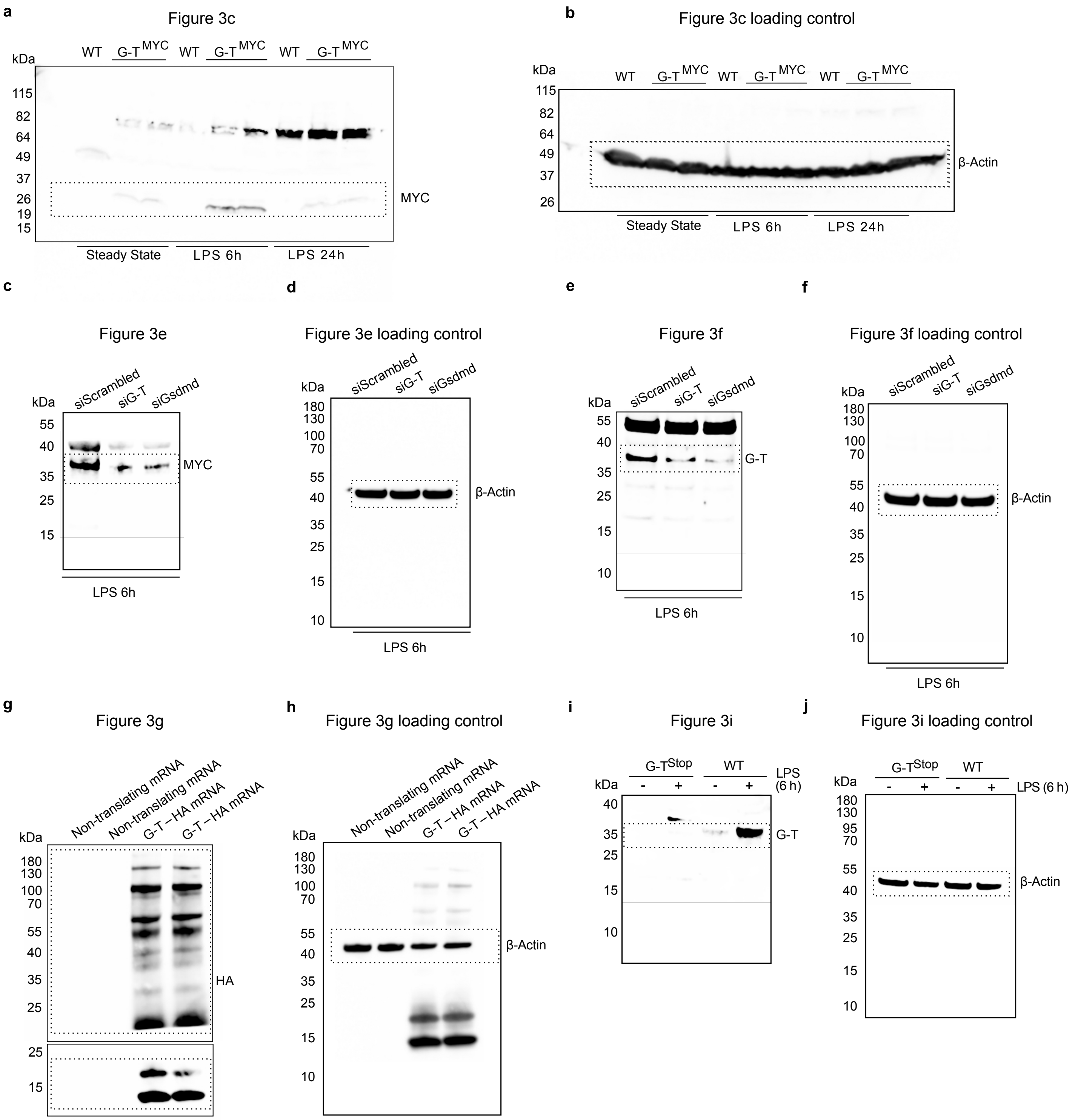

Supplementary Figure 3

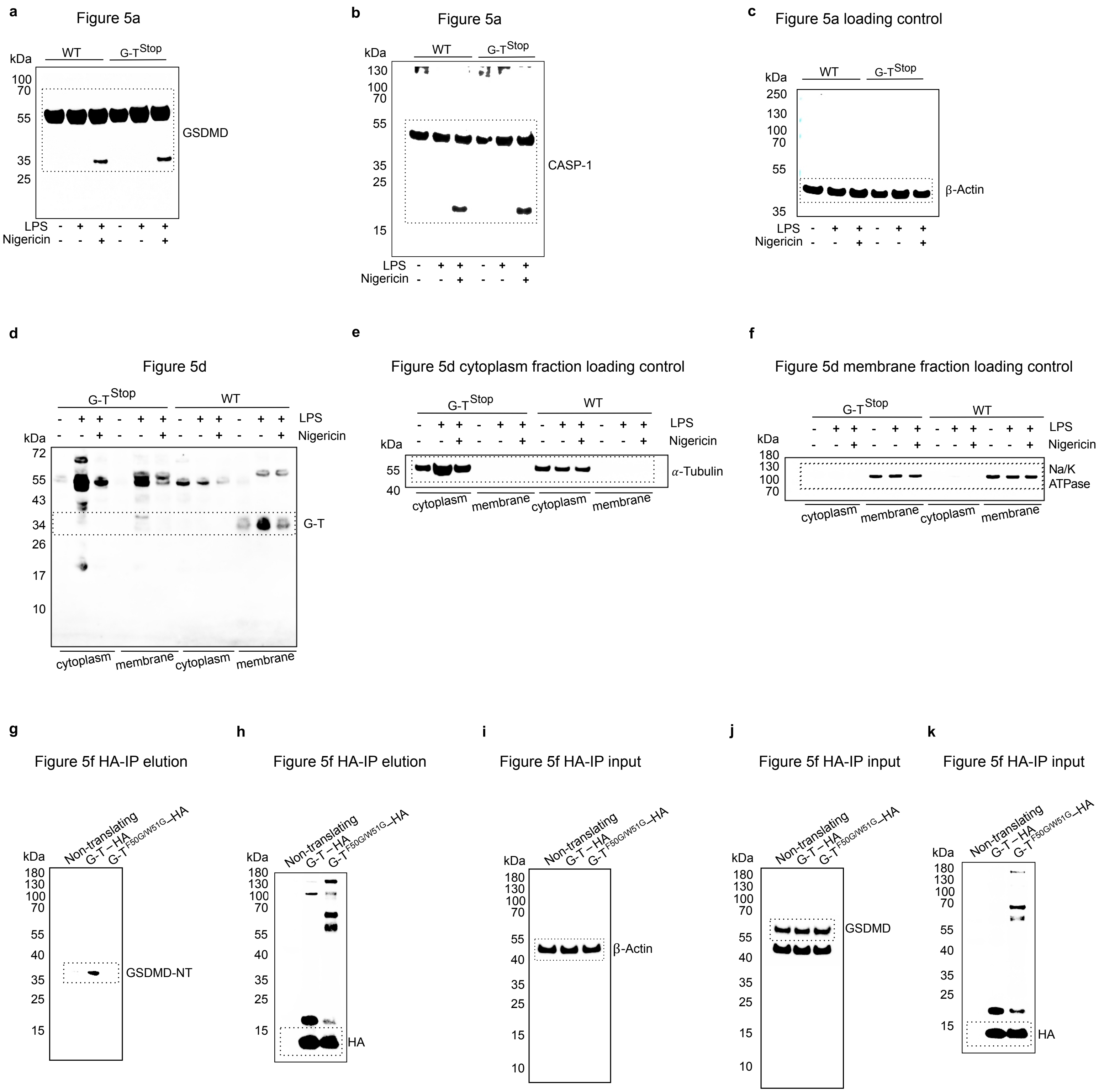

Supplementary Figure 4

a

Extended Figure 8h cleaved CASP-1

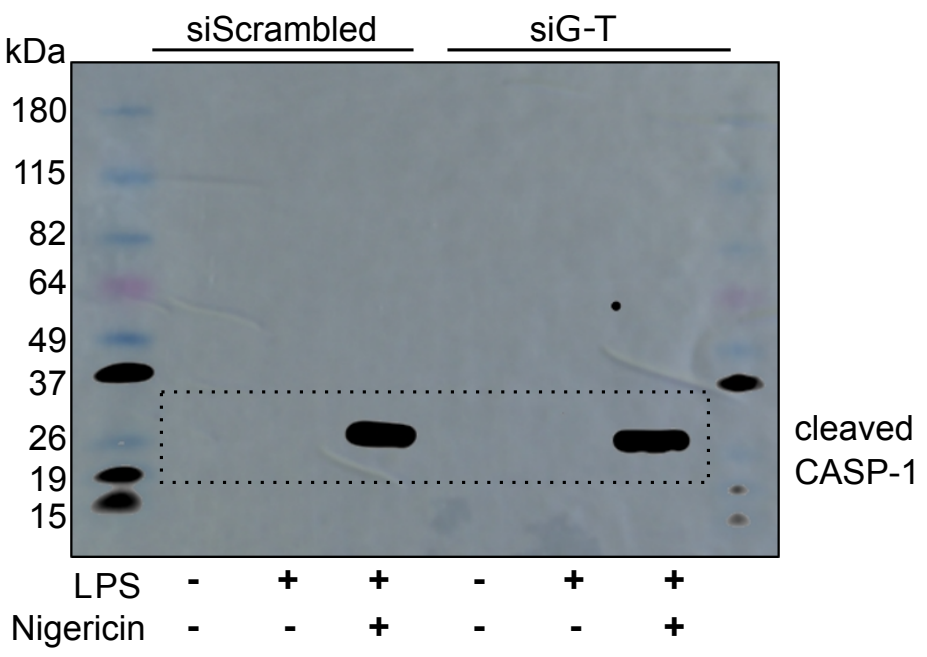

b

Extended Figure 8h cleaved GSDMD

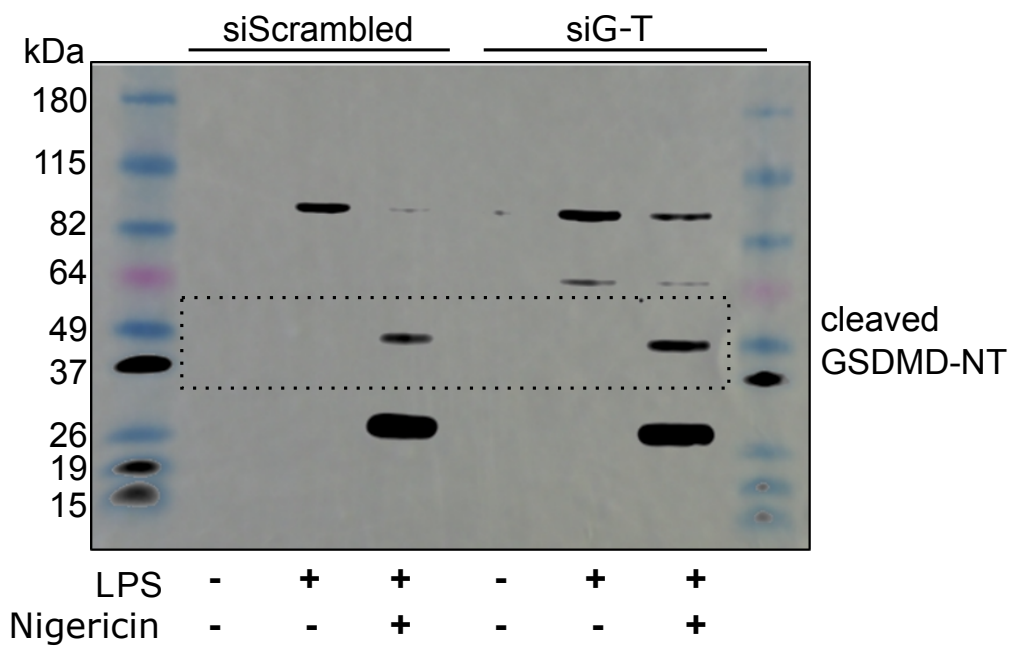

c

Extended Figure 8g loading control

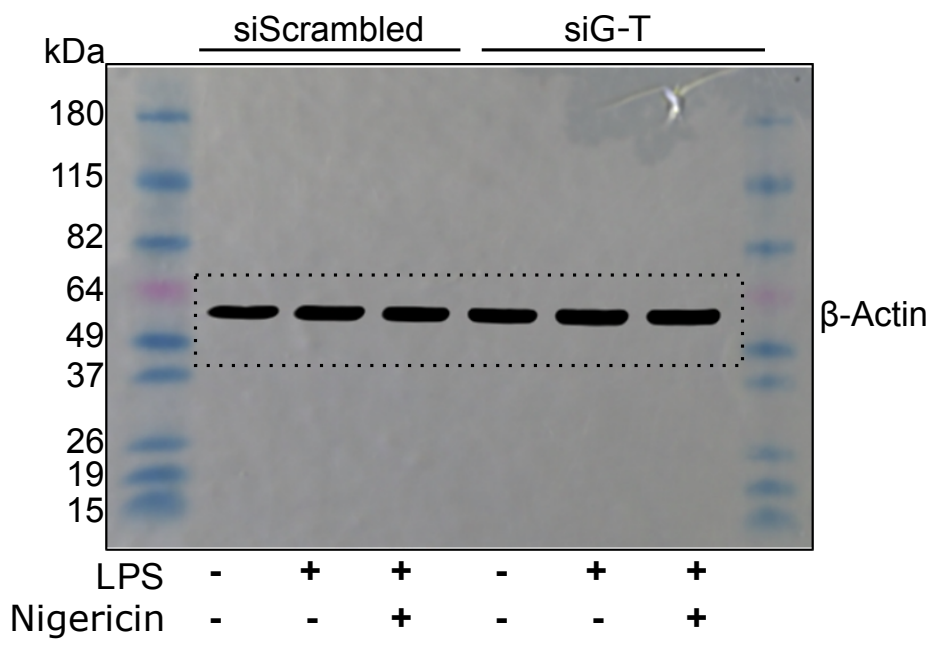

Supplementary Figure 5

a

Extended Figure 9f

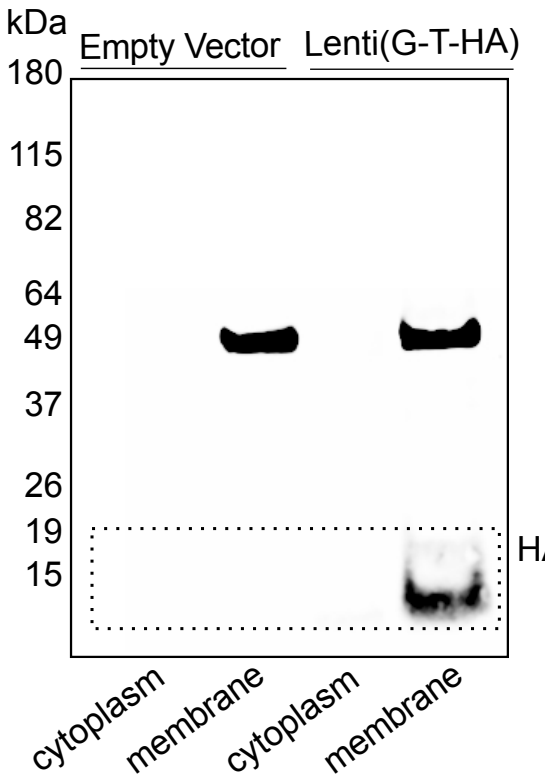

b

Extended Figure 9f cytoplasm control

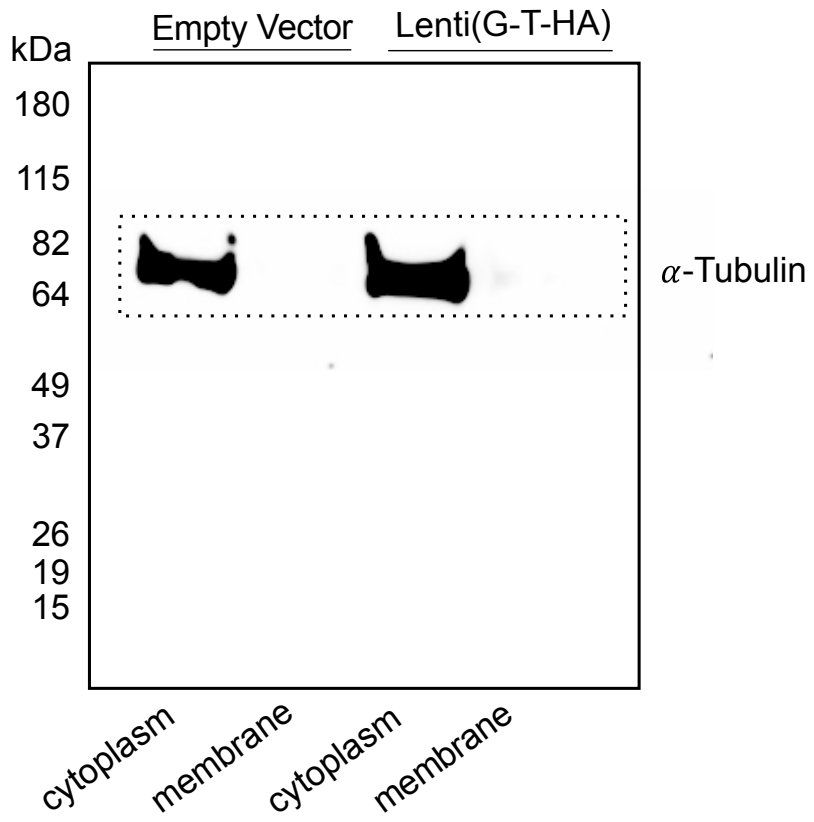

c

Extended Figure 9f membrane control

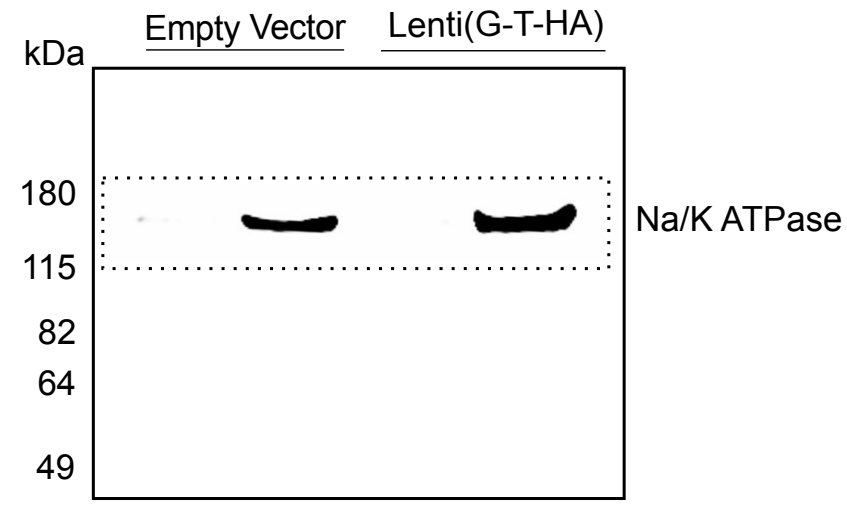

d

Extended Figure 9h

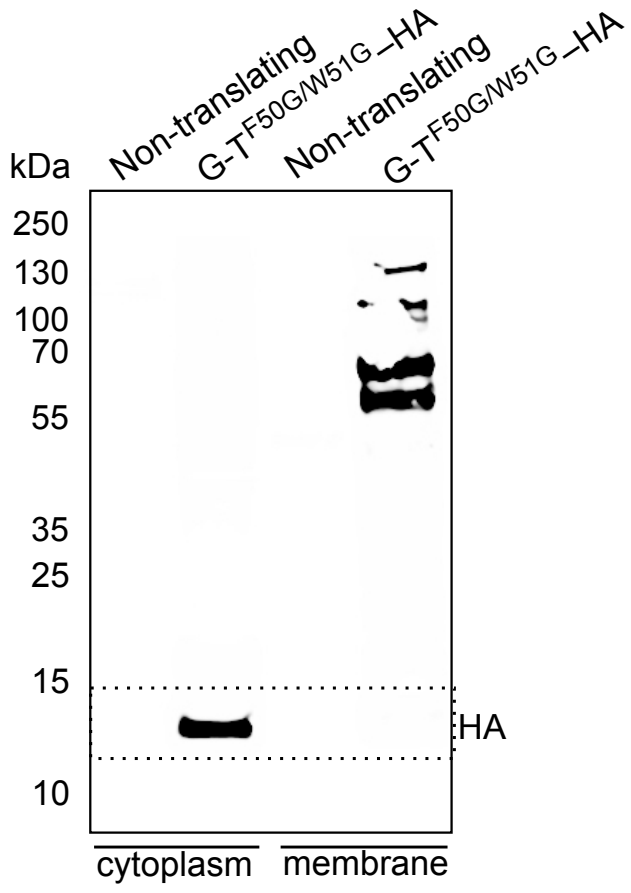

e

Extended Figure 9h cytoplasm control

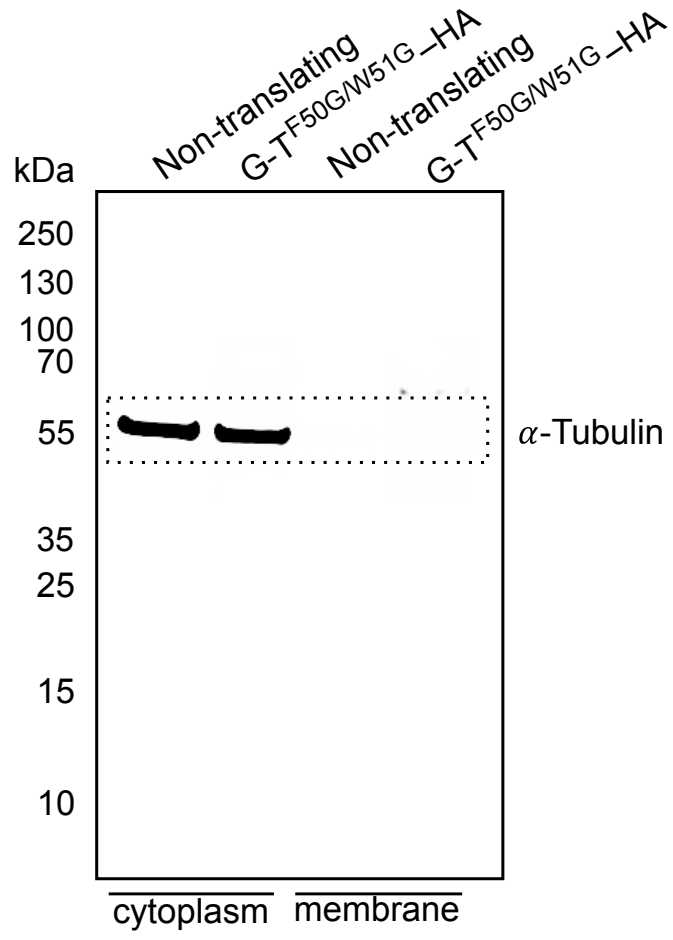

f

Extended Figure 9h membrane control

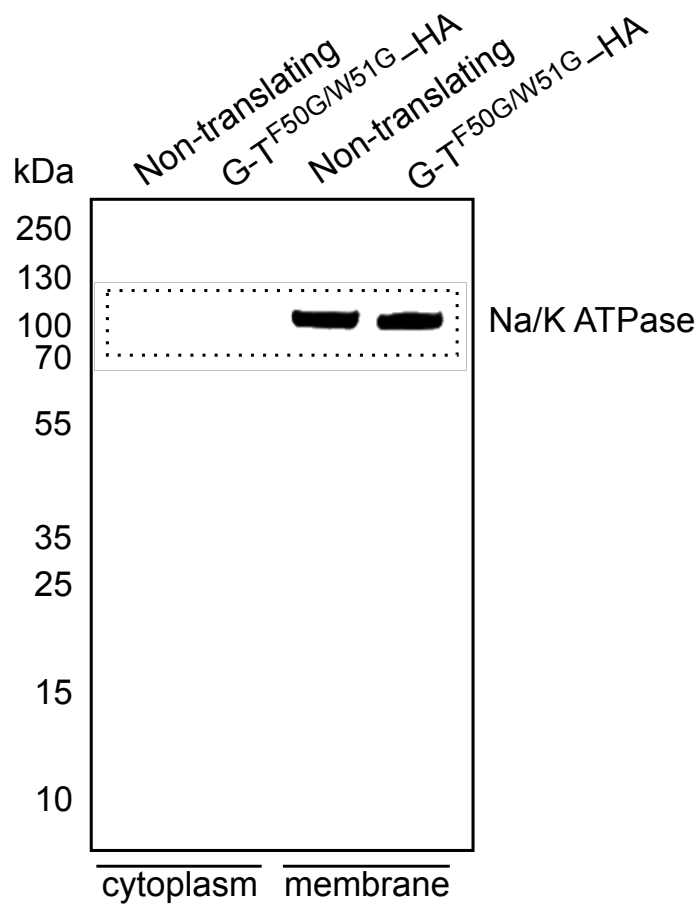

g

Extended Figure 9i

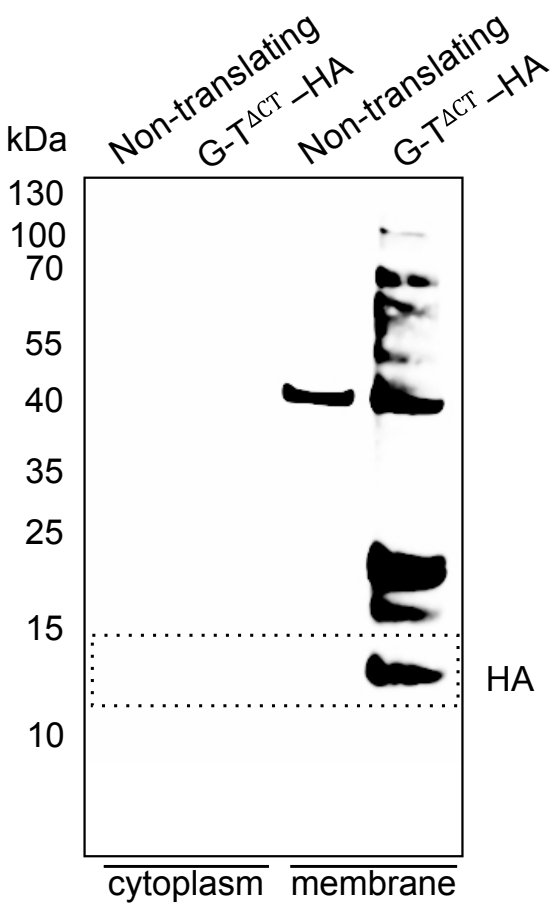

h

Extended Figure 9i fraction controls

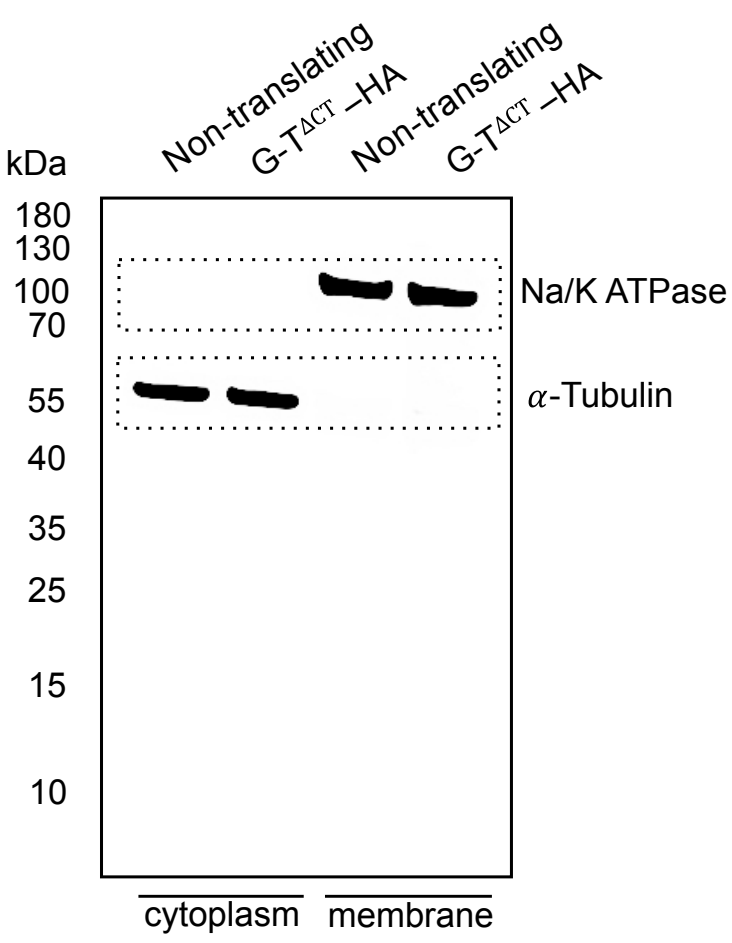

Supplement: Supplementary file 1 [file 41586_2026_10982_MOESM1_ESM.pdf]
