# Supplementary material for: Functional chimeric mRNAs encode proteins in mammalian immunity
Source: Nature. 2026 Sep 2;658(8137):1015–24. doi: 10.1038/s41586-026-10982-x (PMC13686932; doi:10.1038/s41586-026-10982-x)
Supplement: Supplementary file 2 [file 41586_2026_10982_MOESM2_ESM.pdf]

nature portfolio

Corresponding author(s): Ruaidhri Jackson

Last updated by author(s): Aug 1, 2026

Reporting Summary

Nature Portfolio wishes to improve the reproducibility of the work that we publish. This form provides structure for consistency and transparency in reporting. For further information on Nature Portfolio policies, see our Editorial Policies and the Editorial Policy Checklist.

Statistics

For all statistical analyses, confirm that the following items are present in the figure legend, table legend, main text, or Methods section.

n/a	Confirmed	
☐	☒	The exact sample size (*n*) for each experimental group/condition, given as a discrete number and unit of measurement
☐	☒	A statement on whether measurements were taken from distinct samples or whether the same sample was measured repeatedly
☐	☒	The statistical test(s) used AND whether they are one- or two-sided<br>*Only common tests should be described solely by name; describe more complex techniques in the Methods section.*
☐	☒	A description of all covariates tested
☐	☒	A description of any assumptions or corrections, such as tests of normality and adjustment for multiple comparisons
☐	☒	A full description of the statistical parameters including central tendency (e.g. means) or other basic estimates (e.g. regression coefficient) AND variation (e.g. standard deviation) or associated estimates of uncertainty (e.g. confidence intervals)
☐	☒	For null hypothesis testing, the test statistic (e.g. *F*, *t*, *r*) with confidence intervals, effect sizes, degrees of freedom and *P* value noted<br>*Give P values as exact values whenever suitable.*
☒	☐	For Bayesian analysis, information on the choice of priors and Markov chain Monte Carlo settings
☒	☐	For hierarchical and complex designs, identification of the appropriate level for tests and full reporting of outcomes
☒	☐	Estimates of effect sizes (e.g. Cohen's *d*, Pearson's *r*), indicating how they were calculated

Our web collection on statistics for biologists contains articles on many of the points above.

Software and code

Policy information about availability of computer code

Data collection	MinKNOW v22.03.4, MinKNOW Core 5.0.0, Bream v7.0.9, and Guppy v6.0.7 Oxford Nanopore Technologies software. Linux GNU Ubuntu 20.04 LTS OS.
Data analysis	GraphPad Prism 10.0.2, Microsoft Excel (Version 2607 Build 16.0.20228.20110) 64-bit, LibreOffice Calc 26.2.4.2 (X86_64), R programming language ≤ 4.5.2, R Studio ≤ 2026.05.0 Build 218, Python programming language ≤ 3.13.9, Anaconda Python distribution ≤ 26.1., AlphaFold 3 via AlphaFold Server (https://alphafoldserver.com/welcome), Mol* and RCSB PDB platform (https://www.rcsb.org/3d-view). LongGF v0.1.2, JAFFAL v2.2 and v2.3, Genion v1.2.3, BLAST+ v2.13.0, and the TYPHON wrapper script v1.0. Fastp 0.23.2, Arriba v2.4.0, FusionCatcher v1.33, STAR-Fusion v1.12.0, STAR-SEQR v0.6.7, JAFFA v2.3, and Pizzly v0.37.3. nSolver ≤ 4.0. diffHic v1.38.0, cutadapt v4.8, bowtie2 v2.5.3, the Picard suite v1.117, GENOVA ≤ v1.0.1, juicer tools v1.22.01, Rsubread ≤ 2.26.0. tximport v1.38.2, NanoPlot v1.42.0, Minimap2 v2.24, SAMtools v1.15, Salmon v1.10.2, DESeq2 v1.50.2, pygtftk v1.6.2, pafr v0.0.2, tidyverse v2.0.0, Seqkit v2.4.0, Bedops v2.4.41, Bedtools v2.31.0, Clustalo v1.2.4, Clustalw v2.1, Biopython v1.81, Seqtk v1.4, data.table v1.15.2, orfipy v0.0.4, Nichenetr v2.2.0, rtracklayer v1.64.0, kallisto v0.48.0, and scCustomize v3.2.0. ComplexHeatmap ≤ v2.26.1 and ggplot2 suite ≤ v4.0.3. Microsoft Windows 11 Pro 24H2 OS , MacOS BigSur Version 11.6, and Linux GNU Pop!_OS 22.04 and Ubuntu 26.04 OS. The TYPHON wrapper script is available at https://github.com/erenada/TYPHON.

For manuscripts utilizing custom algorithms or software that are central to the research but not yet described in published literature, software must be made available to editors and reviewers. We strongly encourage code deposition in a community repository (e.g. GitHub). See the Nature Portfolio guidelines for submitting code & software for further information.

Data

Policy information about availability of data

All manuscripts must include a data availability statement. This statement should provide the following information, where applicable:

- Accession codes, unique identifiers, or web links for publicly available datasets
- A description of any restrictions on data availability
- For clinical datasets or third party data, please ensure that the statement adheres to our policy

Long-read direct RNAseq data have been deposited in GEO under accession code GSE267147 (murine) and GSE277057 (human). Short-read RNAseq data have been deposited in GEO under accession code GSE324139. Hi-C sequencing data have been deposited in GEO under accession code GSE324391. K562 cell data was downloaded from GSE123191 (PMID: 31839405, PMID: 33514943), accessible from https://www.ncbi.nlm.nih.gov/geo/query/acc.cgi?acc=GSE123191 .

GENCODE reference files for GENCODE M28 release (GRCm39) and GENCODE 43 release (GRCh38.p13) are available at https://www.gencodegenes.org/. UCSC reference files are available at https://genome.ucsc.edu/ and can be accessed via the Table Browser at https://genome.ucsc.edu/cgi-bin/hgTables. Ensembl reference files are available at https://www.ensembl.org/info/data/ftp/index.html?redirect=no and can be downloaded via the ftp site at https://ftp.ensembl.org/pub/.

Research involving human participants, their data, or biological material

Policy information about studies with human participants or human data. See also policy information about sex, gender (identity/presentation), and sexual orientation and race, ethnicity and racism.

Reporting on sex and gender	Anonymous samples were collected and no information is available on sex and gender.
Reporting on race, ethnicity, or other socially relevant groupings	Anonymous samples were collected and no information is available on race, ethnicity or other socially relevant groupings.
Population characteristics	Anonymous peripheral blood samples were collected from the Mississippi Valley Regional Blood Center as waste cellular products. Anonymous cryopreserved CD14+ monocytes were purchased from Lonza (cat. 2W-400C).
Recruitment	No participants were recruited as part of this study.
Ethics oversight	Anonymous peripheral blood samples in this study were collected by Dr. Liang Shan's research group under the approval of the Washington University School of Medicine Internal Review Board.

Note that full information on the approval of the study protocol must also be provided in the manuscript.

Field-specific reporting

Please select the one below that is the best fit for your research. If you are not sure, read the appropriate sections before making your selection.

☒ Life sciences ☐ Behavioural & social sciences ☐ Ecological, evolutionary & environmental sciences

For a reference copy of the document with all sections, see nature.com/documents/nr-reporting-summary-flat.pdf

Life sciences study design

All studies must disclose on these points even when the disclosure is negative.

Sample size	No statistical methods were used to predetermine sample size for experimentation. Given the minimal experimental variation in BMDMs using inbred mouse strains as a cell source, a minimum of three biological replicates were used per sample. In vivo and other in vitro experiments were conducted with samples sizes consistent with the relevant literature (PMID: 26375259 and PMID: 26375003).
Data exclusions	No data were excluded.
Replication	All data were repeated successfully. In experiments using mice, each mouse was considered an individual biological replicate, and experimetns were conducted 2 or more times with multiple mice. In primary cell based studies each biological replicate was from a individual mouse bone marrow collection, and replicates were displayed as biological replicates only. For cell line based work, each experiment was conducted with cells from different frozen stock cultures and wells and reported as biological replicates. For cell free based experiments, each experimetn was conducted at least 3 times using different protein and lipid preparations.
Randomization	Wild-type littermate mice were randomly assigned to groups for lipid nanoparticle (LNP) treatment prior to LPS sepsis or Salmonella Typhimurium infection for experiments involving LNPs. Randomization was not performed for other experiments as all cells used for analysis were from the same inbred strain of mouse. Experiments were designed with respect to covariates and controlling for relevant factors in line with standard experimental practices in the relevant literature (PMID: 26375259 and PMID: 26375003).

Blinding	No blinding was utilized as part of this study. Investigators were not blinded to group allocation during the in vivo experiments. The primary outcomes, including body weight and body temperature, were objective quantitative measurements obtained using calibrated instruments and analyzed according to prespecified criteria, thereby minimizing the potential for observer-dependent bias.

Behavioural & social sciences study design

All studies must disclose on these points even when the disclosure is negative.

Study description	*Briefly describe the study type including whether data are quantitative, qualitative, or mixed-methods (e.g. qualitative cross-sectional, quantitative experimental, mixed-methods case study).*
Research sample	*State the research sample (e.g. Harvard university undergraduates, villagers in rural India) and provide relevant demographic information (e.g. age, sex) and indicate whether the sample is representative. Provide a rationale for the study sample chosen. For studies involving existing datasets, please describe the dataset and source.*
Sampling strategy	*Describe the sampling procedure (e.g. random, snowball, stratified, convenience). Describe the statistical methods that were used to predetermine sample size OR if no sample-size calculation was performed, describe how sample sizes were chosen and provide a rationale for why these sample sizes are sufficient. For qualitative data, please indicate whether data saturation was considered, and what criteria were used to decide that no further sampling was needed.*
Data collection	*Provide details about the data collection procedure, including the instruments or devices used to record the data (e.g. pen and paper, computer, eye tracker, video or audio equipment) whether anyone was present besides the participant(s) and the researcher, and whether the researcher was blind to experimental condition and/or the study hypothesis during data collection.*
Timing	*Indicate the start and stop dates of data collection. If there is a gap between collection periods, state the dates for each sample cohort.*
Data exclusions	*If no data were excluded from the analyses, state so OR if data were excluded, provide the exact number of exclusions and the rationale behind them, indicating whether exclusion criteria were pre-established.*
Non-participation	*State how many participants dropped out/declined participation and the reason(s) given OR provide response rate OR state that no participants dropped out/declined participation.*
Randomization	*If participants were not allocated into experimental groups, state so OR describe how participants were allocated to groups, and if allocation was not random, describe how covariates were controlled.*

Ecological, evolutionary & environmental sciences study design

All studies must disclose on these points even when the disclosure is negative.

Study description	*Briefly describe the study. For quantitative data include treatment factors and interactions, design structure (e.g. factorial, nested, hierarchical), nature and number of experimental units and replicates.*
Research sample	*Describe the research sample (e.g. a group of tagged Passer domesticus, all Stenocereus thurberi within Organ Pipe Cactus National Monument), and provide a rationale for the sample choice. When relevant, describe the organism taxa, source, sex, age range and any manipulations. State what population the sample is meant to represent when applicable. For studies involving existing datasets, describe the data and its source.*
Sampling strategy	*Note the sampling procedure. Describe the statistical methods that were used to predetermine sample size OR if no sample-size calculation was performed, describe how sample sizes were chosen and provide a rationale for why these sample sizes are sufficient.*
Data collection	*Describe the data collection procedure, including who recorded the data and how.*
Timing and spatial scale	*Indicate the start and stop dates of data collection, noting the frequency and periodicity of sampling and providing a rationale for these choices. If there is a gap between collection periods, state the dates for each sample cohort. Specify the spatial scale from which the data are taken*
Data exclusions	*If no data were excluded from the analyses, state so OR if data were excluded, describe the exclusions and the rationale behind them, indicating whether exclusion criteria were pre-established.*
Reproducibility	*Describe the measures taken to verify the reproducibility of experimental findings. For each experiment, note whether any attempts to repeat the experiment failed OR state that all attempts to repeat the experiment were successful.*
Randomization	*Describe how samples/organisms/participants were allocated into groups. If allocation was not random, describe how covariates were controlled. If this is not relevant to your study, explain why.*
Blinding	*Describe the extent of blinding used during data acquisition and analysis. If blinding was not possible, describe why OR explain why blinding was not relevant to your study.*

Did the study involve field work? ☐ Yes ☒ No

Reporting for specific materials, systems and methods

We require information from authors about some types of materials, experimental systems and methods used in many studies. Here, indicate whether each material, system or method listed is relevant to your study. If you are not sure if a list item applies to your research, read the appropriate section before selecting a response.

Materials & experimental systems

n/a	Involved in the study
☐	☒ Antibodies
☐	☒ Eukaryotic cell lines
☒	☐ Palaeontology and archaeology
☐	☒ Animals and other organisms
☒	☐ Clinical data
☒	☐ Dual use research of concern
☒	☐ Plants

Methods

n/a	Involved in the study
☒	☐ ChIP-seq
☒	☐ Flow cytometry
☒	☐ MRI-based neuroimaging

Antibodies

Antibodies used

Anti-HA antibody (clone C29F4) (Cell Signaling Technology cat. 3724S) (1:1000); and anti-rabbit HRP-linked secondary antibody (Cell Signaling Technology cat. 7074S) (1:3000); Anti-β-Actin antibody (clone 13E5) (Cell Signaling Technology cat. 4970) (1:1000); Anti-α-Tubulin antibody (clone DM1A) (Cell Signaling Technology cat. 3873) (1:1000); Anti-NA,K-ATPase antibody (Cell Signaling Technology cat. 3010) (1:1000); anti-Myc antibody (clone 9B11) (Cell Signaling Technology cat. 2276S) (1:1000); anti-mouse HRP-linked secondary antibody (Cell Signaling Technology cat. 7076S) (1:3000); anti-cleaved gasdermin D antibody (clone E3E3P) (Cell Signaling Technology cat. 10137); anti-cleaved caspase-1 antibody (clone E2G2I) (Cell Signaling Technology cat. 89332S) (1:1000); anti-Caspase-1 (clone E9R2D) (Cell Signaling Technology, 83383); anti- Gasdermin D antibody (clone E9S1X) (Cell Signaling Technology, 39754) (1:1000); anti-HA antibody (clone 16B12) conjugated to PE (1:500) (BioLegend cat. 901518); Monoclonal ANTI-FLAG® M2-Peroxidase (HRP) antibody (clone M2) (Sigma cat. A8592) (1:1000). We also generated a custom antibody for GSDMD:TMEM106A (1:500). Briefly, rabbits were immunized with peptide antigen CGRPGHQQPPPAHRPIGQ and antibody purified from serum. Magnetic beads conjugated to an anti-HA monoclonal antibody (clone 2-2.2. 14) (ThermoFisher Scientific cat. 88836) were used for immunoprecipitation.

Validation

The GSDMD:TMEM106A antibody was validated using siRNA knockdown of Gsdmd:Tmem106a and Gsdmd. We also validated that the band was not present in Gsdmd:Tmem106a KO cells. All other antibodies used are commercially available and validated antibodies. No additional validation was performed for commercially available antibodies.

Eukaryotic cell lines

Policy information about cell lines and Sex and Gender in Research

Cell line source(s)

GSDMD KO iBMDMs were obtained from the Kagan lab, who generated these cells (PMID: 29195811). Dox inducible N terminal Gsdmd iBMDMs were also obtained from the Kagan lab, who generated these cells (PMID: 34289345). Expi293 cells were obtained from the Wu Lab (PMID: 38599239), originally sourced from Thermo Fisher Scientific.

Authentication

Cell lines were authenticated by the Kagan Lab (PMID: 29195811, PMID: 34289345) or Wu Lab (PMID: 38599239) as per their cited publications. No additional cell authentication was performed.

Mycoplasma contamination

Cell lines were not tested for Mycoplasma.

Commonly misidentified lines
(See ICLAC register)

No commonly misidentified cell lines were used in this study.

Animals and other research organisms

Policy information about studies involving animals; ARRIVE guidelines recommended for reporting animal research, and Sex and Gender in Research

Laboratory animals

Most studies were conducted using C57BL/6J mice (#000664). Some experiments utilized BALB/cJ (#000651) , WSB/EiJ (#001145), C57BL/6 N-Gsdmdem4Fcw/J (#032410), G:TMyc and G:TStop mice. G:TMyc and G:TStop mice were generated on the C57BL/6J background. Mice were used at ages 8-12 weeks old. Mice were housed in temperature (22 ± 2 °C) and humidity (55 ± 5%) controlled care facilities at Harvard Medical School on a 12 hour light:dark cycle and provided with freely available food and water. Littermate and sex matched control animals were used in all studies.

Wild animals

This study did not use wild animals.

Reporting on sex	Experiments were conducted on male and female mice.
Field-collected samples	No field collected samples were used in the study.
Ethics oversight	Harvard Medical School Animal Care and Use Committee.

Note that full information on the approval of the study protocol must also be provided in the manuscript.

Plants

Seed stocks	*Report on the source of all seed stocks or other plant material used. If applicable, state the seed stock centre and catalogue number. If plant specimens were collected from the field, describe the collection location, date and sampling procedures.*
Novel plant genotypes	*Describe the methods by which all novel plant genotypes were produced. This includes those generated by transgenic approaches, gene editing, chemical/radiation-based mutagenesis and hybridization. For transgenic lines, describe the transformation method, the number of independent lines analyzed and the generation upon which experiments were performed. For gene-edited lines, describe the editor used, the endogenous sequence targeted for editing, the targeting guide RNA sequence (if applicable) and how the editor was applied.*
Authentication	*Describe any authentication procedures for each seed stock used or novel genotype generated. Describe any experiments used to assess the effect of a mutation and, where applicable, how potential secondary effects (e.g. second site T-DNA insertions, mosiacism, off-target gene editing) were examined.*
